# Diketo[*n*]CPPs as chiral and shape-adaptive fullerene hosts and precursors to DBP[*n*]CPPs

**DOI:** 10.1039/d5sc05305f

**Published:** 2026-01-26

**Authors:** Xiaoshuang Xiang, Mathias Hermann, Lei Ye, Philipp Seitz, Lilian Estaque, Grégory Pieters, Thomas Drewello, Birgit Esser

**Affiliations:** a Institute of Organic Chemistry II and Advanced Materials, Ulm University Albert-Einstein-Allee 11 89081 Ulm Germany birgit.esser@uni-ulm.de; b Physical Chemistry I, Department of Chemistry and Pharmacy, Friedrich-Alexander-Universität Erlangen-Nürnberg Egerlandstraße 3 91058 Erlangen Germany; c Université Paris-Saclay, CEA, Département Médicaments et Technologies pour la Santé (DMTS), SCBM 91191 Gif-sur-Yvette France

## Abstract

Conjugated nanohoops, such as [*n*]cycloparaphenylenes ([*n*]CPPs) and derivatives, exhibit unique structural and optoelectronic properties, making them promising candidates for applications in optoelectronic materials, and as hosts for supramolecular chemistry. Using π-systems unsymmetric to rotation or incorporating chiral units can furnish chiral nanohoops. We herein present the synthesis and characterization of diketo[8]- and diketo[9]CPPs, along with their corresponding dibenzo[*a*,*e*]pentalene (DBP) derivatives, DBP[8]- and DBP[9]CPP. Due to the central chirality of the diketone-units, these nanohoops are chiral without the possibility of racemization through rotation and show distinct chiroptical properties. The diketo[*n*]CPPs possess high fluorescence quantum yields of 87% (*n* = 8) and 92% (*n* = 9). The shape-adaptive properties of diketo[*n*]CPPs, facilitated by the tunable kink angle of the diketo unit, enable efficient host–guest interactions with fullerenes. Fluorescence titration revealed a similar binding constant for both fullerenes C_60_ and C_70_ (5 × 10^4^ to 7 × 10^4^ M^−1^ in toluene), corroborated by DFT calculations that illustrate adaptive changes in nanohoop geometry upon fullerene complexation. ESI-MS is employed to generate ionized [1 : 1] host–guest complexes of diketo[9]CPP and DBP[9]CPP with C_60_ and C_70_ as guests. The relative stabilities of these complexes are evaluated in energy-resolved collision experiments.

## Abstract 2

Diketo[8]CPP and diketo[9]CPP are chiral conjugated nanohoops with high photoluminescence quantum yields and shape-adaptive fullerene binding.

## Graphical abstract

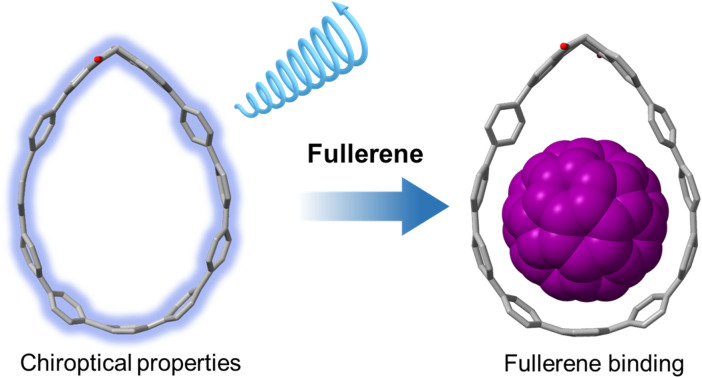

## Introduction

[*n*]Cycloparaphenylenes (CPPs) are radially oriented π-conjugated nanohoops composed of *para*-linked benzene, possessing strikingly distorted phenylene moieties. Their unique physical and chemical properties make them promising materials for various fields, such as materials science, electronics, and biological applications.^1–4^ Various aromatic subunits other than benzene were introduced to tune the structural or optoelectronic properties of conjugated nanohoops beyond the size-dependent effect known for [*n*]CPPs.^5–8^ To date, a variety of nanohoops and their derivatives have been synthesized. These structures are a family of shape-rigid conjugated macrocycles featuring shape-persistent cavities surrounded by radially oriented π-systems. With these unique properties, conjugated nanohoops can be excellent hosts for fullerene guest molecules with high binding affinity.^9–11^ However, only a few examples have been reported to date on shape-adaptive nanohoops. Cong and coworkers reported a CPP-based figure-of-eight-shaped host enabling a similar guest-adaptive encapsulation of fullerenes.^12^ Stępień and coworkers published a squid-shaped molecule with a flexible cavity that binds neutral and cationic guests.^13^ Introducing flexible host structures can enhance guest selectivity and promote the dynamic binding of guest molecules, resulting in interesting host–guest properties and providing new insights into research areas such as guest recognition and separation.^14–17^

**Fig. 1:**
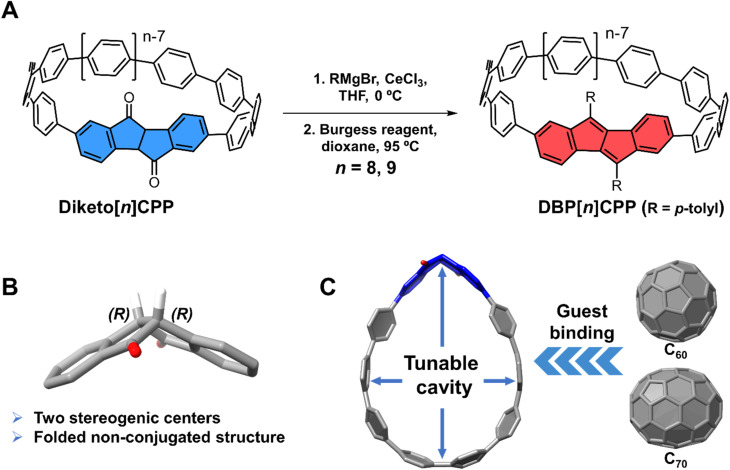
(A) Diketone- and DBP-based chiral nanohoops synthesized herein; (B) unique structure of (*R*,*R*)-dihydroindeno[2,1-*a*]indene-5,10-dione as the central and chiral diketone unit; (C) the shape-adaptive cavity of diketo[8]- and diketo[9]CPPs enables binding of fullerenes. All structures were optimized with B3LYP-D3(BJ)/6-31G(d).

Previously, we described the synthesis of diketo[6]CPP and diketo[7]CPP and the corresponding DBP[6]CPP and DBP[7]CPP (with R = mesityl, Fig. 1A).^18^ Diketo[*n*]CPPs are chiral based on the central chirality of the dihydroindeno[2,1-*a*]indene-5,10-dione unit (blue in Fig. 1A, structure in Fig. 1B). When the diketo units are transformed into dibenzo[*a*,*e*]pentalenes (DBPs), DBP[*n*]CPPs result, which are still chiral based on the bend in the DBP panel – as long as its rotation through the nanohoop is sterically hindered, which is the case for *n* = 6, 7, and also for *n* = 8, 9 reported herein. The enantiomers of diketo[*n*]CPPs can therefore be defined using (*S*,*S*)- or (*R*,*R*)-descriptors (Fig. 1B), while for the DBP[*n*]CPPs the M- and P-notations are suited.^18^ While both diketo[6]- and -[7]CPPs were highly fluorescent, leading to circularly polarized luminescence, their cavities were too small to host fullerene guest molecules. Building upon this work, we herein report on larger nanohoops diketo[8]- and diketo[9]CPPs as well as the derived DBP[8]- and DBP[9]CPPs, which we investigated for potential utilization in the fields of circularly polarized luminescence (CPL) materials and supramolecular chemistry. The cavities of diketo[8]- and diketo[9]CPP are large enough as well as shape-adaptive, so that they can both host C_60_ or C_70_ fullerene (Fig. 1C). Notably, the results of solution studies reveal that both diketo[*n*]CPPs have a similar binding affinity for C_70_ and C_60_, demonstrating that the molecules exhibit adaptive binding to these fullerenes. In addition, diketo[8]- and [9]CPPs show high fluorescence quantum yields of 0.87 and 0.92, respectively, which are enhanced compared to the smaller diketo[6]- and [7]CPPs (0.57 and 0.66, respectively).

## Results and discussion

### Synthesis

**Scheme 1:**
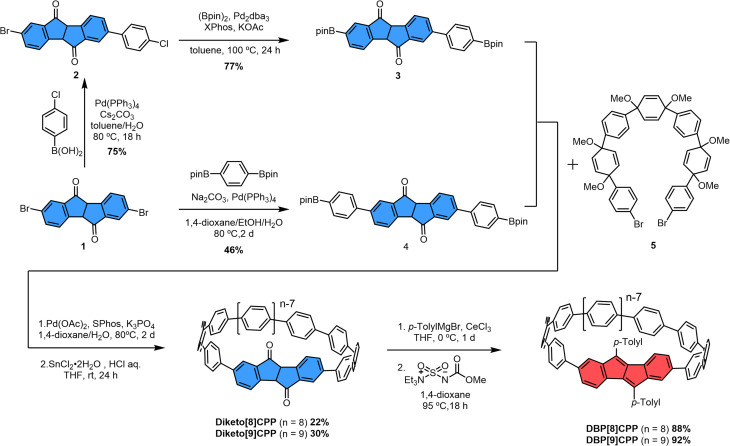
Synthesis of diketo[*n*]CPPs and DBP[*n*]CPPs (*n* = 8, 9).

The synthesis of the diketo[*n*]CPPs and DBP[*n*]CPPs begins with the preparation of the key intermediates (Scheme 1). The bis-brominated kinked diketone 1 is a key building block in our synthetic route.^19^ In order to access larger diketo[*n*]CPPs in comparison to our previous studies,^18,20^ we extended this building block by attaching one (2, 3) or two (4) further aryl units. The Suzuki–Miyaura coupling of dibromo–diketone 1 with 1.1 equivalents of 2-chlorophenylboronic acid afforded monoarylated diketone 2 in a yield of 75%. Then, the following Miyaura-borylation furnished compound 3 in a good yield of 77%. Separately, dibromo–diketone 1 was transformed into the π-extended bis(boronic ester) 4 by Pd-catalyzed borylation in a yield of 46%. Subsequently, the macrocyclization of these intermediates (3 and 4) with C-shaped unit 5 (ref. 20 and 21) was carried out using a Pd-catalyzed Suzuki–Miyaura coupling reaction. This was followed by a reductive aromatization and furnished the larger diketo[*n*]CPPs in good yields of 22% (*n* = 8) and 30% (*n* = 9) over both steps.

In the next step, the diketo[*n*]CPPs were transformed into DBP[*n*]CPPs, for which we chose *p*-tolyl substituents. CeCl_3_-mediated Grignard addition of *p*-tolyl magnesium bromide was followed by two-fold water elimination with the Burgess reagent as a mild dehydration method,^19,22^ which provided DBP[8]CPP and DBP[9]CPP in high yields of 88% and 92%, respectively, over these two steps.

DFT calculations at the B3LYP/6-31G(d) level of theory using the StrainViz^23^ tool provided information on the strain energies of these nanohoops (see SI Section 9.1 for details). The strain energies amount to 42 and 41 kcal mol^−1^ for diketo[8]CPP and diketo[9]CPP, and 59 and 53 kcal mol^−1^ for DBP[8]CPP and DBP[9]CPP, respectively. These results show that the diketo[*n*]CPPs have lower strain energies than their corresponding DBP[*n*]CPPs, which is likely due to the naturally kinked structure of the diketo-units in the formed nanohoops, relieving part of the strain (see SI Table S54 and Fig. S63). Compared to the smaller diketo[6]CPP (strain energy 52 kcal mol^−1^),^18^ diketo[8]CPP and diketo[9]CPP are significantly less strained. Diketo[*n*]CPPs and DBP[*n*]CPPs exhibit comparable or lower strain than conventional [*n*]CPPs^23^ and *m*[*n*]CPPs^24^ with the same number of phenylenes (see SI Fig. S64). Notably, the synthesis of smaller nanohoops requires overcoming substantially larger strain energies.

### Structural properties

The structures of all nanohoops and their intermediates were characterized by nuclear magnetic resonance (NMR) spectroscopy, high-resolution mass spectrometry (HRMS), and partly by single-crystal X-ray diffraction (SC-XRD) (see the SI for details). The structures of DBP[8]CPP and DBP[9]CPP were further confirmed by 2D NMR, which enabled full assignment of the DBP panel and *p*-tolyl substituents (Fig. S25 and S26). Notably, the diketo[*n*]CPPs and DBP[*n*]CPPs were stable under ambient conditions and could be handled without any precautions.

The structures of both diketo[*n*]CPPs (*n* = 8, 9) and DBP[9]CPP were unambiguously confirmed by SC-XRD. Crystals were grown by vapor diffusion of acetonitrile into a dichloroethane solution for diketo[8]CPP and by vapor diffusion of acetonitrile into a THF solution for diketo[9]CPP. We were also able to obtain single crystals of DBP[9]CPP by vapor diffusion of *n*-hexane into a CHCl_3_ solution.

**Fig. 2:**
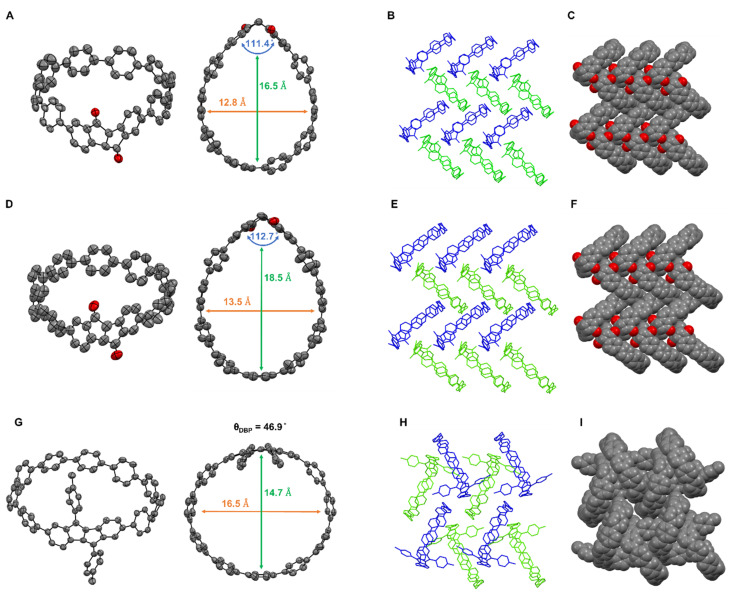
Single-crystal X-ray structural analysis of diketo[8]CPP (A–C), diketo[9]CPP (D–F) and DBP[9]CPP (G–I). (A, D and G) Side view and top view of the crystal structures of diketo[9]CPP and DBP[9]CPP; (B, C, E, F, H and I) packing structure. (*S*,*S*) or (*R*,*R*) for the diketo[*n*]CPPs and M- or P- for the DBP[9]CPP enantiomers are highlighted in blue and green color, respectively. Solvent molecules and hydrogen atoms are omitted for clarity. Thermal ellipsoids are shown at the 50% probability level.

Diketo[*n*]CPPs exhibit a waterdrop shape (Fig. 2A and D) caused by the kinked diketone unit. The cavity widths of diketo[8]CPP and diketo[9]CPP are 12.8 Å and 13.5 Å, respectively, close to the diameter of [10]CPP (13.7 Å).^25^ The “kink” angles of the diketone units amount to 111.4° (diketo[8]CPP) and 112.7° (diketo[9]CPP), which are larger than that of the “free” diketone 1 (107.2°).^19^ These angles are close to those reported for diketo[6]CPP (110.3°).^18^ Hence, in order to relieve strain in the oligoparaphenylene loop, the diketone kink angles are widened in diketo[*n*]CPPs.

As shown in Fig. 2G, DBP[9]CPP exhibits an oval shape in the solid state, with similar internal cavity diameters of 16.5 Å and 14.7 Å in two opposing directions. The DBP moiety in DBP[9]CPP is distorted and deviates from planarity. The bending of the DBP unit can be quantified by the bending angle *Θ*_DBP_,^19^ which is 46.9° in this case. This distortion is lower than those reported for DBP[6]- and DBP[7]CPPs.^18^

For all three nanohoops, the solid-state packing occurred in racemic form with the enantiomers stacked in alternate columns (see blue and green molecules in Fig. 2B, E, and H). For diketo[*n*]CPPs, the packing of the molecules in the solid state occurs in a herringbone fashion with the formation of head-to-head structures (Fig. 2B and C). The diketone units point towards each other, which leads to hydrogen bonding with the oxygen atoms in addition to C–H⋯π-interactions (see SI Section 8.2). For DBP[9]CPP, we observe a one-dimensional nested structure, where the tolyl side group of a P (or M) enantiomer inserts into the cavity of an adjacent M (or P) enantiomer, and the insertion propagates sequentially (Fig. 2H and I).

### Photophysical properties

**Fig. 3:**
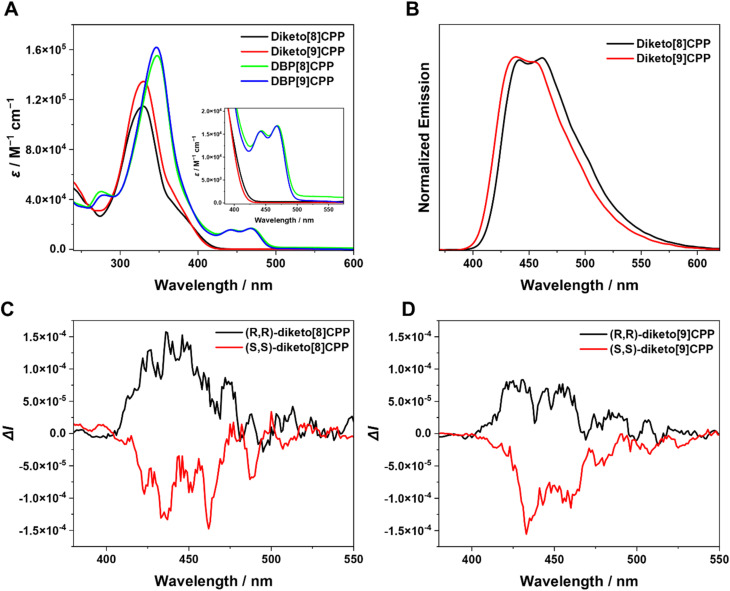
(A) Absorption (10^−5^ M to 10^−6^ M) and (B) photoluminescence spectra (10^−6^ M to 10^−7^ M) of diketo[*n*]CPPs and DBP[*n*]CPPs; CPL spectra of (C) diketo[8]CPP and (D) diketo[9]CPP (5 × 10^−5^ M). All measurements were carried out in dichloromethane.

The photophysical properties of diketo[*n*]CPPs and DBP[*n*]CPPs were studied by UV/vis absorption spectroscopy and steady-state fluorescence spectroscopy (Fig. 3 and Table 1). The absorption spectra of diketo[*n*]CPPs and DBP[*n*]CPPs each show a strong band corresponding to the absorption of the oligoparaphenylene (PP) linkers as their maximum between 329 and 347 nm (Fig. 3A). Based on the results of TD-DFT (B3-LYP-D3(BJ)/def2-TZVP) calculations, these peaks correspond to multiple orbital transitions (see SI Tables S7–S10). For diketo[*n*]CPPs the main absorption bands appear at wavelengths close to diketo[6]- and [7]CPPs due to their similar orbital energy levels (Table 1 and SI Fig. S66). In addition, a weak shoulder absorption band at 360–420 nm can also be observed, which can be assigned to the HOMO → LUMO transition (SI Tables S7 and S8). The signature bands of the DBP units^26–28^ in the DBP[*n*]CPPs appear as peaks around 440–480 nm and a red-shifted long-wavelength-absorption onset compared to the diketo[*n*]CPPs. These peaks correspond to the HOMO → LUMO (first peak) and HOMO−1 → LUMO (second peak) transitions (see SI, Tables S9 and S10). Compared to DBP[6]- and [7]CPPs, the DBP bands are more expressed in the larger DBP[8]- and [9]CPPs. The DBP[*n*]CPPs (*n* = 6–9) show higher molar attenuation coefficients (*ε*) than the diketo[*n*]CPPs. For all nanohoops, *ε* increases with the number of benzene rings (*n*), exhibiting a similar trend to [*n*]CPPs.^2^ The molar attenuation coefficients *ε* exhibit a positive correlation with increasing hoop size and conjugation (see values in Table 1).

**Table 1:** Optical properties^a^ and frontier molecular orbital energy levels of diketo[*n*]CPPs and DBP[*n*]CPPs

Compound	*λ*_abs-max_/nm	*ε*/M^−1^ cm^−1^	*λ*_em-max_ c/nm	*Φ*_F_	Δ*E*_opt_^e^/eV	*E*_LUMO_ d/eV	*E*_HOMO_ d/eV	|*g*_lum_|
Diketo[6]CPP^18^	325	6.4 × 10^4^	481	0.57	—	−1.98	−5.94	4.0 × 10^−4^
Diketo[7]CPP^18^	329	8.5 × 10^4^	472	0.66	—	−2.01	−5.96	—
Diketo[8]CPP	329	1.1 × 10^5^	460	0.87	2.97	−1.96	−5.95	6.8 × 10^−4^
Diketo[9]CPP	330	1.3 × 10^5^	437	0.92	3.01	−2.02	−5.95	6.9 × 10^−4^
DBP[8]CPP^b^	347	1.5 × 10^5^	437	<0.01	2.51	−2.46	−5.54	—
DBP[9]CPP^b^	346	1.6 × 10^5^	452	<0.01	2.53	−2.45	−5.58	—

a In dichloromethane.

b Substituent: *p*-tolyl.

c Excitation wavelength = 330 nm.

d HOMO and LUMO levels calculated at the PW6B95-D3(BJ)/def2-QZVPP level of theory.

e Determined from the onset of the longest wavelength absorption.

All four nanohoops are fluorescent with emission maxima from 437–460 nm (Fig. 3B, see SI Fig. S35 for DBP[8]- and -[9]CPPs, for exact values see Table 1). These emissions stem from the oligo(paraphenylene) moieties, which explains the similar wavelengths and shapes of the emission bands. Compared to the smaller congeners with *n* = 6 and 7,^18^ the emission maxima are blue-shifted both in the diketo- and DBP[8]- and [9]CPPs. This is in line with observations on [*n*]CPPs, where the emission blue-shifts with increasing size, a remarkable size-dependent effect, which stands in contrast to linear conjugated PPs.^1^ The multiple emission peaks likely arise from vibronic progressions of the lowest singlet excited state rather than from multiple emissive electronic states. Such vibronically resolved fluorescence is typical for [*n*]CPPs, where the 0–1/0–0 vibronic peak ratio has been explicitly analyzed^29^ and strong electron–vibration coupling has been reported.^30^ Remarkably, compared to the smaller diketo[*n*]CPPs (*n* = 6, 7), diketo[8]CPP and diketo[9]CPP exhibited higher fluorescence quantum yields (*Φ*_F_) of 0.87 and 0.92, respectively (for *n* = 6, *Φ*_F_ = 0.57, and for *n* = 7, *Φ*_F_ = 0.66).^18^ An increase in *Φ*_F_ with hoop size is also observed for [*n*]CPPs^2^ and *m*[*n*]CPPs.^24^ In contrast, DBP[8]CPP and DBP[9]CPP showed low *Φ*_F_ values (<0.01), which we ascribe to an energy or electron transfer occurring in the excited state from the PP units to the non-emissive DBP part of the compounds, which quenches their fluorescence. Pentalene derivatives have been shown to undergo rapid decay from the S_1_ state *via* singlet fission.^31^ Structurally similar monobenzopentalenes also show no S_1_ emission, which has been attributed to a formally forbidden S_1_ → S_0_ transition and fast intersystem crossing or singlet fission.^32^ Emission has only been observed from the S_2_ state, with extremely low quantum yields (<0.1%). Consequently, the DBP units are non-emissive and dissipate the energy radiation-free.^27^ Hence, the optical properties of the diketo[*n*]CPPs are dominated by the PP linkers with excellent fluorescence quantum yields. In DBP[*n*]CPPs, the optical properties reflect the presence of both the PP linkers in characteristic absorption and emission bands as well as that of the DBP units in a red-shifted absorption onset and an almost complete quenching of the fluorescence intensity.

Next, we studied the chiroptical properties of the nanohoops. The (*S*,*S*)- and (*R*,*R*)-enantiomers of diketo[8]- and [9]CPPs were separated using HPLC on a chiral stationary phase. The CD and CPL spectra for all compounds are shown in Fig. 3C or SI Section 6. Each pair of enantiomers displays mirror-imaged CD and CPL spectra with opposite Cotton effects. Time-dependent density functional theory (TD-DFT) calculations indicate that the theoretical CD spectra for diketo[*n*]CPPs are compatible with the experimental results (Fig. S38), from which we assigned the enantiomers. In the excited state, all enantiomers exhibit distinct CPL spectra, with dissymmetry factors |*g*_lum_| at *λ*_em_ (max) reaching 6.8 × 10^−4^ and 6.9 × 10^−4^ for diketo[8]CPP and diketo[9]CPP, respectively—values slightly higher than those reported for diketo[6]CPP.^18^ Although these dissymmetry factor values fall within the typical range for chiral organic emitters, the relatively high molar extinction coefficient and quantum yield of the two keto-nanohoops result in valuable CPL brightness values (B_CPL_)^33^ of 32.5 and 41.2 M^−1^ cm^−1^ for diketo[8]CPP and diketo[9]CPP, respectively.

We then attempted to separate the enantiomers of DBP[8]CPP, however, unsuccessfully. Reinjection of an initially pure enantiomer of DBP[8]CPP stored at room temperature for several minutes already revealed the presence of the opposite enantiomer, indicating rapid racemization under ambient conditions. Furthermore, enantiomeric separation of DBP[9]CPP showed a slight plateau between the two peaks in the HPLC elugram. This chromatographic behavior was observed even at a column-oven temperature as low as 20 °C, indicating a low racemization barrier (Fig. S39). Thus, the racemization behavior of DBP[9]CPP was investigated by means of dynamic variable-temperature (VT) HPLC on a chiral stationary phase.^34^ The elution profiles of DBP[9]CPP in the temperature range from 283 to 303 K in steps of 5 K were recorded and analyzed with the DCXplorer software packages (see SI Section 6.2).^35^ With increasing column temperature, the retention time separation between the two enantiomers became smaller, indicating a faster exchange rate. The data were then fitted using the Eyring equation to give the thermodynamic parameters Δ*H*_e_‡ = 18.6 kcal mol^−1^ and Δ*S*^‡^_e = _−31.6 J mol^−1^. Therefore, the interconversion energy barrier between the enantiomers was estimated to be 20.9 kcal mol^−1^ at 298 K, which is close to the DFT-calculated barrier (19.9 kcal mol^−1^). For DBP[8]CPP, the elution profile was then analyzed *via* the unified equation for dynamic chromatography,^35^ which enabled the determination of a racemization barrier of Δ*G*^‡^_e = _23.5 kcal mol^−1^ at 308 K, consistent with the DFT barrier of 22.5 kcal mol^−1^, explaining its markedly slower racemization. We previously reported calculated racemization barriers for methyl-substituted DBP[6]CPP and DBP[7]CPP of 36.5 kcal mol^−1^ and 26.9 kcal mol^−1^, respectively. These smaller nanohoops exhibit higher barriers and slower racemization.^18^

### Supramolecular host properties of diketo[*n*]CPPs

With diameters between 13.5 Å and 16.5 Å, both diketo[8]- and diketo[9]CPPs as well as DBP[8]- and DBP[9]CPPs have cavities suitable to host C_60_ and C_70_ fullerenes. The host–guest complexes formed between the nanohoops and both C_60_ and C_70_ were characterized by high-resolution mass spectrometry (HRMS), NMR and fluorescence spectroscopy. ^1^H NMR spectra of diketo[*n*]CPPs and DBP[*n*]CPPs in TCE-*d*_2_ showed clear shifts of the proton resonances upon addition of excess C_60_ or C_70_ (see SI Section 7.2), except for the addition of C_60_ to DBP[9]CPP. This might be due to the large size of this nanohoop resulting in a reduced interaction with the C_60_ fullerene. All signals in the complexes show chemical shift changes or splitting, which can be rationalized by their different spatial locations relative to the shielding and deshielding regions created by the fullerene guests. HRMS analyses indicated ionized species with *m*/*z* values matching diketo[*n*]CPPs⊃C_60_ or DBP[*n*]CPPs⊃C_70_ for all nanohoops, demonstrating the possibility of host–guest-complex formation, also for DBP[9]CPP (see SI Section 7.1).

**Fig. 4:**
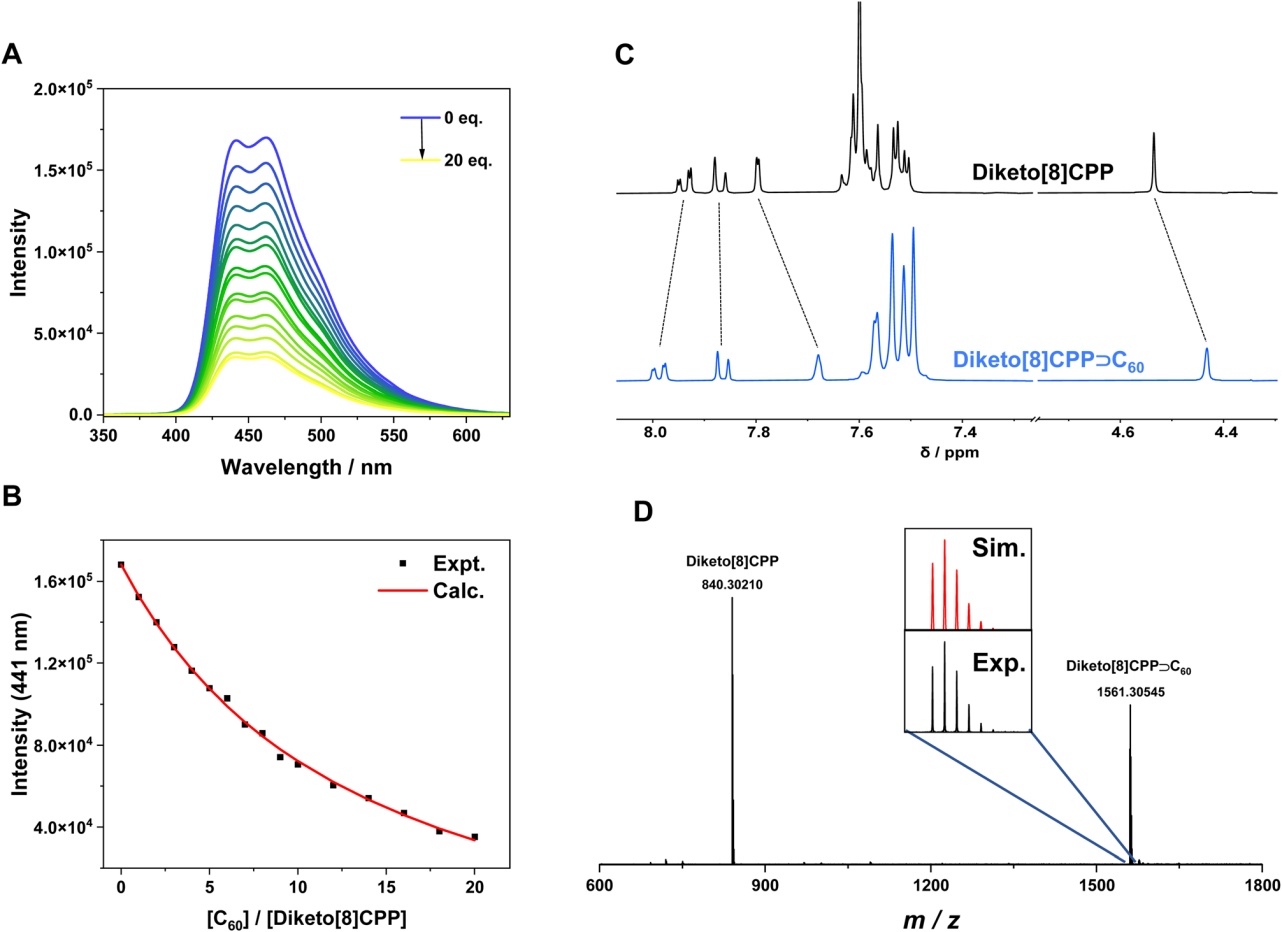
Supramolecular host properties of diketo[*n*]CPPs. (A and B) Fluorescence titration of diketo[8]CPP (1.2 × 10^−6^ M) with C_60_ in toluene (fit based on a 1 : 1 model); (C) ^1^H NMR spectra (1,1,2,2-tetrachloroethane-*d*_2_, 400 MHz) of diketo[8]CPP and its mixture with 3 eq. C_60_; (D) MALDI mass spectrum of diketo[8]CPP⊃C_60_ (inset shows the experimental and calculated isotopic pattern).

We quantified the binding of diketo[*n*]CPPs to fullerenes by conducting fluorescence titration experiments in toluene solution. The fluorescence of the diketo[*n*]CPPs is significantly quenched upon addition of C_60_ or C_70_ (Fig. 4A and B and SI Section 7.3), suggesting that a charge transfer may occur between the host and guest, similar to [*n*]CPPs.^36^ This was supported by the calculated frontier molecular orbitals of diketo[*n*]CPPs⊃C_60_ and diketo[*n*]CPPs⊃C_70_, where the HOMO is localized on the diketo[*n*]CPP and the LUMO on the fullerene (SI, Fig. S69). By fitting the titration curves using the BindFit program,^37–40^ we obtained the binding constants (*K*) for the host–guest complexes in toluene at room temperature. The average association constants for diketo[8]CPP⊃C_60_, diketo[8]CPP⊃C_70_, diketo[9]CPP⊃C_60_ and diketo[9]CPP⊃C_70_ are 6.3 × 10^4^, 7.2 × 10^4^, 5.2 × 10^4^, and 5.3 × 10^4^ M^−1^, respectively, in toluene (Table 2). These are lower than the binding constant for the C_60_ complex of [10]CPP in toluene of 2.8 × 10^6^ M^−1^,^36^ determined by fluorescence quenching titration, but similar to that for the C_60_ complex of [4]cyclodibenzopentalene of 5.3 × 10^4^ M^−1^ in toluene (from UV/vis-spectroscopic titration).^41^ The binding constants of the diketo[*n*]CPPs lie much closer to that of [10]CPP with C_70_ fullerene of 8.4 × 10^4^ M^−1^ in toluene.^8^ This is a very interesting observation: while [10]CPP binds C_60_ more than 30 times stronger than C_70_ in solution, for each diketo[*n*]CPP we obtained similar binding constants for both fullerenes (*K*(C_60_)/*K*(C_70_) ≈ 1). We reason that this observation is related to the shape adaptivity of the diketo[*n*]CPPs, maximizing their interacting surface with the fullerene guest in each case.

**Table 2:** Experimental binding constants^a^*K* of 1 : 1 host–guest complexes and free binding energies Δ*G* (298 K) from fluorescence titrations of diketo[*n*]CPPs with C_60_ and C_70_ in toluene

Experiment		*K* a/M^−1^	Δ*G*^b^/kcal mol^−1^
Diketo[8]CPP	C_60_	6.27(±0.02) × 10^4^	−6.55
	C_70_	7.15(±0.04) × 10^4^	−6.62
Diketo[9]CPP	C_60_	5.20(±0.02) × 10^4^	−6.43
	C_70_	5.33(±0.01) × 10^4^	−6.45

a Average of three titrations.

b Calculated using Δ*G* = −*RT* ln *K*.

**Fig. 5:**
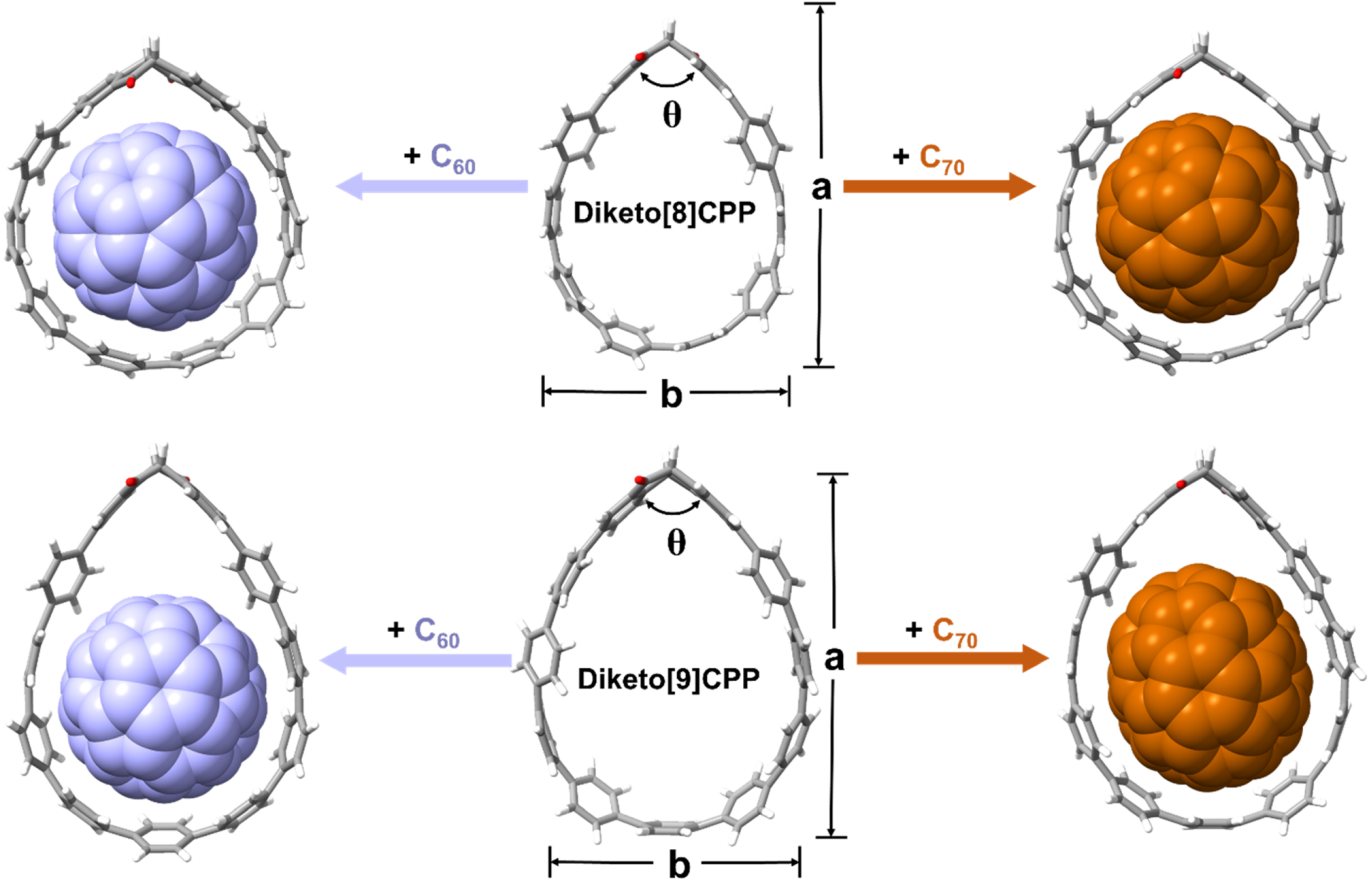
DFT-optimized geometries (B3LYP-D3(BJ)/6-31G(d)) and association free energies (GFN2-*x*TB) of diketo[*n*]CPPs and their fullerene complexes.

To further assess the non-covalent interactions and shape adaptivity of the diketo[*n*]CPPs towards fullerene binding, we optimized their molecular geometries as well as those of the fullerene complexes at the B3LYP-D3(BJ)/6-31G* level of theory (Fig. 5 and Table 3). C_60_ is approximately spherical with a mean diameter of 7.1 Å and fits well into both diketo[8]- and -[9]CPPs. It preferably interacts with the π-surfaces of the PP parts, especially visible in diketo[9]CPP⊃C_60_, where it is centered at the “bottom” part of the nanohoop as shown in Fig. 5. The nanohoops adapt their shape to best host C_60_: while the “kink” angle *θ* of the diketo unit in diketo[8]CPP increases from 110.7° to 114.9° in the fullerene complex, this angle gets smaller in diketo[9]CPP (111.6° to 108.3°) to maximize interactions of the π-surfaces. Diketo[8]CPP overall assumes a more circular shape (*a* and *b* values approaching each other) to host C_60_.

**Table 3:** Geometrical parameters of diketo[*n*]CPPs and their fullerene complexes, including long axis (*a*), short axis (*b*), and bending angle (*θ*)

	*a*/Å	*b*/Å	*θ*/°
Diketo[8]CPP	16.66	12.29	110.7
Diketo[8]CPP ⊃ C_60_	15.31	13.83	114.9
Diketo[8]CPP ⊃ C_70_	15.31	13.84	114.3
Diketo[9]CPP	18.22	13.43	111.6
Diketo[9]CPP ⊃ C_60_	18.30	13.48	108.3
Diketo[9]CPP ⊃ C_70_	18.0	13.96	110.2

Because of the anisotropic shape of fullerene C_70_, reminiscent of an American football with a short axis of 7.12 Å and a long axis of 7.96 Å, the supramolecular complexes formed between nanohoops and C_70_ are diverse.^8,42^ Based on the calculated association energies, C_70_ adopts a standing orientation in diketo[8]CPP, but a lying orientation in diketo[9]CPP (Fig. 5 and Table S11). Similar to the C_60_ complex, diketo[8]CPP has to widen its “kink” angle *θ* to 114.3° to best host C_70_, while for diketo[9]CPP accommodating C_70_ in standing orientation hardly requires any geometrical change of the nanohoop (*θ* = 110.2°). As for C_60_, diketo[8]CPP assumes a more circular shape in the C_70_ complex.

For each of the fullerene complexes, an independent gradient model based on Hirshfeld partition (IGMH) analysis^43,44^ showed that the *para*-phenylene moieties around the fullerene guest in each cavity contribute to the non-covalent interactions (SI, Fig. S70).

The calculated association-free energies^45^ for diketo[8]CPP⊃C_60_, diketo[8]CPP⊃C_70_, diketo[9]CPP⊃C_60_ and diketo[9]CPP⊃C_70_ at the GFN2-*x*TB level of theory in toluene amount to −5.5, −7.6, −4.7 and −5.6 kcal mol^−1^, respectively (see SI Table S13). This compares well to the experimental results in toluene (Table 2).

**Fig. 6:**
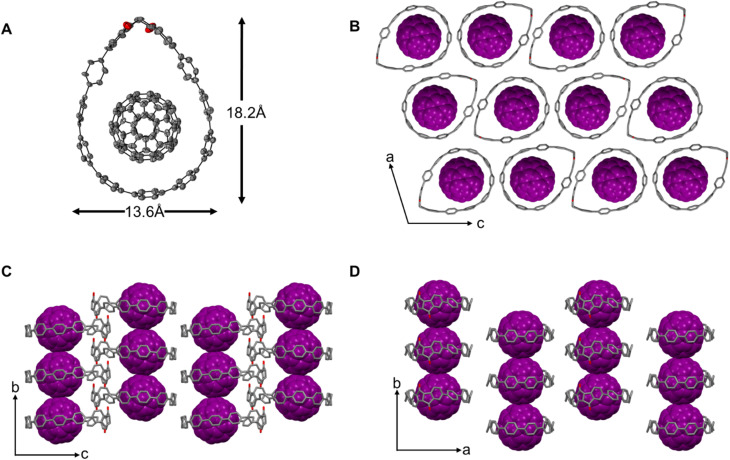
Single-crystal X-ray structural analysis of diketo[9]CPP ⊃ C_60_. (A) Top view of the solid-state structure of diketo[9]CPP ⊃ C_60_. (B–D) Packing structure. Solvent molecules and hydrogen atoms are omitted for clarity. Thermal ellipsoids are shown at the 50% probability level.

Single crystals of the complex diketo[9]CPP⊃C_60_ suitable for X-ray diffraction were obtained by vapor diffusion of MeCN into a 1,2-dichlorobenzene solution. The solid-state structure clearly shows that a complex between diketo[9]CPP and C_60_ with 1 : 1 stoichiometry is present. The fullerene inside the cavity was restrained and placed in two positions (see SI Section 8.2). The top half of the structure is disordered over two positions consisting of both enantiomers of the diketo[9]CPP structure with a ratio of 57/43. In the solid state, incorporation of C_60_ transforms the herringbone packing of diketo[9]CPP (Fig. 2E and F) into a columnar structure. Notably, the overall size of the diketo[9]CPP cavity shows only a slight variation upon complexation (Fig. 6).

The above-discussed solution-based complexation experiments were complemented by gas-phase experiments on the formation and relative stability of noncovalent complexes of diketo[9]- and DBP[9]CPPs with C_60_ and C_70_, respectively. For this, electrospray ionization-(tandem) mass spectrometry (ESI-(MS/)MS) was applied. ESI is a soft ionization method allowing the gentle transfer of loosely bound complexes from solution into the gas phase. In the present case, the formation of ions occurs by electrochemical oxidation with the ESI source operating as an electrochemical cell. Consequently, radical cations are formed through the oxidation of the molecules that constitute the noncovalent complex. The resulting ionized complexes are subjected to energy-resolved collision-induced dissociation (CID). Recording the decay as a function of the applied collision energy allows establishing the relative bond strength holding together the complex ion. The *E*_50_ value refers to the collision energy, at which half of the complex has decomposed, and is taken as a measure of stability. All oxidized nanohoop–fullerene complexes decomposed into the nanohoop radical cation by loss of a neutral fullerene. Evidently, within the complex, the nanohoop was oxidized in favour of the fullerene.

Table 4 compares the *E*_50_ values as a measure of the relative stabilities of the cationic host–guest complexes combining [*n*]CPP (*n* = 10, 11) as well as diketo[9]- and DBP[9]CPPs as the hosts with C_60_ and C_70_ as guests. [10]CPP˙^+^⊃C_60_ shows the strongest noncovalent bonding amongst the C_60_ complexes.^36,46^ [11]CPP˙^+^⊃C_60_ is slightly less strongly connected. The C_60_ complexes of diketo[9]- and DBP[9]CPP˙^+^ show only a slightly lower stability as [11]CPP˙^+^⊃C_60_, with the DBP[9]CPP complexes being marginally more stable than the diketo[9]CPP complexes. The stability of all host–guest complexes increases when C_70_ is incorporated. Even though [10]CPP has a considerably larger binding constant with C_60_ than with C_70_ in equilibrium measurements in solution,^8,36^ the dissociation of the isolated complex ions in the gas phase reveals a stronger bonding to C_70_. The latter has been observed before and attributed to enhanced interactions due to the larger π system of C_70_.^46^ [11]CPP˙^+^⊃C_70_ is the most stable C_70_ complex followed by [10]CPP˙^+^⊃C_70_. Also diketo[9]CPP, whose shape-adaptive binding led – in solution – to similar binding constants as C_60_ and C_70_, forms more stable isolated complex ions with C_70_ than with C_60_. Diketo[9]CPP˙^+^⊃C_70_ is slightly more stable than DBP[9]CPP˙^+^⊃C_70_. Evidently, the host–guest complexes employing the shape-adaptive diketo[9]CPP gain similar stabilities as those using DBP[9]CPP and [11]CPP as the hosts.

**Table 4:** *E*_50_ values of the investigated noncovalent radical cation complexes (host ⊃ guest^˥^ ˙^+^) in eV

Guest	Host			
	[10]CPP	[11]CPP	Diketo[9]CPP	DBP[9]CPP
C_60_	0.146	0.117	0.099	0.103
C_70_	0.158	0.172	0.141	0.115

## Conclusions and outlook

In summary, we presented diketo[*n*]CPPs and DBP[*n*]CPPs with *n* = 8 and 9 and studied their structures, photophysical properties, and interactions with fullerenes. All four nanohoops are chiral, with diketo[*n*]CPPs based on the central chirality of the diketo unit, and DBP[*n*]CPPs based on the hindered rotation of the DBP panels. Remarkably, diketo[*n*]CPPs showed high fluorescence quantum yields of 87% (*n* = 8) and 92% (*n* = 9), while the fluorescence in DBP[*n*]CPPs was quenched, likely due to energy transfer to the non-emissive DBP units. Diketo[8]CPP and diketo[9]CPPs deliver valuable CPL brightness values (B_CPL_) of 32.5 and 41.2 M^−1^ cm^−1^, respectively, while the DBP[*n*]CPPs racemize easily owing to their lower enantiomerization barriers. The diketo unit bestows diketo[*n*]CPPs with a shape-adaptive structure, where through the change of its kink angle the geometrical parameters of the nanohoops can adapt. This unique property enabled diketo[8]- and diketo[9]CPPs to form stable complexes with both C_60_ and C_70_, showing similar binding strengths in the range of 5 × 10^4^ to 7 × 10^4^ M^−1^ in toluene to both fullerene guests due to their shape adaptivity. This is supported by DFT calculations on the structures of the inclusion complexes, clearly demonstrating the changes in the nanohoop shape. Collision experiments in the gas phase reveal that noncovalent complex ions with C_60_ and C_70_ possess similar individual stabilities using either diketo[9]CPP or DBP[9]CPP as the host with bond strengths similar to the use of [11]CPP as the host. Having uncovered the basic structural and optoelectronic properties of diketo[*n*]CPPs and DBP[*n*]CPPs, we are now in a position to fully utilize these unique nanohoops for various applications.

## Author contributions

Conceptualization: B. E. and X. X.; data curation: all authors; formal analysis: X. X., M. H., L. Y., P. S., and L. E.; funding acquisition: B. E.; investigation: X. X., M. H., L. Y., P. S., and L. E.; methodology: X. X., M. H., L. Y., P. S., and L. E.; project administration: B. E.; supervision: B. E., G. P., and T. D.; validation: all authors; visualization: X. X.; writing – original draft: X. X. and B. E.; writing – review & editing: all authors.

## Conflicts of interest

There are no conflicts to declare.

## Supplementary Material

SC-017-D5SC05305F-s001

SC-017-D5SC05305F-s002

## Data Availability

The data that support the findings of this study are openly available in Zenodo at https://zenodo.org/doi/10.5281/zenodo.15847822, reference number 15847822. CCDC 2364437, 2365575, 2427456 and 2486379 contain the supplementary crystallographic data for this paper.^47*a*–*d*^ Supplementary information: materials and methods, synthetic procedures and characterization data, NMR and mass spectra, further spectra from UV/Vis, fluorescence, CD and CPL measurements, details on host-guest studies, SC-XRD data, and details on DFT calculations. See DOI: https://doi.org/10.1039/d5sc05305f.
