# Supplementary material for: Diketo[n]CPPs as chiral and shape-adaptive fullerene hosts and precursors to DBP[n]CPPs
Source: Chem Sci. 2026 Jan 26;17(13):6677–87. doi: 10.1039/d5sc05305f (PMC12890307; doi:10.1039/d5sc05305f)
Supplement: SC-017-D5SC05305F-s001 [file SC-017-D5SC05305F-s001.pdf]

Supplementary Information (SI) for Chemical Science.
This journal is © The Royal Society of Chemistry 2026

Supporting Information

Diketo[*n*]CPPs as Chiral and Shape-adaptive Fullerene Hosts and Precursors to DBP[*n*]CPPs

Xiaoshuang Xiang,[a] Mathias Hermann,[a] Lei Ye,[b] Philipp Seitz,[a] Lilian Estaque,[c] Grégory Pieters,[c] Thomas Drewello[b] and Birgit Esser*[a]

Affiliations:

a. Institute of Organic Chemistry II and Advanced Materials, Ulm University, Albert-Einstein-Allee 11, 89081 Ulm, Germany; birgit.esser@uni-ulm.de
b. Physical Chemistry I, Department of Chemistry and Pharmacy, Friedrich-Alexander-Universität Erlangen-Nürnberg, Egerlandstraße 3, 91058 Erlangen, Germany.
c. Université Paris-Saclay, CEA, Département Médicaments et Technologies pour la Santé (DMTS), SCBM, 91191 Gif-sur-Yvette, France

Contents

1. Materials and Methods S2

2. Synthetic Manipulations S5

3. NMR Spectra S14

4. Mass Spectra S28

5. Optical Properties S30

5.1 Determination of Molar Extinction Coefficients S30

5.2 Absorption and Emission Spectra S32

6. Chiral Resolution, Circular Dichroism and Circularly Polarized Luminescence Spectra S33

6.1 Chiral resolution and the chiroptical properties for diketo[n]CPP S33

6.2 Dynamic HPLC elution profiles and the thermodynamic analysis for DBP[n]CPP S34

7 Supramolecular Chemistry S41

7.1 MS Spectra of Nanohoop-Fullerene Complexes S41

7.2 NMR Studies of Nanohoop-Fullerene Complexation S48

7.3 Fluorescence Titration Experiments S52

8. Single Crystal X-Ray Diffraction S56

8.1 General Experimental Setup and Solution S56

8.2 Details for Each Crystal S57

9. DFT Calculations S62

9.1 Strain Energies S62

9.2 Calculation of Racemization Barriers S65

9.3 TD-DFT Calculations S66

9.4 Calculations of Fullerene Complexes S73

10 References S78

1. Materials and Methods

Chemicals and solvents

All reagents were obtained from ABCR, ACROS-ORGANICS, ALFA-AESAR, BLDPHARM, CHEMPUR, SIGMA-ALDRICH / MERCK or TCI. Moisture- or oxygen-sensitive reactions were carried out in dried glassware, heated under vacuum (< 10^{-2} mbar), using standard Schlenk techniques in a dry argon atmosphere (Argon 4.6 from MTI IndustrieGase AG). Anhydrous solvents (Toluene, THF) were obtained from an M. BRAUN solvent purification system (MB-SPS-800) and stored over activated molecular sieves (3 Å). Anhydrous 1,4-dioxane was purchased from ACROS-ORGANICS (extra dry, < 50 ppm H_2O) and further stored over activated molecular sieves (3 Å). Other solvents were purchased and used in analytical or HPLC grade. All the sensitive reactions were performed using standard vacuum-line and Schlenk techniques. Petroleum ether (technical grade, boiling range 40–65 °C) served as the main eluent.

Analytical thin layer chromatography

Thin layer chromatography was carried out using silica gel-coated aluminum plates with a fluorescence indicator (MERCK 60 F_{254}, purchased from Merck KGaA). Detection was carried out by using UV-light (λ_{max} = 254 and 366 nm).

Flash column chromatography

Flash column chromatography was carried out using silica gel 60, grain size 40–63 µm (230–400 mesh) from MACHERY-NAGEL.

Nuclear magnetic resonance spectroscopy (NMR)

NMR spectra were recorded at 300 K, unless otherwise stated, on the following spectrometers: BRUKER *Avance NEO 400* with BBFO probe [400 MHz (^{1}H), 100 MHz (^{13}C)], BRUKER *Avance NEO 600* with iProbe BBO Cryoprobe [600 MHz (^{1}H), 150 MHz (^{13}C)]. Chemical shifts are reported in parts per million (ppm, δ scale) relative to the signal of tetramethylsilane (δ = 0.00 ppm). ^{1}H NMR spectra are referenced to tetramethylsilane as an internal standard or the residual solvent signal of the respective solvent: $CDCl_3$: δ = 7.26 ppm; CD_2Cl_2: δ = 5.32 ppm, $(CD_3)CO$: δ = 2.05 ppm, 1,1,2,2-tetrachloroethane-d_2: δ = 5.91 ppm. ^{13}C NMR spectra are referenced to the following signals: $CDCl_3$: δ = 77.16 ppm, CD_2Cl_2: δ = 53.84 ppm, $(CD_3)CO$: δ = 28.84, 206.36

ppm,[1].Analysis followed first order, and the following abbreviations for multiplets were used: singlet (s), broad singlet (br. s), doublet (d), triplet (t), quartet (q), septet (sept), multiplet (m) and combinations thereof i.e. doublet of doublets (dd). Coupling constants (*J*) are given in Hertz [Hz].

High resolution mass spectrometry (HRMS)

HRMS spectra were measured on a THERMO FISHER SCIENTIFIC Exactive mass spectrometer with an orbitrap analyser with atmospheric-pressure ionization (APCI) as ionization methods. HRMS was measured on a BRUKER solariX Hybrid 7T FT-ICR via matrix assisted laser desorption/ionization (MALDI). The used matrix is trans-2-[3-(4-tert-butylphenyl)-2-methyl-2-propenylidene]malononitrile (DCTB). ESI mass spectra (MS^1) experiments were performed on a quadrupole time-of-flight mass spectrometer (microTOF-Q II, Bruker Daltonics, Bremen) equipped with an electrospray ionization (ESI) source. The analyte solutions were directly injected into the ESI source with a syringe pump at a flow rate of 180 μl h^{-1}. The temperature of the nitrogen heating gas was set to 180 °C and a capillary voltage of −4.5 kV was applied. Prior to each experiment, the instrument parameters were optimized to obtain good signal intensities. MS^2 (CID) experiments were carried out with N_2 as collision gas which was generated by a Parker LCMS64 nitrogen generator with a purity of 99.999% and a flow rate of 0.2 l min^{-1}.

UV/Vis absorption spectra

UV/Vis absorption spectra were measured on a SHIMADZU UV-3600i Plus using Quartz cuvettes (1 cm path length) from HELLMA ANALYTICS. Extinction coefficients were measured by serial dilution. Spectra were measured in 5 steps from 10^{-6} to 10^{-5} M. Extinction coefficient values were then averaged over all measurements.

Fluorescence spectra

Photoluminescence measurements were conducted with a PerkinElmer FL6500 instrument, using quartz cuvettes with a path length of 1.0 cm at 298 K.

Photoluminescence Quantum Yields

Photoluminescence quantum yields (PLQYs) were obtained from Hamamatsu Quantaurus-QY (C11347).

Gel permeation chromatography (GPC)

GPC was performed on LaboACE LC-7080 Plus with CH_2Cl_2 as the eluent.

High performance liquid chromatography (HPLC)

HPLC was performed on KNAUER AZURA Semipreparative HPLC system. Analytical HPLC analyses were performed on a KNAUER AZURA system equipped with a DAD 2.1L detector, ASM 2.2L autosampler, P 6.1L (10 mL) pump, VU 4.1 valve unit, AS 6.1L autosampler, and CT 2.1 column thermostat.

Electronic circular dichroism (ECD) and circularly polarized luminescence (CPL)

Electronic circular dichroism (ECD) were recorded in an Olis DSM 245 spectrophotometer equipped with a non-ozone producing xenon lamp of 150 W. Circularly polarized luminescence (CPL) measurements were carried out in dichloromethane (DCM) solution (5×10^{-5} M) at room temperature using a Jasco CPL-300 spectrometer and a 10 mm × 10 mm quartz cuvette. The acquisition parameters were as follows: a data pitch of 1 nm, a scanning speed of 100 nm/min, and an excitation wavelength of 330 nm. The spectra presented represent the average of at least 10 accumulations.

2. Synthetic Manipulations

1 and **5** were synthesized according to previously reported procedures.[2-4]

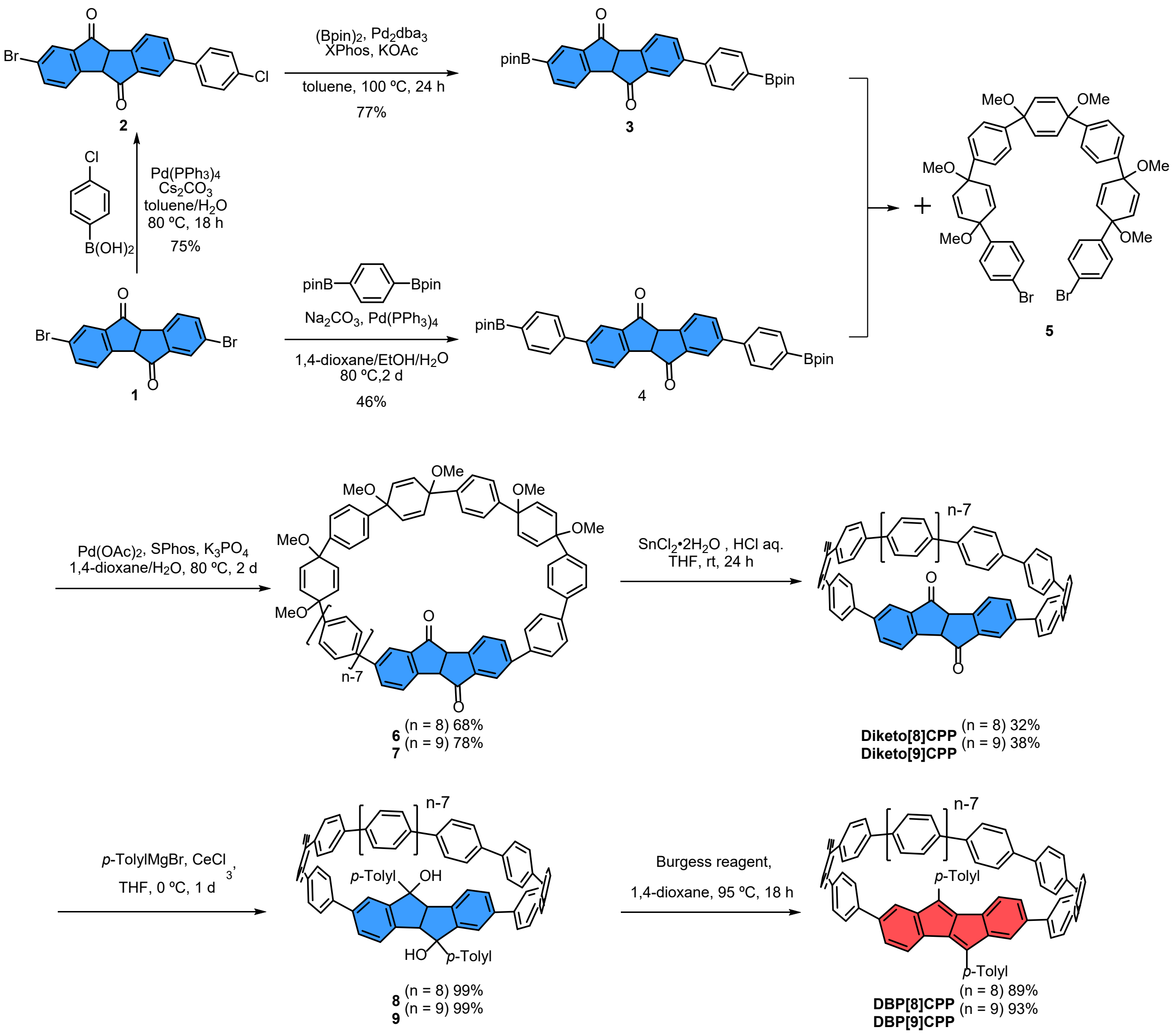

Scheme 1. Synthesis of Diketo[n]CPPs and DBP[n]CPPs (n = 8, 9).

2-Bromo-7-(4-chlorophenyl)-4b,9b-dihydroindeno[2,1-*a*]indene-5,10-dione (2)

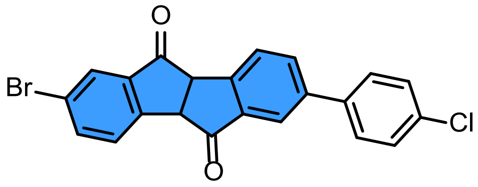

2

A solution of **1** (0.78 g, 2.0 mmol, 1 eq.), 4-chlorophenylboronic acid (315.1 mg, 2.2 mmol, 1.1 eq.), Cs_2CO_3 (1.3 g, 4.1 mmol, 2 eq.) and $Pd(PPh_3)_4$ (235.8 mg, 0.2 mmol, 0.1 eq.) in a degassed mixture of toluene (200 mL) and H_2O (20 mL) was stirred at 70 °C for 18 h. Toluene was removed under vacuum, and the aqueous layer was extracted with CH_2Cl_2(3 × 50 mL). The combined organic layer was purified by silica gel column chromatography (eluent: Ethyl acetate/ Petroleum ether = 1/6) in a long column to yield **2** as a white solid (0.63 g, 1.5 mmol, 75%).

***R*f** 0.45 (Ethyl acetate/ Petroleum ether: 1/5); ^{1}H NMR (400 MHz, $CDCl_3$): δ 8.00–7.93 (m, 1H), 7.90–7.77 (m, 5H), 7.51–7.46 (m, 2H), 7.44–7.38 (m, 2H), 4.47 (d, *J* = 5.7 Hz, 1H), 4.40 (d, *J* = 5.7 Hz, 1H); ^{13}C NMR (100 MHz, $CDCl_3$): δ 13C NMR (101 MHz, CDCl3) δ 200.8, 200.1, 148.6, 148.4, 141.7, 138.8, 138.0, 136.7, 135.6, 135.0, 134.5, 129.4, 128.5, 128.2, 127.9, 127.2, 123.8, 123.0, 52.7 (One carbon signal is overlapping at 52.68); **HRMS** (pos. APCI): *m*/*z* calcd for $C_{22}H_{13}{}^{79}BrClO_2$ 422.9782 $[M+H]^+$, found 422.9791.

2-(4,4,5,5-Tetramethyl-1,3,2-dioxaborolan-2-yl)-7-(4-(4,4,5,5-tetramethyl-1,3,2-dioxaborolan-2-yl)phenyl)-4b,9b-dihydroindeno[2,1-*a*]indene-5,10-dione (3)

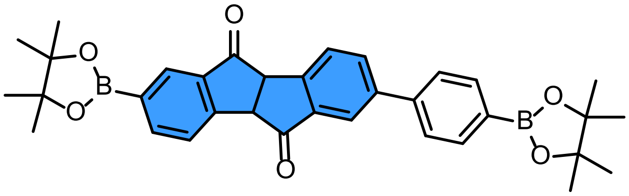

3

A mixture of **2** (435.9 mg, 1.0 mmol, 1 eq.), bis(pinacolato)diboron (1.57 g, 6.2 mmol, 6 eq.), KOAc (605.8 mg, 6.2 mmol, 6 eq.), $Pd_2(dba)_3$ (37.7 mg, 41.2 µmol, 4 mol%) and XPhos (39.2 mg, 82.4 µmol, 8 mol%) in anhydrous toluene (40 mL) was stirred at 100 °C for 1 d. The toluene was removed under vacuum, and the mixture was extracted with CH_2Cl_2(3 × 40 mL). The organic phase

was dried over Na_2SO_4. The crude product was purified using a short silica gel column (eluent: Ethyl acetate/ Petroleum ether: 1/4) to provide **3** as a white solid (0.43mg, 0.77 mmol, 77%).

***R*f** 0.40 (Ethyl acetate/ Petroleum ether: 1/4); ^{1}H NMR (400 MHz, $(CD_3)_2CO$): δ 8.10 (m, 2H), 8.04 – 7.81 (m, 6H), 7.75 – 7.69 (m, 2H), 4.65 (d, *J* = 5.8 Hz, 1H), 4.61 (d, *J* = 5.9 Hz, 1H), 1.35 (s, 12H), 1.33 (s, 12H); ^{13}C NMR (100 MHz, $(CD_3)_2CO$): δ 201.7, 201.5, 153.7, 150.3, 143.0, 142.8, 142.2, 136.6, 136.2, 135.5, 131.4, 128.0, 127.3, 127.0, 123.0, 85.1, 84.7, 54.2, 53.3, 25.2, 25.1(Three aromatic carbon signals are overlapping); **HRMS** (pos. APCI): m/z calcd for $C_{34}H_{37}B_2O_6^+$ 563.2771 $[M+H]^+$, found 563.2779.

2,7-Bis(4-(4,4,5,5-tetramethyl-1,3,2-dioxaborolan-2-yl)phenyl)-4b,9b-dihydroindeno[2,1-*a*]indene-5,10-dione (4)

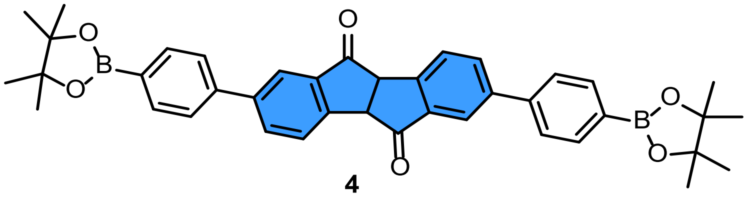

4

Compound **1** (1.94 g, 5.0 mmol, 1.0 eq.) and 1,4-bis(4,4,5,5-tetramethyl-1,3,2-dioxaborolan-2-yl)benzene (8.25 g, 25 mmol, 5.0 eq.) were dispersed in a mixture of 1,4-dioxane (60 mL), ethanol (30 mL), and deionized water (30 mL) in a 250 mL round-bottom flask. The mixture was then degassed by sparging with Ar for 30 minutes. $Pd(PPh_3)_4$ (115 mg, 0.1 mmol, 2.0 mol%) and Na_2CO_3 (5.25 g, 50.0 mmol, 10 eq.) were added to the mixture, which was then further purged with Ar for another 15 minutes. Thereafter, the mixture was stirred at 80 °C for 36 h, and then quenched by addition of methanol (10 mL). The mixture was extracted with CH_2Cl_2(3 × 40 mL). The organic phase were dried over Na_2SO_4, and the solvents were removed under reduced pressure. The crude product was purified by column chromatography (silica gel, Petroleum ether/ Ethyl acetate: 4/1) followed by recrystallization from CH_2Cl_2/ Ethyl acetate to afford **4** as white solid (1.5 g, 2.3 mmol, 46%).

***R*f** 0.45 (Petroleum ether/ Ethyl acetate: 4/1); ^{1}H NMR (400 MHz, $CDCl_3$): δ 8.03–7.92 (m, 6H), 7.89–7.86 (m, 4H), 7.60–7.54 (m, 4H), 4.50 (s, 2H), 1.35 (s, 24H); ^{13}C NMR (100 MHz, $CDCl_3$): δ 201.6, 149.0, 142.6, 142.2, 135.64, 135.59, 135.1, 127.0, 126.6, 123.2, 84.1, 52.9, 25.0. (One aromatic carbon signal is overlapping); **HRMS** (pos. MALDI): m/z calcd for $C_{40}H_{40}B_2O_6Na^+$ 661.2903 $[M+Na]^+$, found 661.2916.

Compound 6

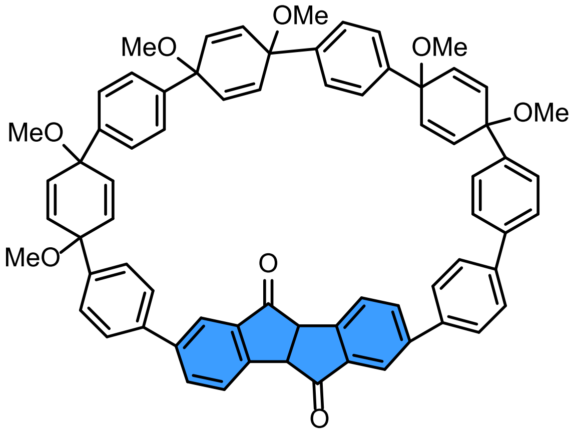

6

A solution of **3** (140.6 mg, 0.25 mmol, 1.0 eq.), C-shaped precursor **5** (219.7 mg, 0.25 mmol, 1.0 eq.) and K_3PO_4 (530.7 mg, 2.5 mmol, 10 eq.) in 1,4-dioxane (150 mL) and H_2O (10 mL) was degassed by sparging with Ar for 2 h. Then, $Pd(OAc)_2$ (28.1 mg, 0.125 mmol, 0.5 eq.) and SPhos (102.6 mg, 0.25 mmol, 1 eq.) were added under an argon atmosphere. The reaction flask was sealed and stirred at 80 °C for 2 d. The reaction mixture was filtered over a pad of Celite® (washed with mixture of CH_2Cl_2 and EtOAc), the solvent was removed under reduced pressure, and the mixture was extracted with CH_2Cl_2 (3 × 50 mL). The combined organic extracts were dried over Na_2SO_4. The crude product was purified using a short silica gel column (eluent: Petroleum ether/ Ethyl acetate: 1/2) to yield **6** as a light yellow solid (0.17 g, 0.17 mmol, 68%), which was used directly in the next step without further purification. (Note: Trace amounts of acyclic oligomers were observed. These compounds did not interfere with isolation of the target macrocycles.)

$\mathbf{R_f}$ 0.40 (Petroleum ether/ Ethyl acetate: 1/2); **HRMS** (pos. MALDI): m/z calcd for $C_{70}H_{58}O_8$ 1026.4126$^+$ $[M]^{\bullet+}$, found 1026.4195.

Compound 7

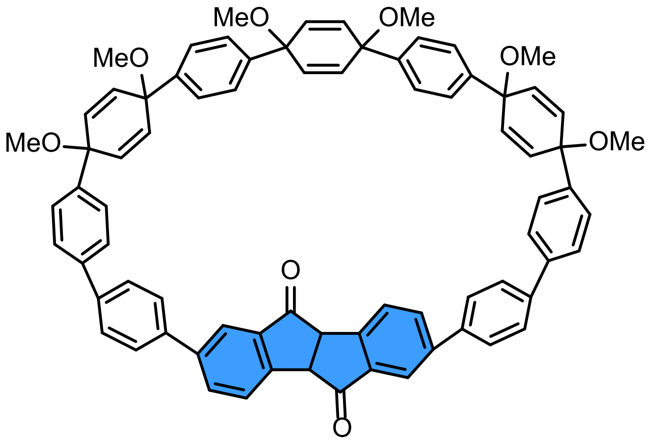

7

7 was synthesized using the same procedure described above for compound **6** using **4** (319 mg, 0.5 mmol, 1.0 eq.), C-shaped precursor **5** (438 mg, 0.5 mmol, 1.0 eq.), $Pd(OAc)_2$ (58 mg, 0.25 mmol, 0.5 eq.), SPhos (205 mg, 0.5 mmol, 1.0 eq.), K_3PO_4 (1.06 g, 5 mmol, 10 eq.), 1,4-dioxane (300 mL) and H_2O (20 mL). **7** was obtained as a white solid (0.43 g, 0.39 mmol, 78%).

***R*f** 0.40 (Petroleum ether/ Ethyl acetate: 1/2); **HRMS** (pos. MALDI): m/z calcd for $C_{76}H_{62}O_8^+$ 1102.4439 $[M]^{\bullet+}$, found 1102.4434.

Diketo[8]CPP

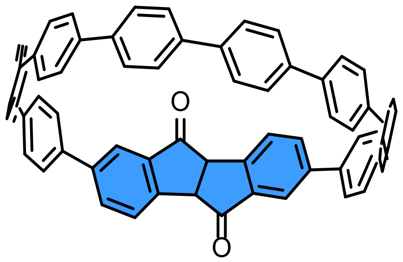

Diketo[8]CPP

$SnCl_2{\cdot}2H_2O$ (393 mg, 1.74 mmol, 13.0 eq.) was dissolved in degassed THF (30 mL). Concentrated aq. HCl (37% w/w, 12 M, 0.22 mL, 2.68 mmol, 20.0 eq.) was added dropwise, and the resulting solution was stirred at room temperature for 30 min. In a separate flask **6** (137.7 mg, 134 µmol, 1.00 eq.) was dissolved in degassed THF (10 mL), and the resulting solution was added dropwise to the H_2SnCl_4 solution. The resulting fluorescent solution was stirred at room temperature for 24 h. The reaction was quenched by addition of aq. KOH (2 M, 400 mL). The mixture was extracted with ethyl acetate (3 × 50 mL). The solvent was removed under reduced pressure. The residue was

firstly purified by passing through a short silica gel column (CH_2Cl_2) and then by preparative GPC with CH_2Cl_2 as eluent. Diketo[8]CPP was afforded as a yellow solid (36 mg, 43 µmol, 32%).

***R*f** 0.55 (CH_2Cl_2/ Petroleum ether: 2/1); **^{1}H NMR** (400 MHz, $CDCl_3$): δ 7.93–7.83 (m, 4H), 7.80 (dd, *J* = 1.8, 0.7 Hz, 2H), 7.64–7.48 (m, 32H), 4.52 (s, 2H); **^{13}C NMR** (100 MHz, $CDCl_3$): δ 202.3, 148.8, 141.7, 140.2, 138.73, 138.65, 138.3, 138.2, 137.8, 137.2, 134.6, 134.1, 127.8, 127.74, 127.72, 127.66, 127.6, 127.4, 127.29, 127.27, 127.0, 122.8, 52.9 (One aromatic carbon signal is overlapping); **HRMS** (pos. MALDI): m/z calcd for $C_{64}H_{40}O_2^+$ 840.3023 $[M]^{•+}$, found 840.3021.

Diketo[9]CPP

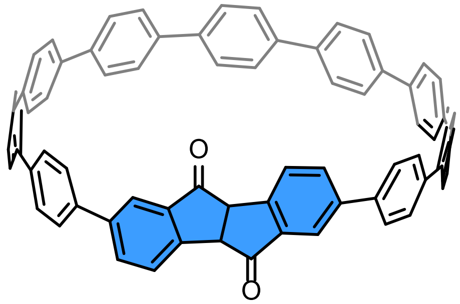

Diketo[9]CPP

Diketo[9]CPP was synthesized using the same procedure described above for Diketo[8]CPP using **7** (551 mg, 0.5 mmol, 1.0 eq.), $SnCl_2{\cdot}2H_2O$ (1.68 g, 7.5 mmol, 13.0 eq.), concentrated aq. HCl (37% w/w, 12 M, 0.83 mL, 10 mmol, 20.0 eq.) and THF (50+10 mL). Diketo[9]CPP was obtained as a yellow solid (0.17 g, 0.19 mmol, 38%).

***R*f** 0.5 (CH_2Cl_2/ Petroleum ether: 2/1); **^{1}H NMR** (400 MHz, $CDCl_3$): δ 7.99–7.85 (m, 6H), 7.73–7.50 (m, 36H), 4.54 (s, 2H); **^{13}C NMR** (150 MHz, $CDCl_3$): δ 202.28, 202.3, 148.8, 141.7, 140.2, 139.0, 138.98, 138.8, 138.5, 138.4, 138.22, 138.16, 137.4, 134.6, 134.2, 127.8, 127.7, 127.64, 127.59, 127.5, 127.33, 127.30, 127.28, 127.0, 122.8, 52.9 (One aromatic carbon signal is overlapping); **HRMS** (pos. MALDI): m/z calcd for $C_{70}H_{44}O_2^+$ 916.3336 $[M]^{•+}$, found 916.3337.

Compound 8

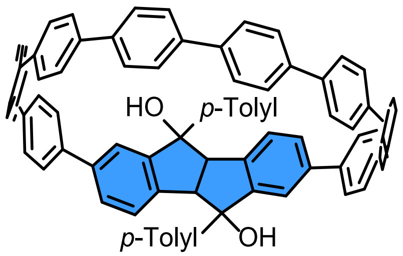

8

In a Schlenk tube anhydrous $CeCl_3$ (234 mg, 1.07 mmol, 20.00 eq.) was dried at 150 °C *in vacuo* (10^{-3} mbar) for 5 h. After cooling to room temperature and introducing argon, anhydrous THF (6 mL) was added. The mixture was stirred at room temperature for 18 h. In a separate Schlenk tube Diketo[8]CPP (45 mg, 54 µmol, 1.00 eq.) was dissolved in anhydrous THF (4 mL), added to the reaction mixture and then stirred at room temperature for 4.5 h. A solution of *p*-tolyl magnesium bromide in THF (1 M, 0.80 mL, 0.80 mmol, 15 eq.) was added to the $CeCl_3$ suspension at 0 °C. The resulting mixture was stirred at 0 °C for 1 h. The solution was allowed to warm to room temperature and was stirred for another 2 h. Saturated aq. NaCl was added, and the mixture was extracted with CH_2Cl_2 (3 × 30 mL). The combined organic extracts were washed with brine and dried over Na_2SO_4. The solvent was removed under reduced pressure, and the crude product was purified by column chromatography (silica gel, Petroleum ether / Ethyl acetate: 3/1). The title compound **8** (55 mg, 53 µmol, 99%) was obtained as a yellow solid.

***R*f** 0.6 (CH_2Cl_2/ Petroleum ether: 2/1); **^{1}H NMR** (400 MHz, $CDCl_3$): δ 7.86 (d, *J* = 8.0 Hz, 2H), 7.70–7.47 (m, 36H), 7.20 (d, *J* = 8.0 Hz, 4H), 7.12 (d, *J* = 8.0 Hz, 4H), 4.22 (s, 2H), 2.46 (s, 2H), 2.32 (s, 6H); **^{13}C NMR** (100 MHz, $CDCl_3$): δ 148.6, 144.7, 140.2, 139.5, 138.9, 138.7, 138.5, 138.31, 138.29, 138.2, 137.8, 137.1, 129.2, 128.1, 127.74, 127.68, 127.66, 127.6, 127.4, 127.3, 127.2, 127.1, 125.3, 121.9, 85.8, 59.2, 21.1(Two aromatic carbon signals are overlapping); **HRMS** (pos. MALDI): m/z calcd for $C_{78}H_{56}O_2^{+}$ 1024.42750 $[M]^{\bullet+}$, found 1024.4273.

Compound 9

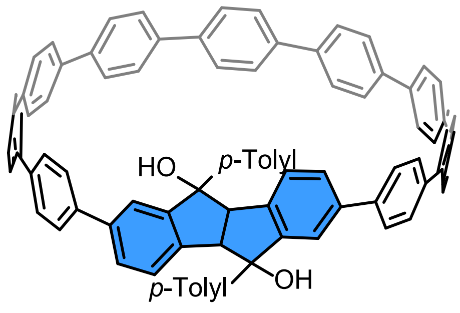

9

9 was synthesized using the same procedure described above for compound **8** using Diketo[9]CPP (40 mg, 43 µmol, 1.0 eq.), $CeCl_3$ (236 mg, 0.96 mmol, 20.00 eq.), *p*-tolyl magnesium bromide in THF (1 M, 0.64 mL, 0.64 mmol, 15 eq.)and THF (6 + 4 mL). **9** was obtained as a white solid (47 mg, 42 µmol, 99%).

***R*f** 0.6 (CH_2Cl_2/ Petroleum ether: 2/1); **^{1}H NMR** (400 MHz, $CDCl_3$): δ 7.86 (d, *J* = 8.0 Hz, 2H), 7.70–7.46 (m, 40H), 7.18 (d, *J* = 8.0 Hz, 4H), 7.11 (d, *J* = 8.0 Hz, 4H), 4.21 (s, 2H), 2.47 (s, 2H), 2.31 (s, 6H); **^{13}C NMR** (100 MHz, CD_2Cl_2): δ 149.1, 145.2, 140.4, 139.5, 139.23, 139.17, 139.1, 139.0, 138.9, 138.7, 138.48, 138.46, 137.4, 129.3, 128.8, 127.9, 127.83, 127.77, 127.74, 127.71, 127.5, 127.41, 127.38, 125.6, 121.9, 85.9, 59.5, 21.1. (Three aromatic carbon signals are overlapping); **HRMS** (pos. MALDI): m/z calc. for $C_{84}H_{60}O_2^+$ 1100.4588 $[M]^{\bullet+}$, found 1100.4566.

DBP[8]CPP

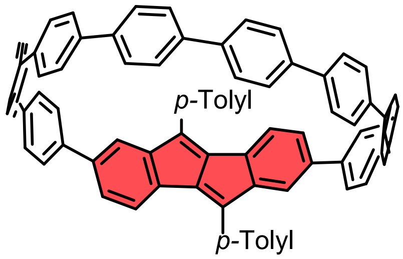

DBP[8]CPP

A flame-dried flask was charged with **8** (54 mg, 53 µmol, 1.00 eq.) and Burgess's reagent (151.4 mg, 636 µmol, 12.0 eq.). The flask was evacuated and backfilled with argon, and anhydrous 1,4-dioxane (10 mL) was added. The reaction mixture was stirred at 95 °C in a sealed flask for 20 h. After cooling to room temperature, the reaction was quenched by addition of saturated aqueous $NaHCO_3$ (30 mL). The aqueous mixture was extracted with CH_2Cl_2 (3 × 50 mL). The organic

extracts were dried over Na_2SO_4, filtered, and the solvent was removed under reduced pressure. Column chromatography (silica gel, Petroleum ether /CH_2Cl_2: 2/1) afforded the title compound (47 mg, 47 µmol, 89%) as a black solid.

***R*f** 0.4 (Petroleum ether /CH_2Cl_2: 2/1); **^{1}H NMR** (400 MHz, $CDCl_3$): δ 7.62–7.43 (m, 36H), 7.29 (m (badly resolved AA'BB' signal), 4H), 7.20 (m, 2H), 7.10 (m, 4H), 2.45 (s, 6H); **^{13}C NMR** (150 MHz, $CDCl_3$): δ 150.7, 142.8, 140.8, 139.8, 139.0, 138.9, 138.7, 138.63, 138.60, 138.5, 138.4, 134.0, 131.2, 129.5, 128.5, 127.63, 127.56, 127.53, 127.49, 127.47, 127.41, 127.36, 127.0, 125.7, 122.5, 121.1, 21.7 (Four aromatic carbon signals are overlapping); **HRMS** (pos. MALDI): m/z calcd for $C_{78}H_{52}^+$ 998.4064 $[M]^{•+}$, found 998.4059.

DBP[9]CPP

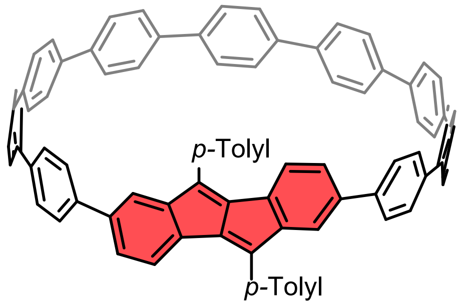

DBP[9]CPP

DBP[9]CPP was synthesized using the same procedure described above for compound DBP[8]CPP using **9** (46 mg, 42 µmol, 1.0 eq.), Burgess's reagent (119.9 mg, 504 µmol, 12.0 eq.) and anhydrous 1,4-dioxane (10 mL). DBP[9]CPP was obtained as a black solid (41 mg, 39 µmol, 93%).

***R*f** 0.4 (Petroleum ether /CH_2Cl_2: 2/1); **^{1}H NMR** (400 MHz, $CDCl_3$) δ 7.65–7.54 (m, 28H), 7.49 (m, 12H), 7.31 (m(badly resolved AA'BB' signal), 4H), 7.22 (d, *J* = 1.5 Hz, 2H), 7.14 (d, *J* =7.9 Hz, 2H), 7.10 (dd, *J* = 7.9, 1.5 Hz, 2H), 2.45 (s, 6H); **^{13}C NMR** (151 MHz, $CDCl_3$): δ 150.6, 142.6, 140.8, 139.6, 138.8, 138.6, 138.5, 138.4, 138.32, 138.30, 138.2, 138.0, 133.7, 131.1, 129.4, 128.4, 127.54, 127.50, 127.46, 127.43, 127.40, 127.35, 127.29, 127.22, 126.8, 125.6, 122.4, 120.9, 21.5 (Four aromatic carbon signals are overlapping); **HRMS** (pos. MALDI): m/z calcd for $C_{84}H_{56}^+$ 1064.4377 $[M]^{•+}$, found 1064.4376.

3. NMR Spectra

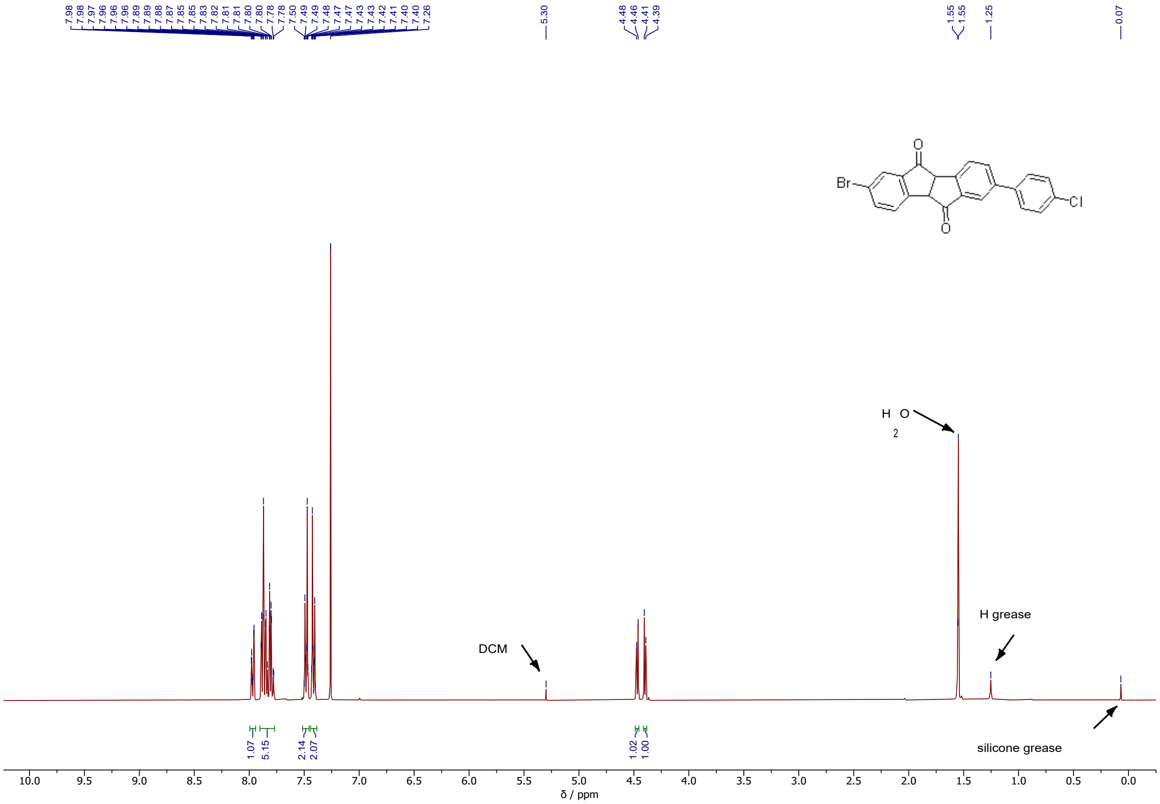

Figure S1.^{1}H NMR spectrum (400 MHz, $CDCl_3$) of **2**.

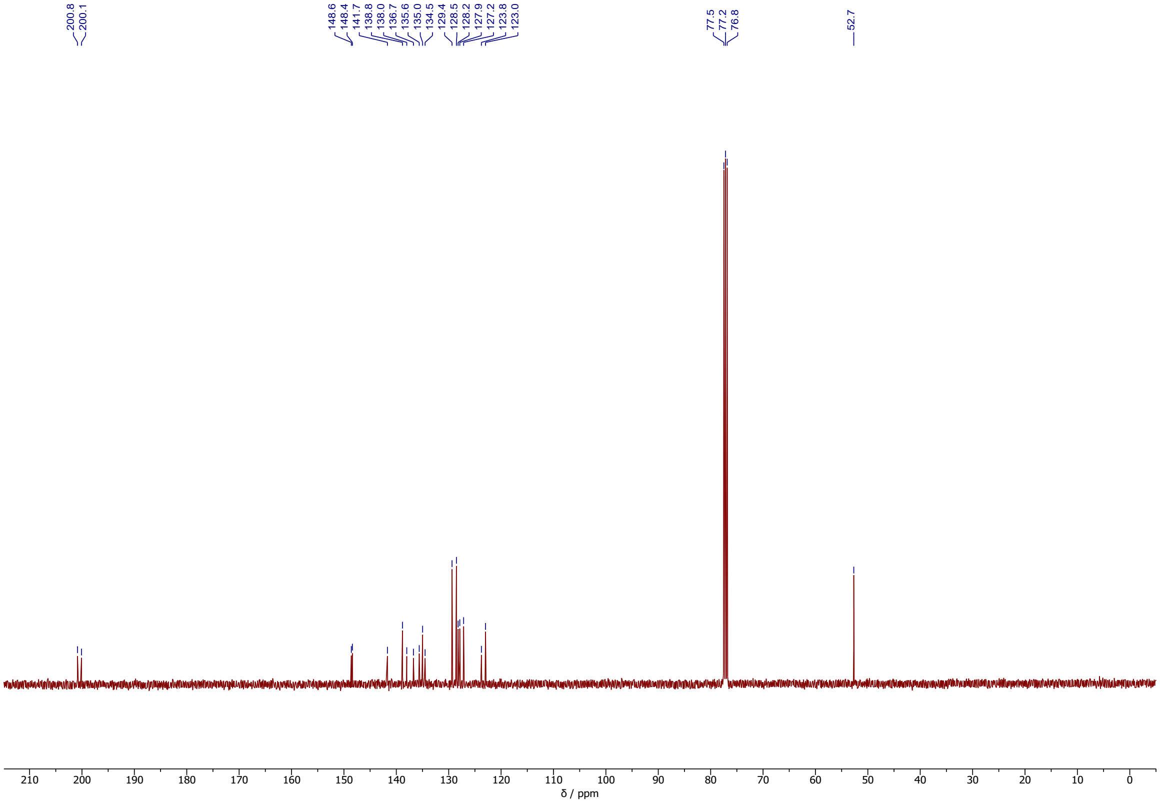

Figure S2. ^{13}C NMR spectrum (100 MHz, $CDCl_3$) of **2**.

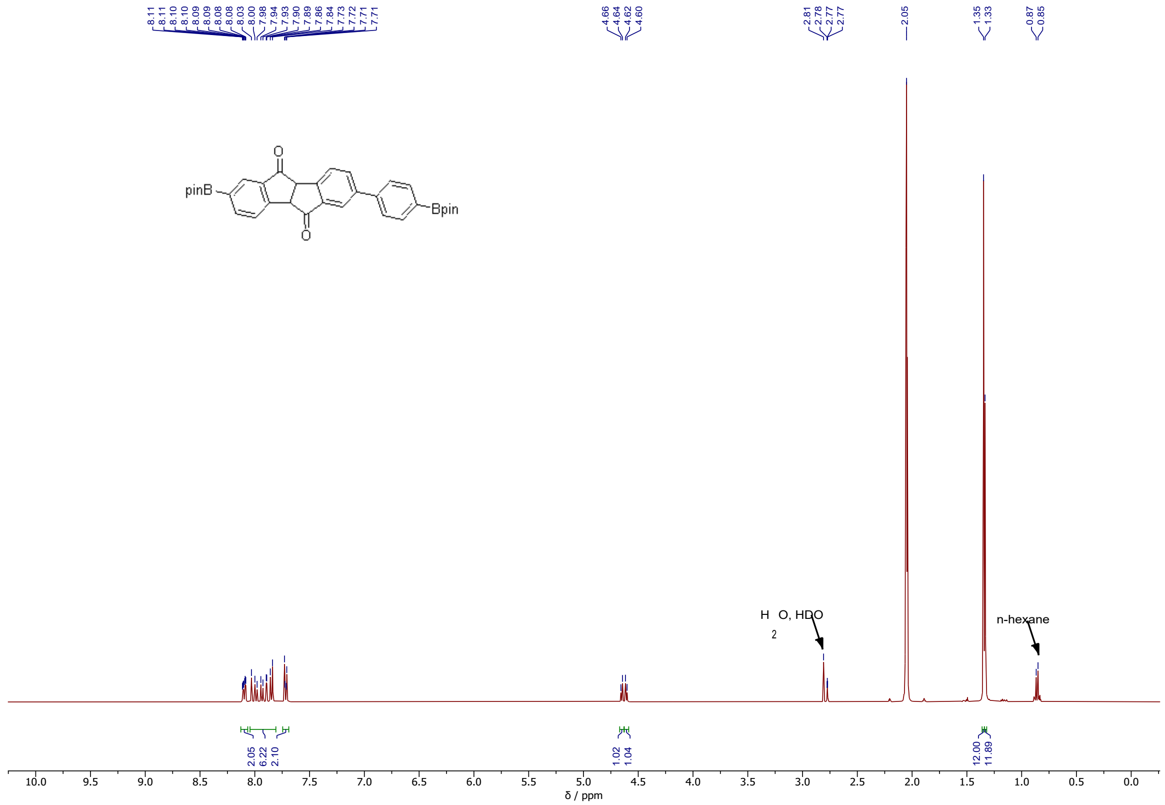

Figure S3. ^{1}H NMR spectrum (400 MHz, $(CD_3)_2CO$) of **3**.

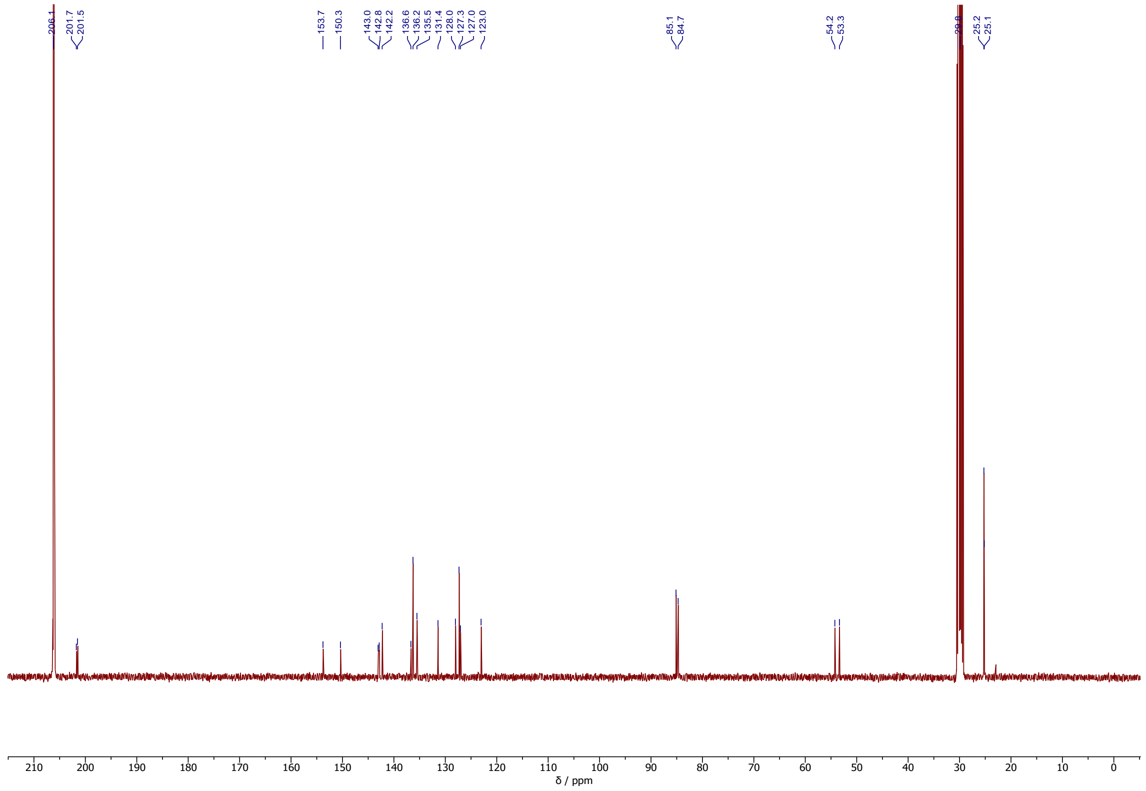

Figure S4. ^{13}C NMR spectrum (100 MHz, $(CD_3)_2CO$) of **3**.

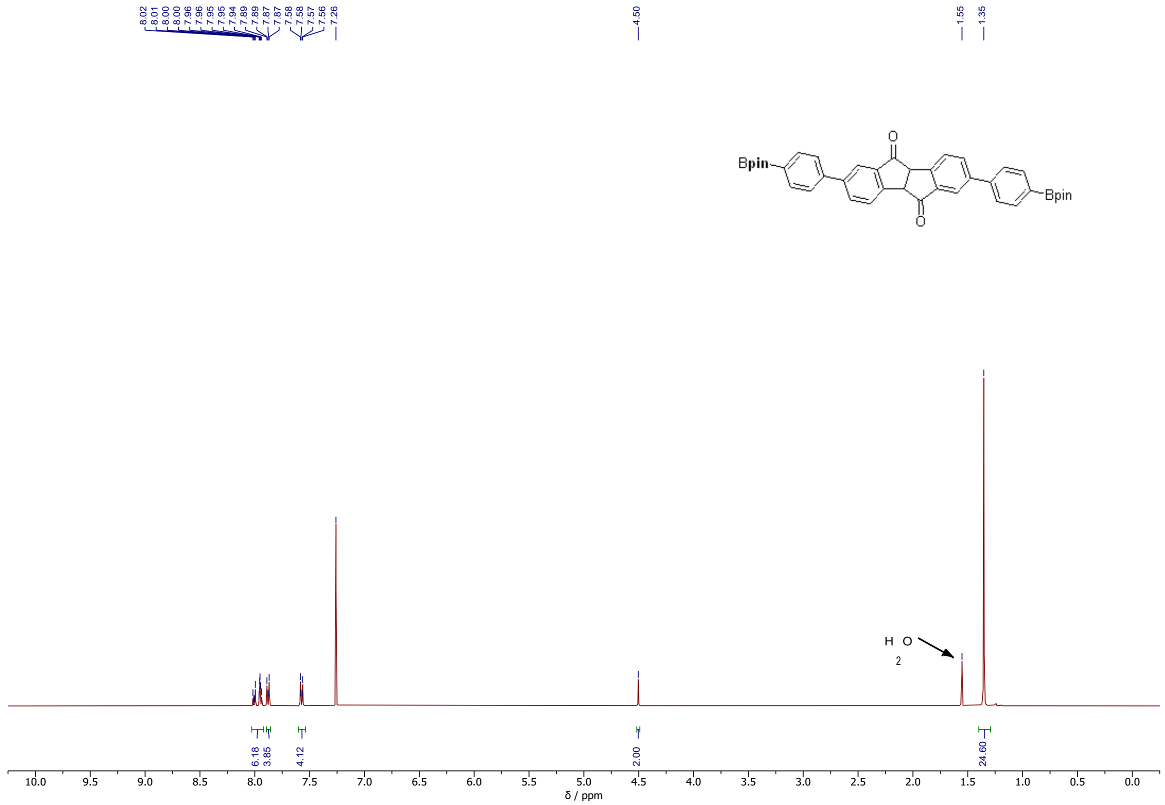

Figure S5. ^{1}H NMR spectrum (400 MHz, $CDCl_3$) of **4**.

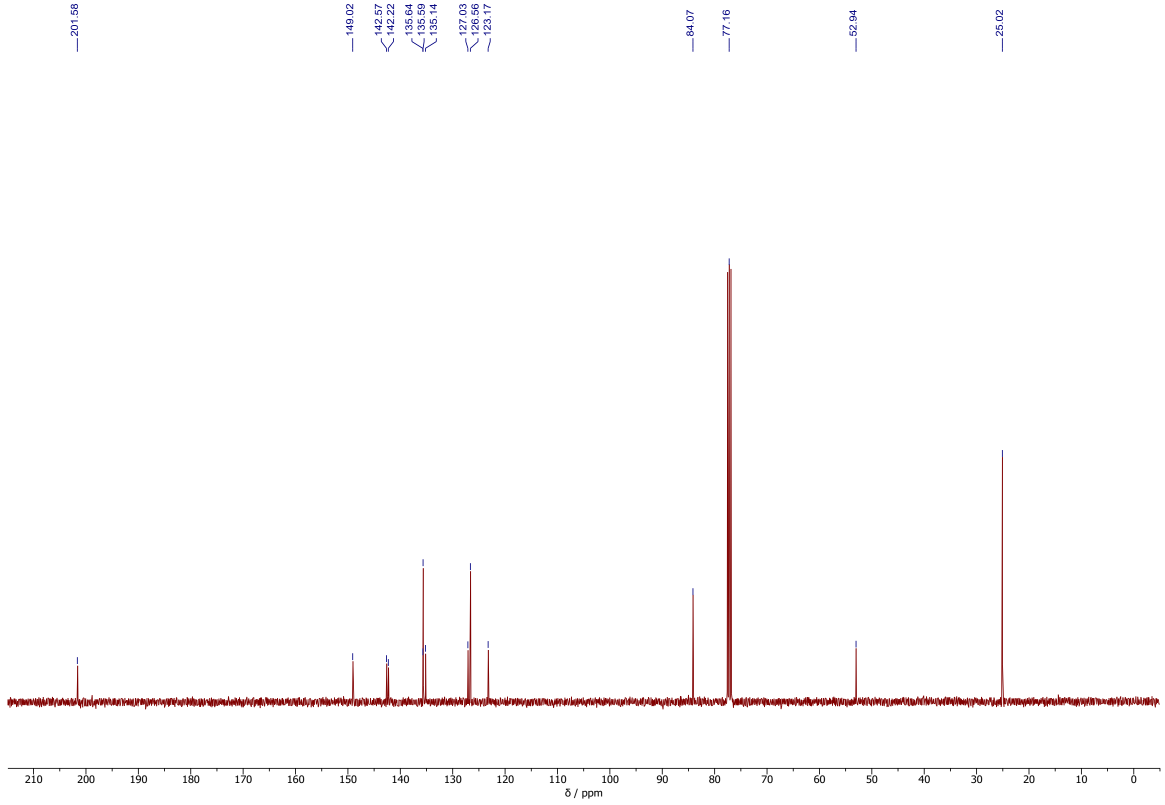

Figure S6. ^{13}C NMR spectrum (100 MHz, $CDCl_3$) of **4**.

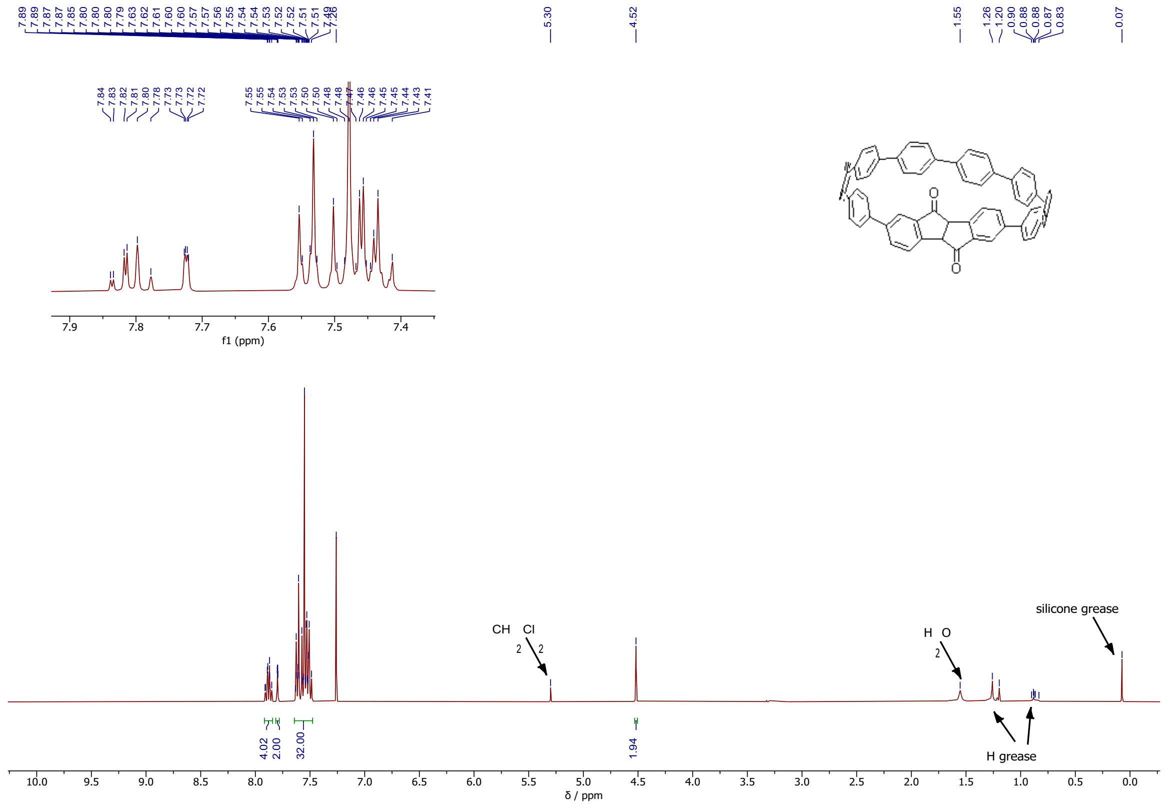

Figure S7. ^{1}H NMR spectrum (400 MHz, $CDCl_3$) of Diketo[8]CPP.

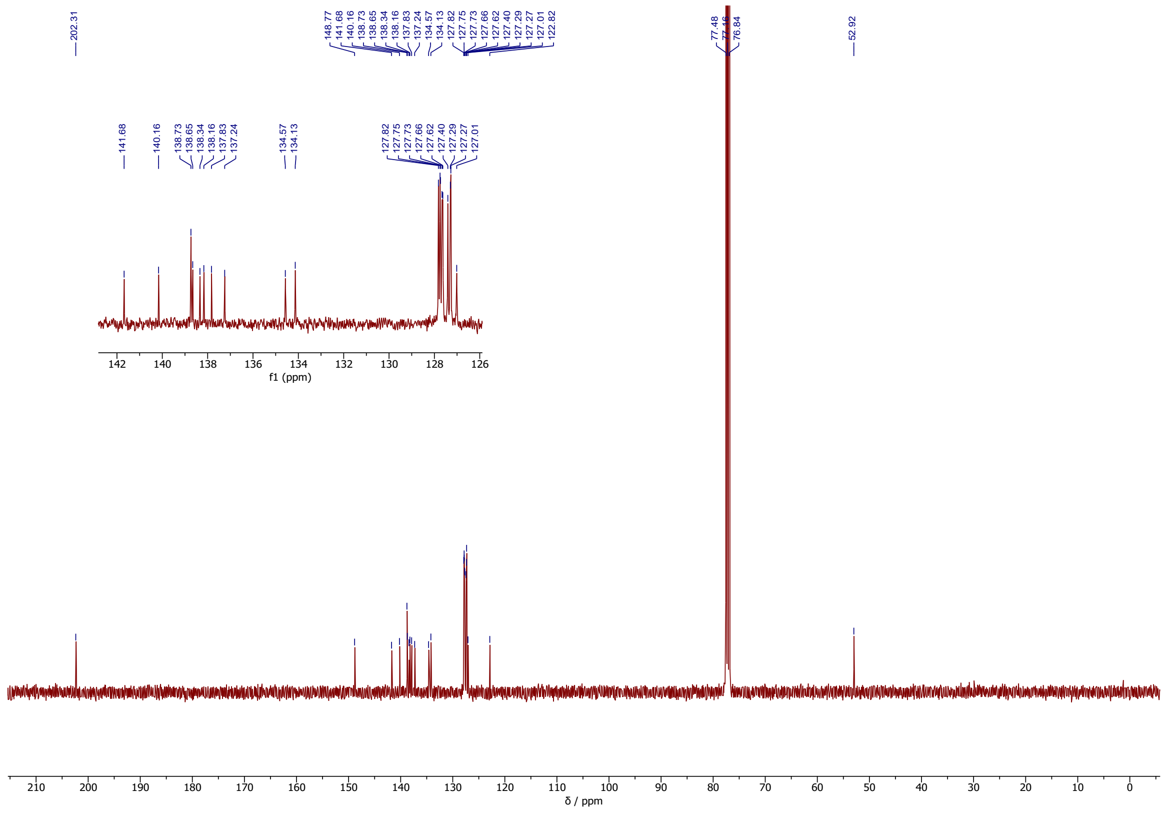

Figure S8. ^{13}C NMR spectrum (100 MHz, $CDCl_3$) of Diketo[8]CPP.

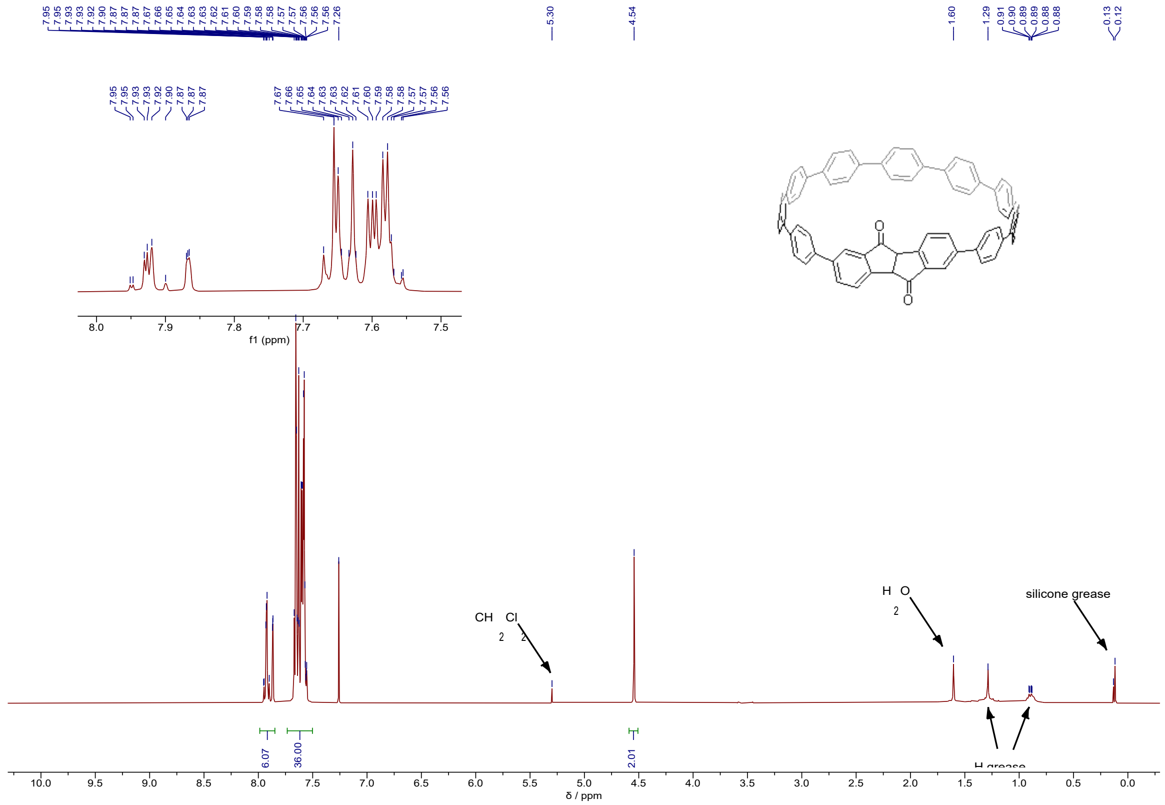

Figure S9. ^{1}H NMR spectrum (400 MHz, $CDCl_3$) of Diketo[9]CPP.

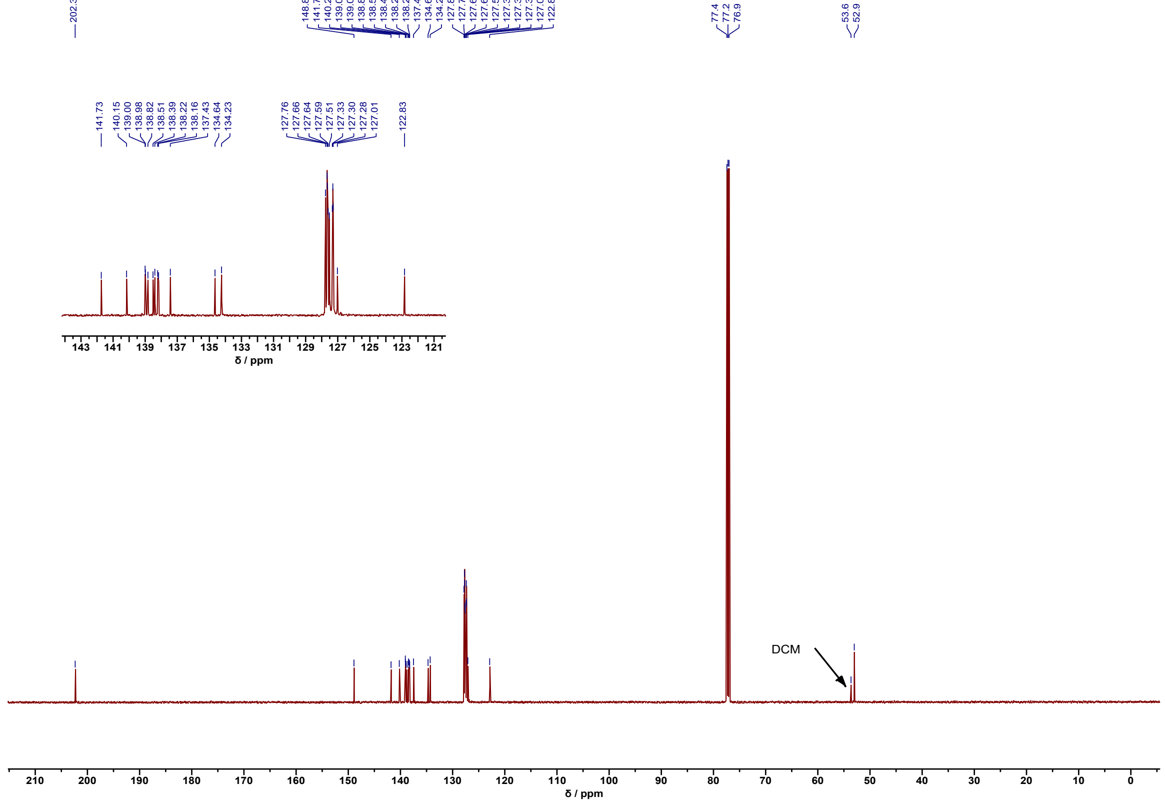

Figure S10. ^{13}C NMR spectrum (150 MHz, $CDCl_3$) of Diketo[9]CPP.

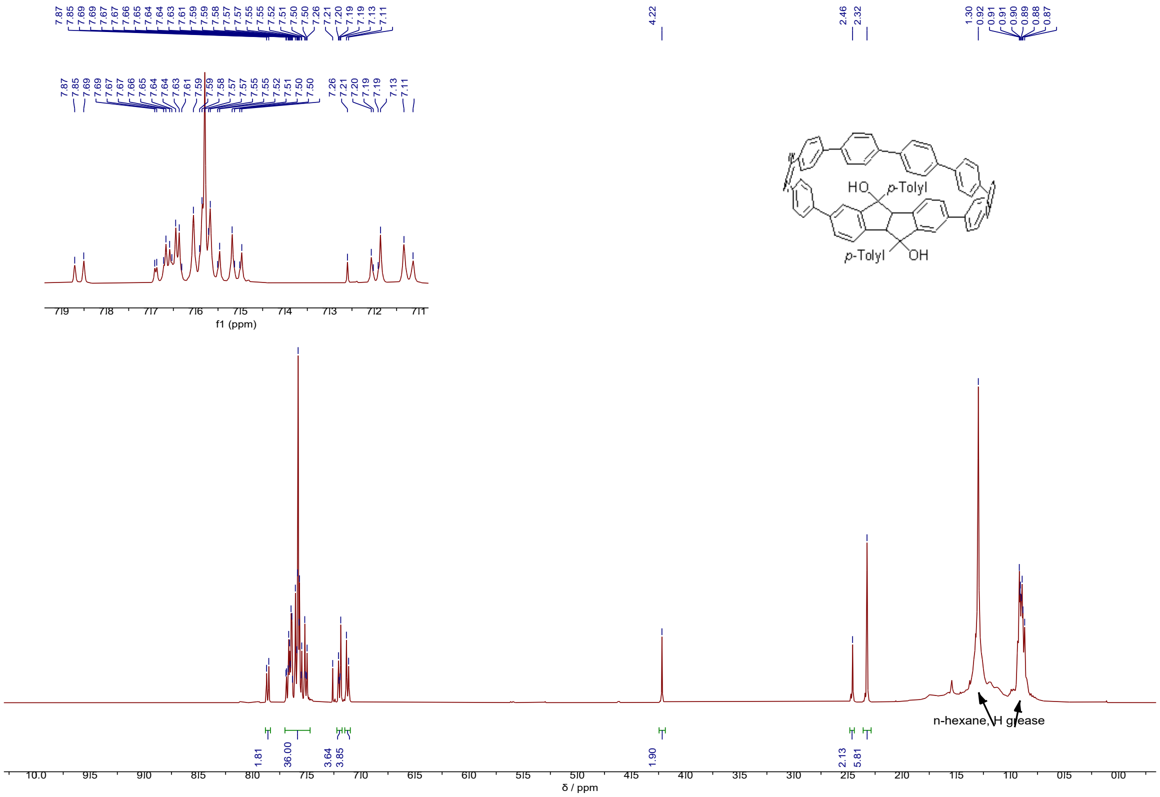

Figure S11. ^{1}H NMR spectrum (400 MHz, $CDCl_3$) of **8**.

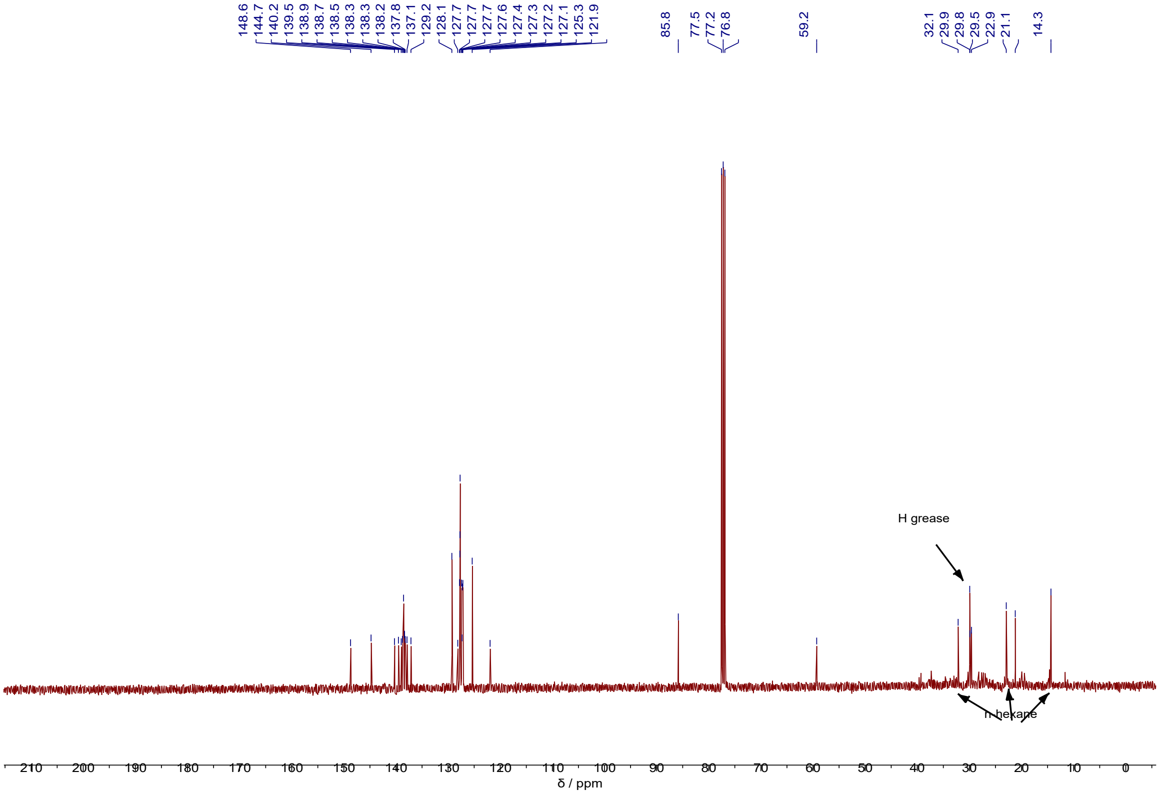

Figure S12. ^{13}C NMR spectrum (100 MHz, $CDCl_3$) of **8**.

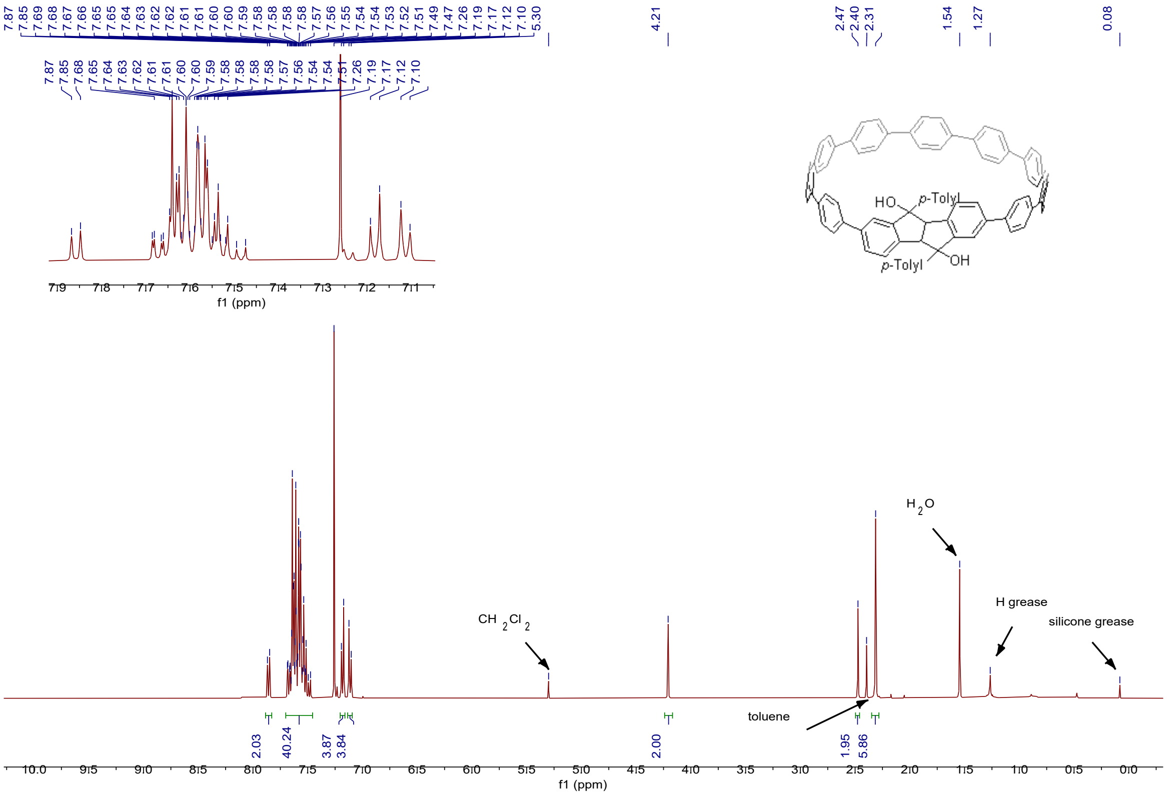

Figure S13. ^{1}H NMR spectrum (400 MHz, $CDCl_3$) of **9**.

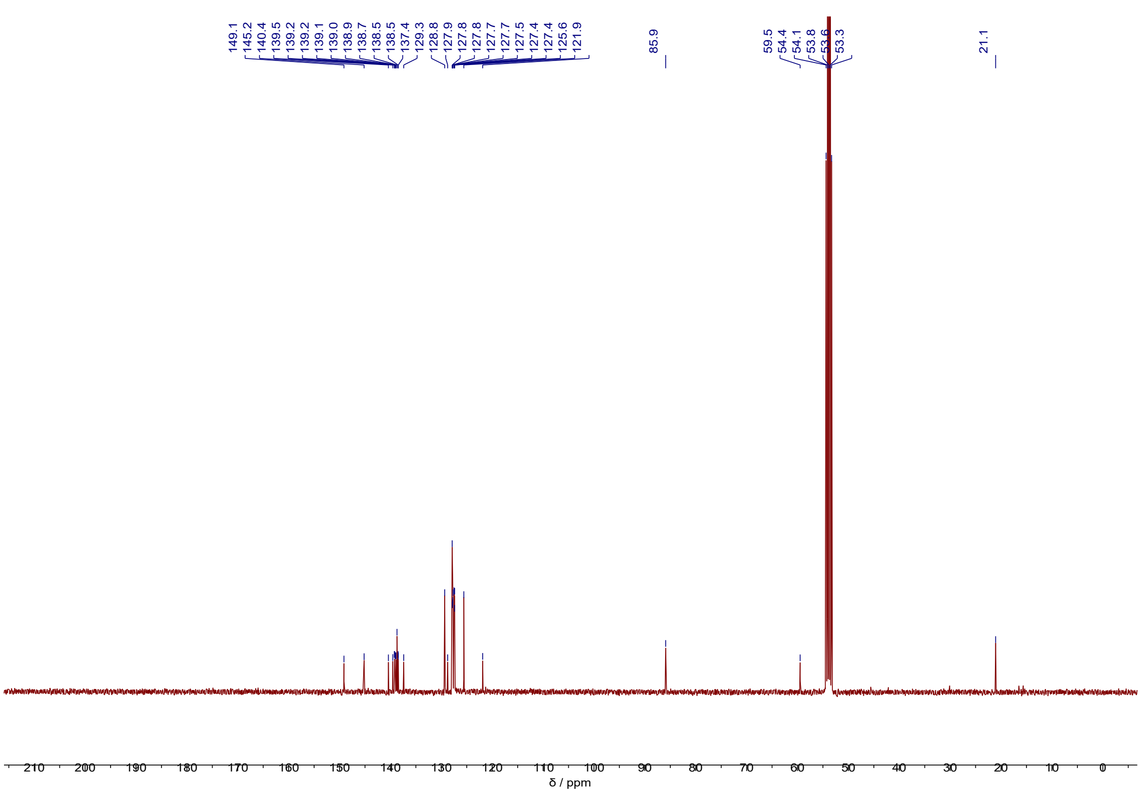

Figure S14. ^{13}C NMR spectrum (400 MHz, CD_2Cl_2) of **9**.

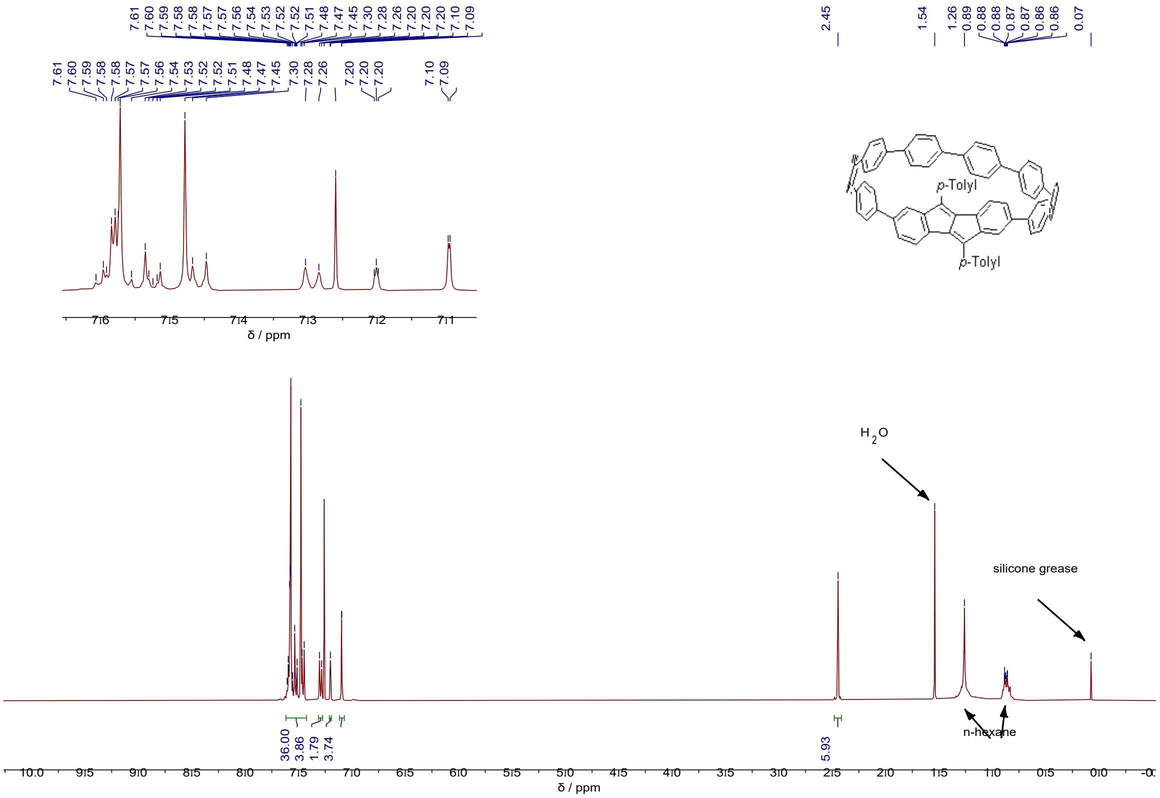

Figure S15. ^{1}H NMR spectrum (400 MHz, $CDCl_3$) of DBP[8]CPP.

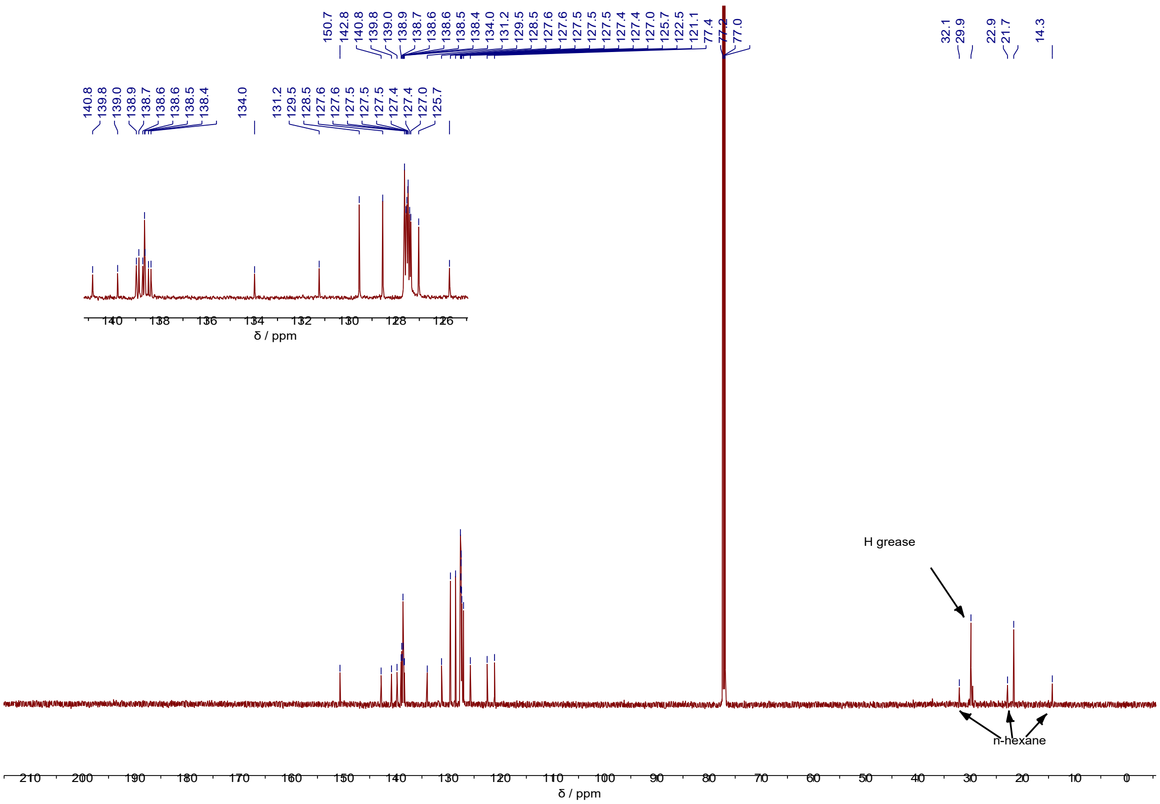

Figure S16. ^{13}C NMR spectrum (150 MHz, $CDCl_3$) of DBP[8]CPP.

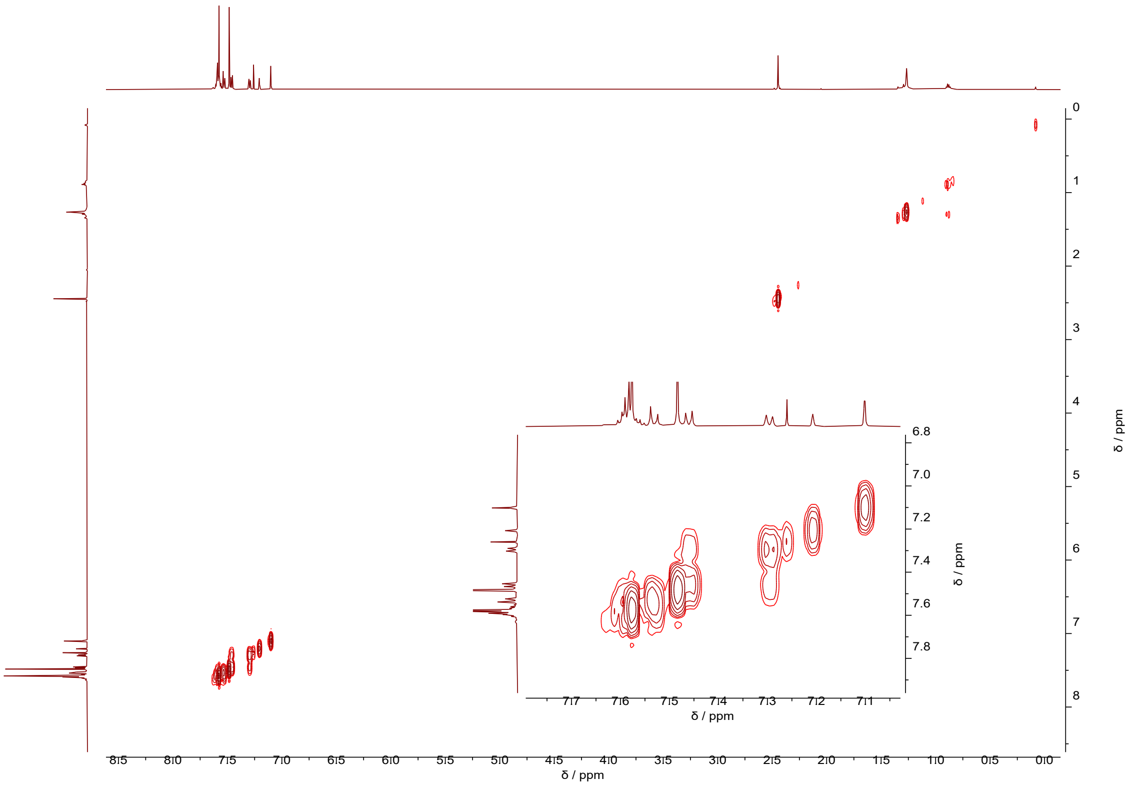

Figure S17. $^{1}H^{1}H$-COSY spectrum (600 MHz, $CDCl_3$) of DBP[8]CPP.

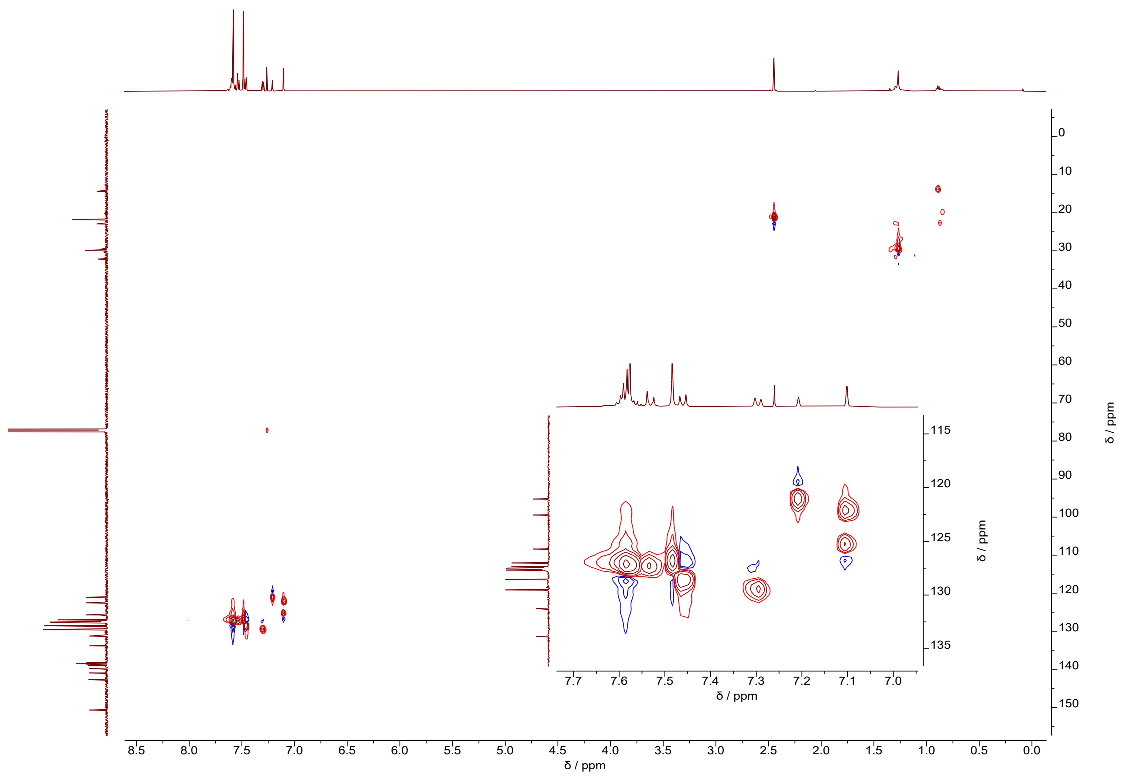

Figure S18**:** HSQC-spectrum of DBP[8]CPP in $CDCl_3$ (600/150 MHz).

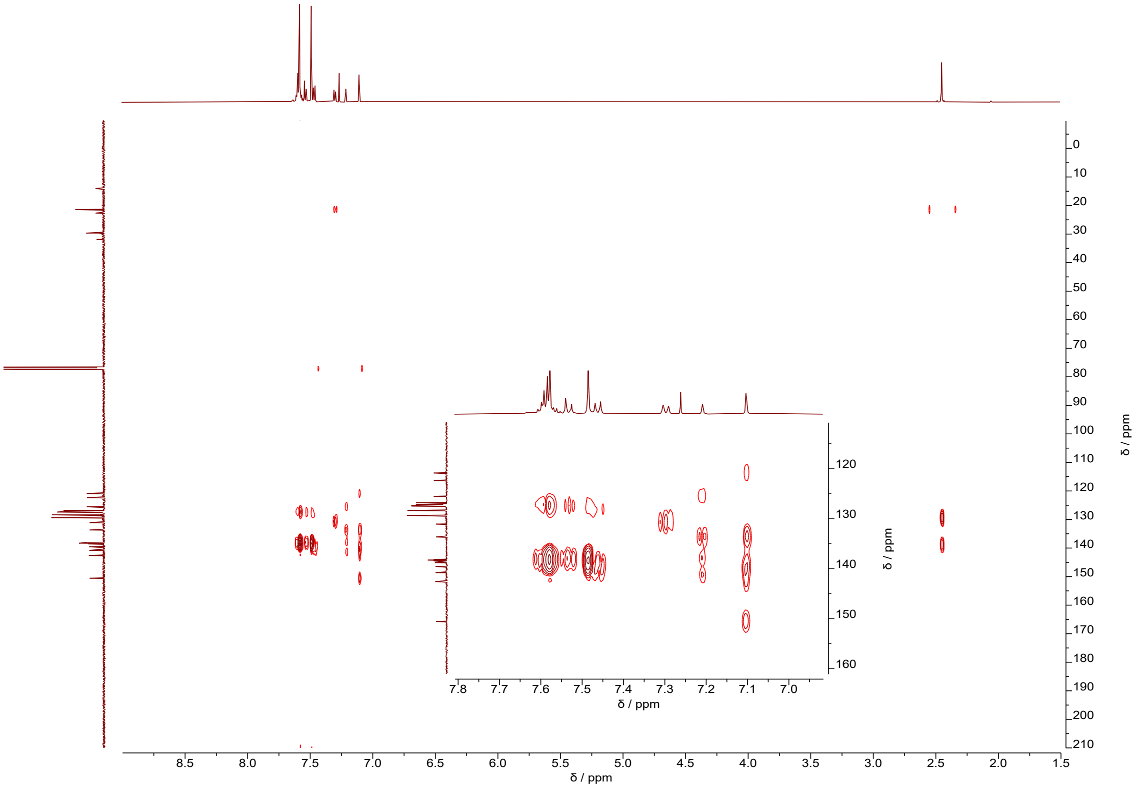

Figure S19**:** HMBC-spectrum of DBP[8]CPP in $CDCl_3$ (600/150 MHz).

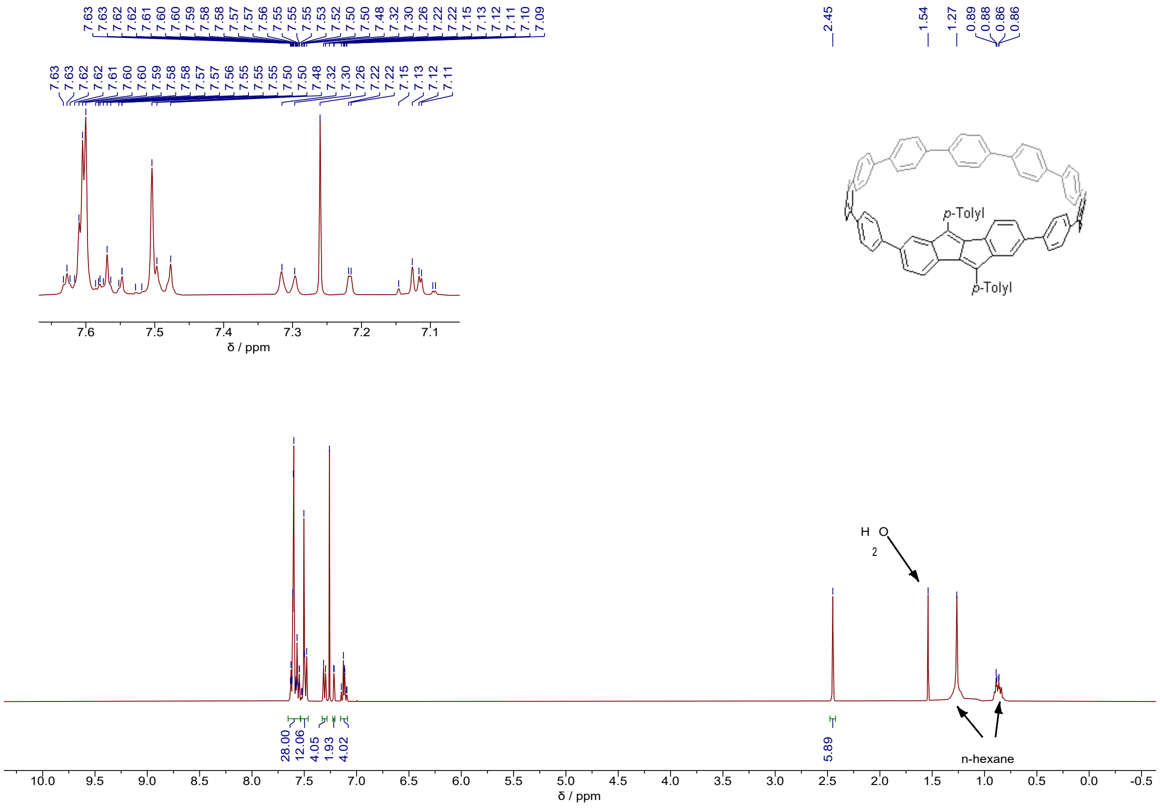

Figure S20. ^{1}H NMR spectrum (400 MHz, $CDCl_3$) of DBP[9]CPP.

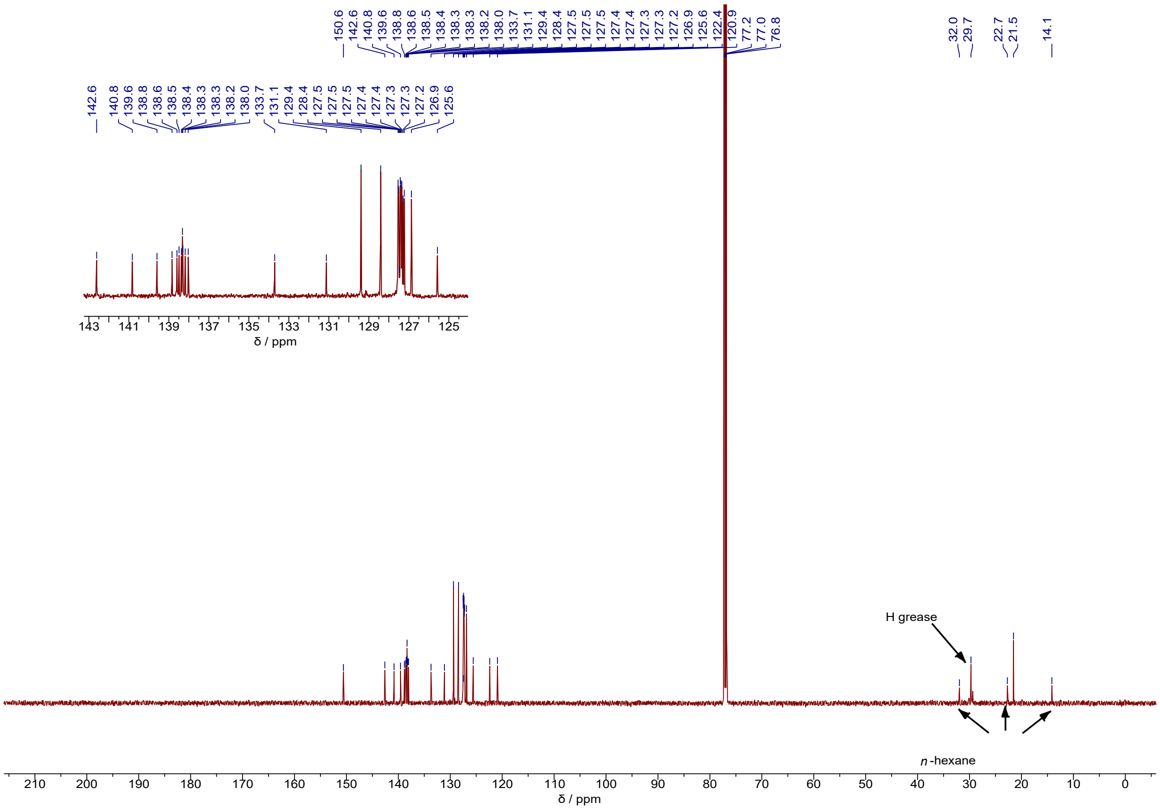

Figure S21. ^{13}C NMR spectrum (150 MHz, $CDCl_3$) of DBP[9]CPP.

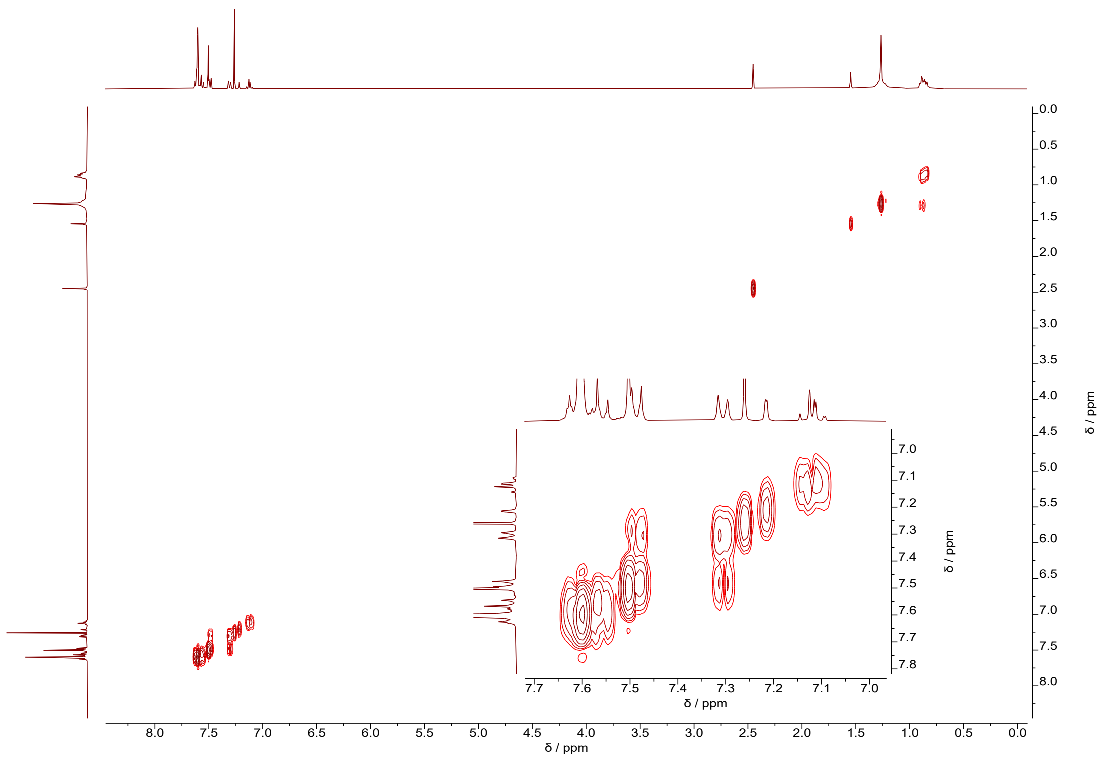

Figure S22. $^{1}H^{1}H$-COSY spectrum (400 MHz, $CDCl_3$) of DBP[9]CPP.

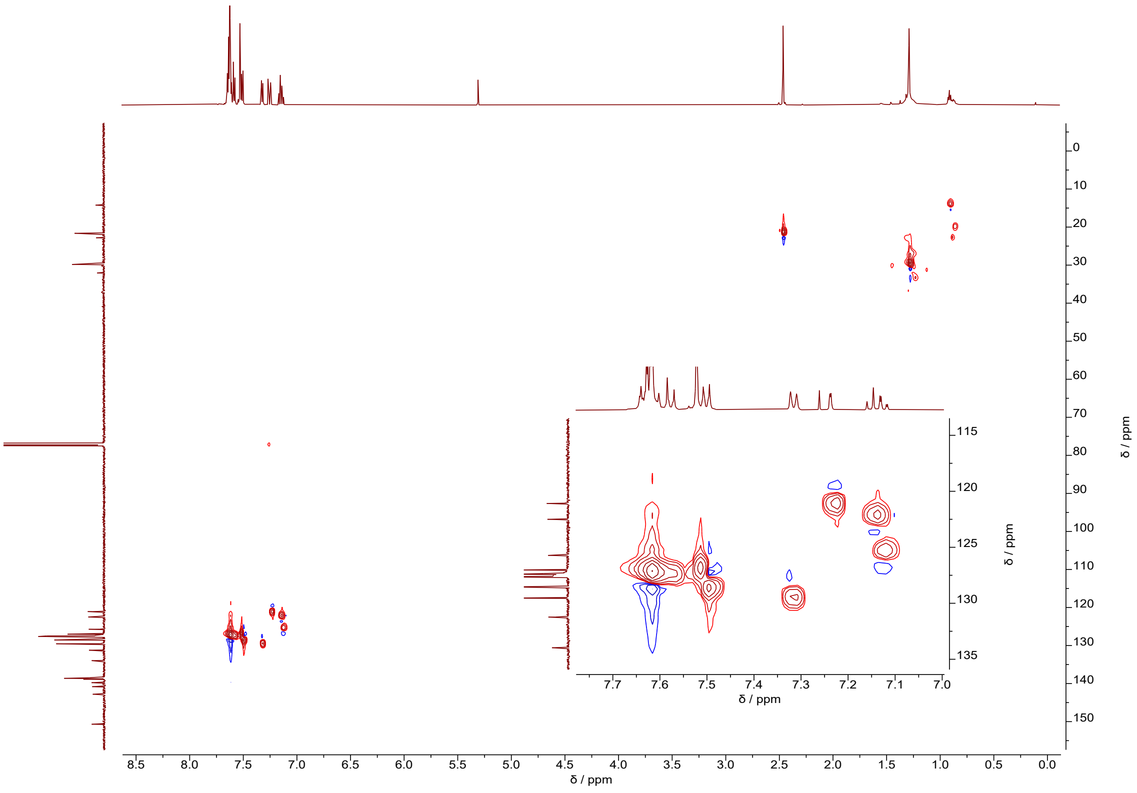

Figure S23**:** HSQC-spectrum of DBP[9]CPP in $CDCl_3$ (600/150 MHz).

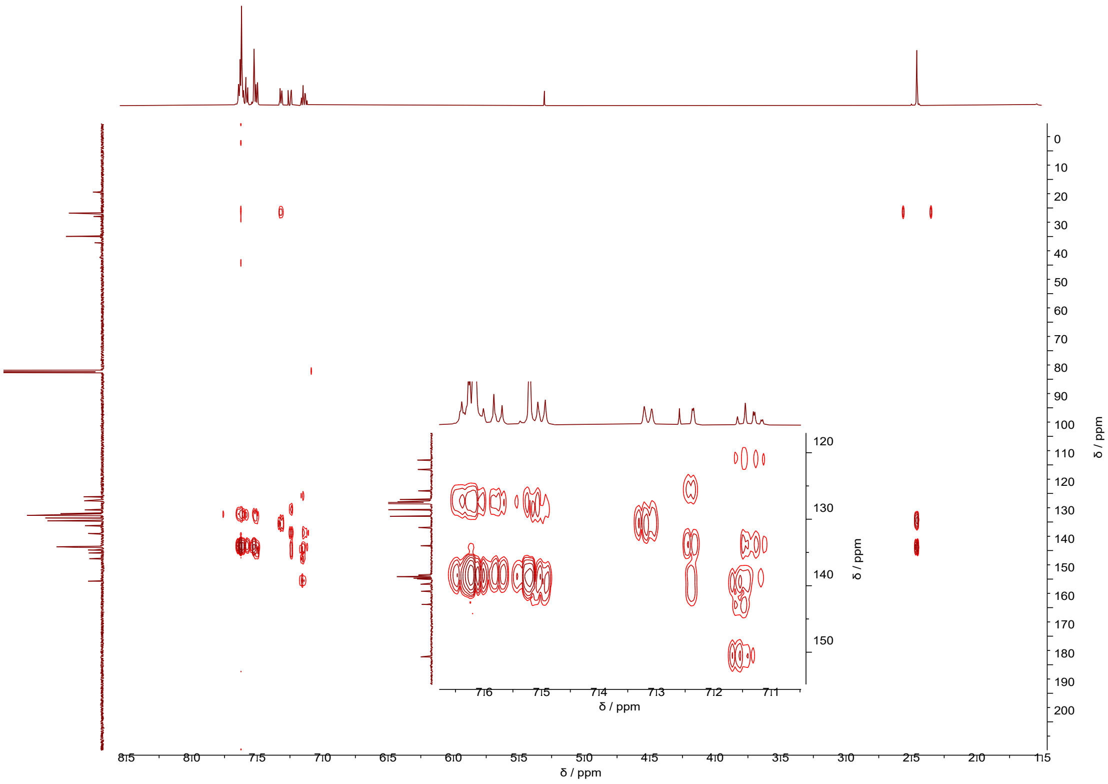

Figure S24**:** HMBC-spectrum of DBP[9]CPP in $CDCl_3$ (600/150 MHz).

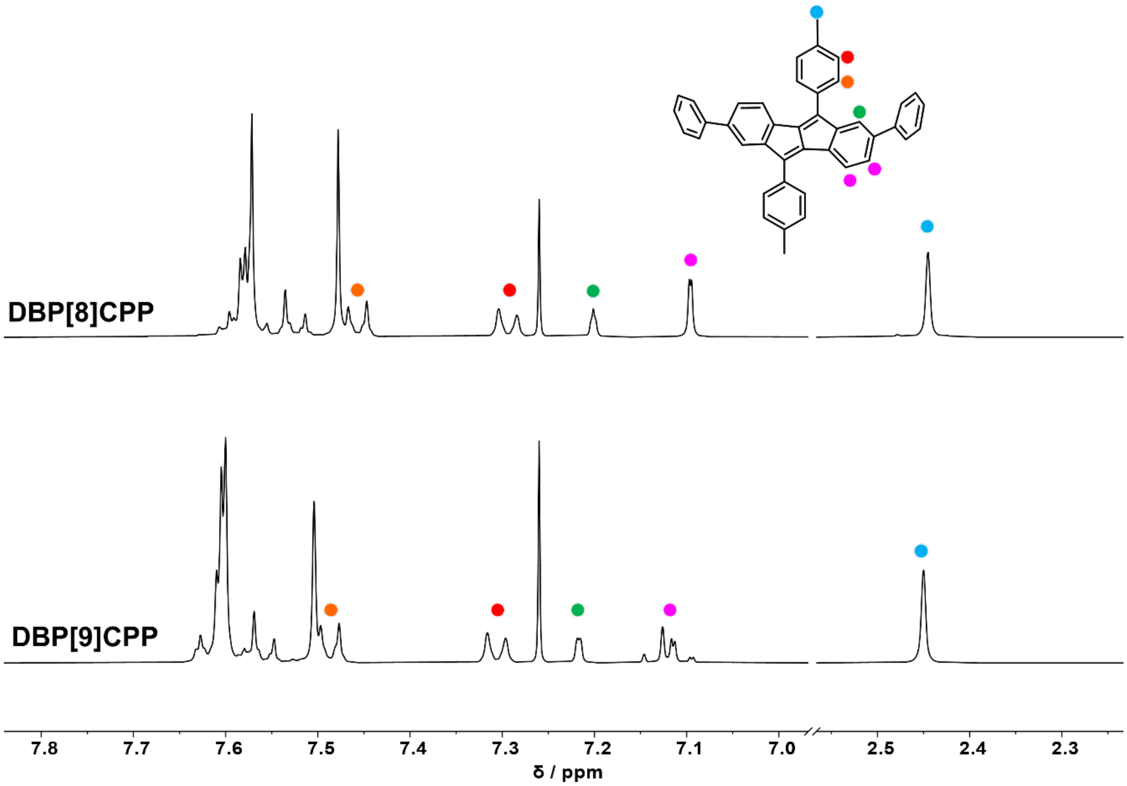

Figure S25: ^{1}H NMR spectra with signals assigned based on 2D NMR spectra of DBP[*n*]CPPs ($CDCl_3$, 400 MHz, 298 K).

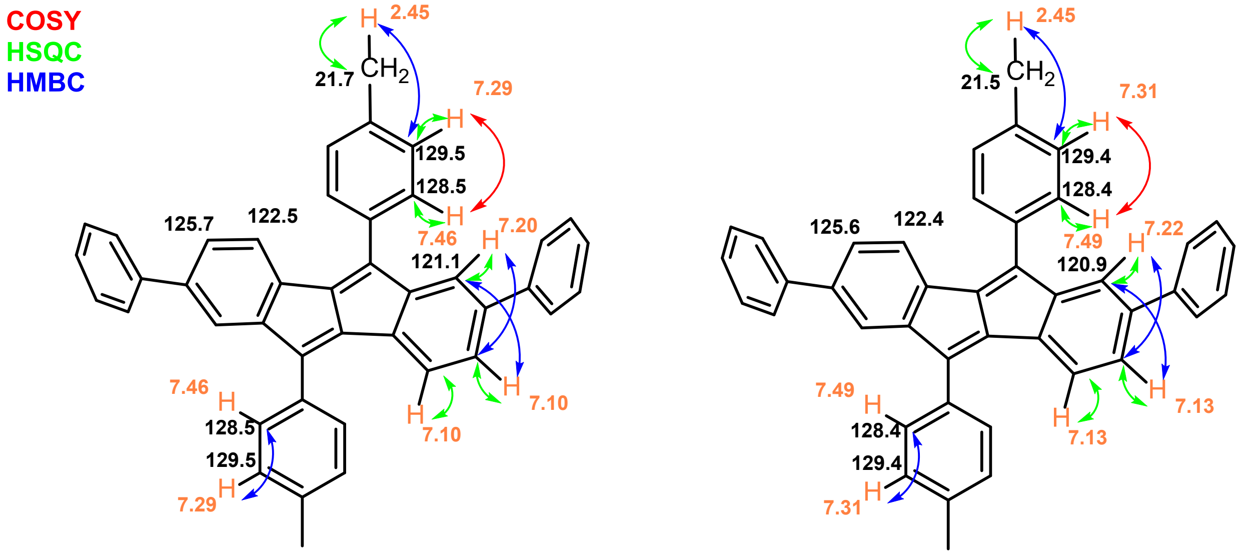

DBP[8]CPP **DBP[9]CPP**

Figure S26: Summary of the key correlations and assigned chemical shifts for DBP[*n*]CPPs.

4. Mass Spectra

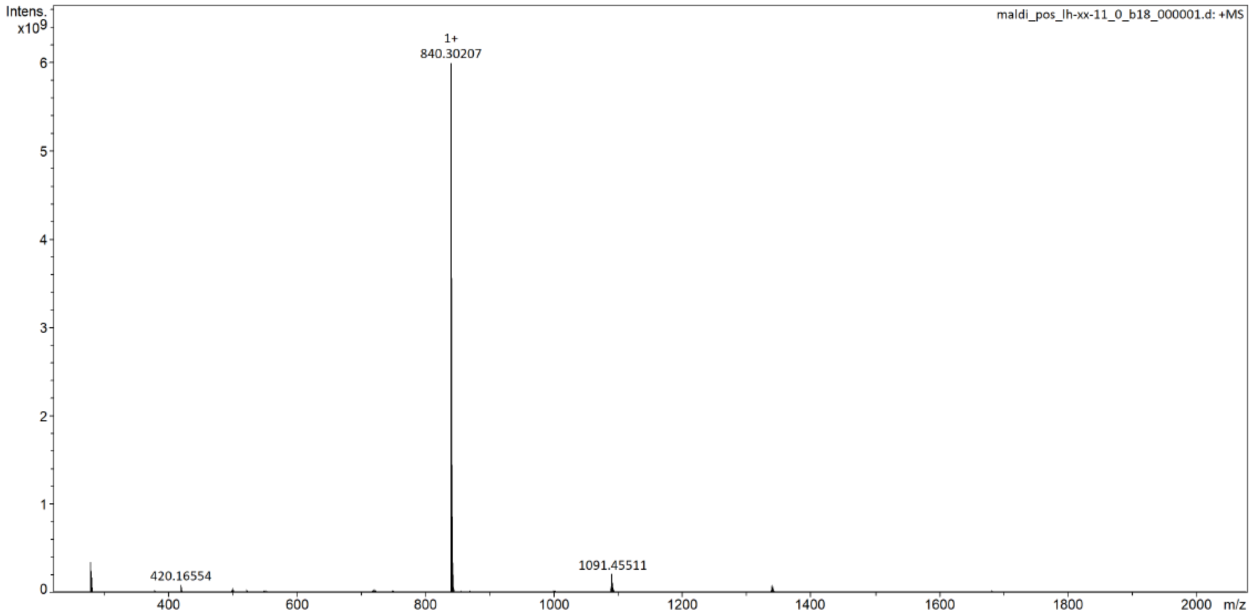

Figure S27. HRMS mass spectrum of diketo[8]CPP (MALDI, positive mode).

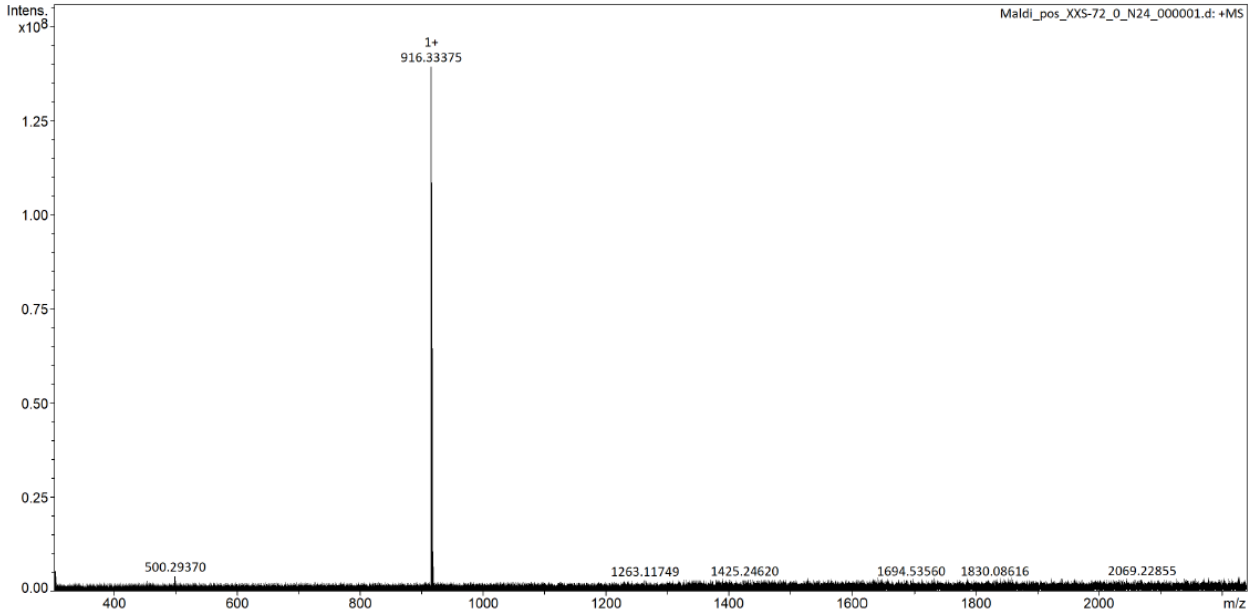

Figure S28. HRMS mass spectrum of diketo[9]CPP (MALDI, positive mode).

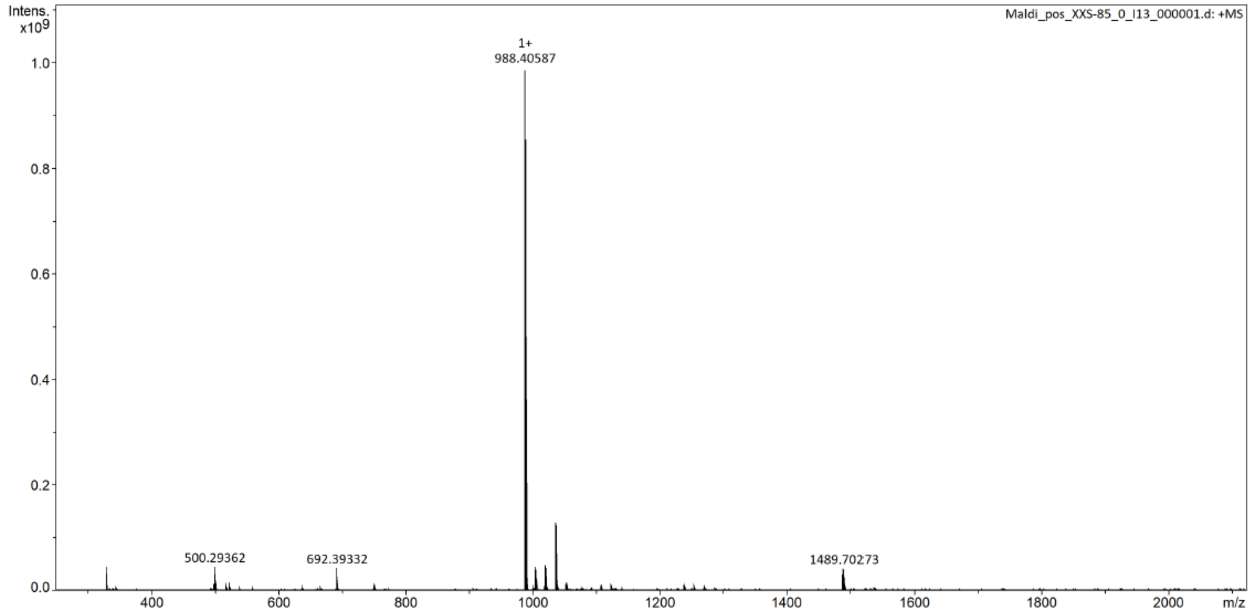

Figure S29. HRMS mass spectrum of DBP[8]CPP (MALDI, positive mode).

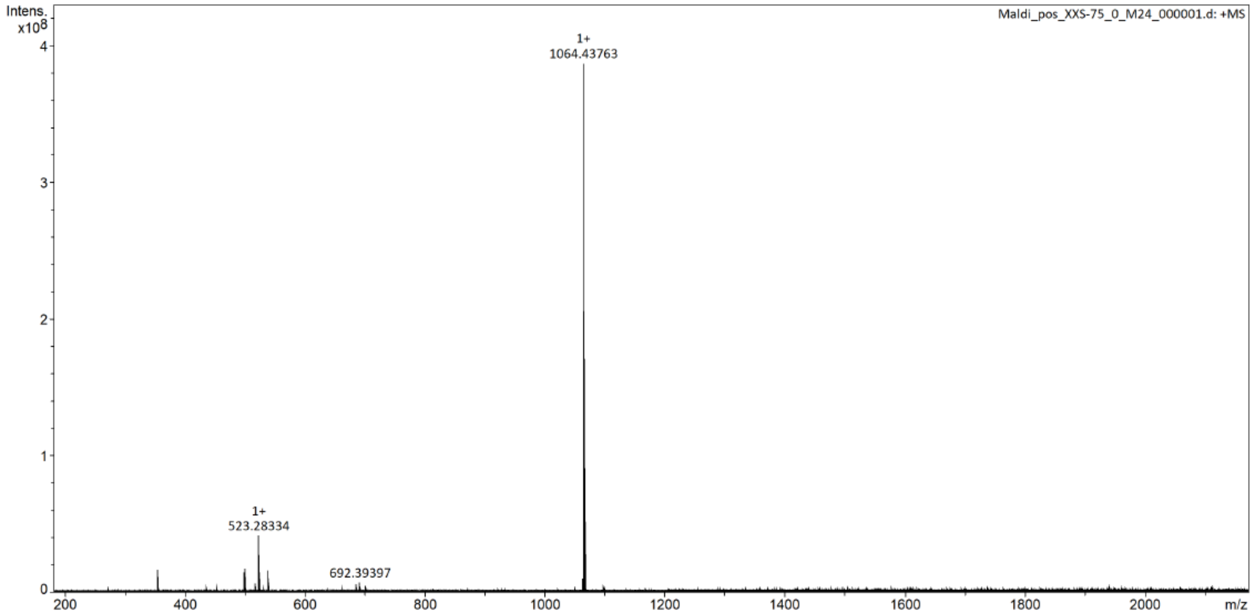

Figure S30. HRMS mass spectrum of DBP[9]CPP (MALDI, positive mode).

5. Optical Properties

5.1 Determination of Molar Extinction Coefficients

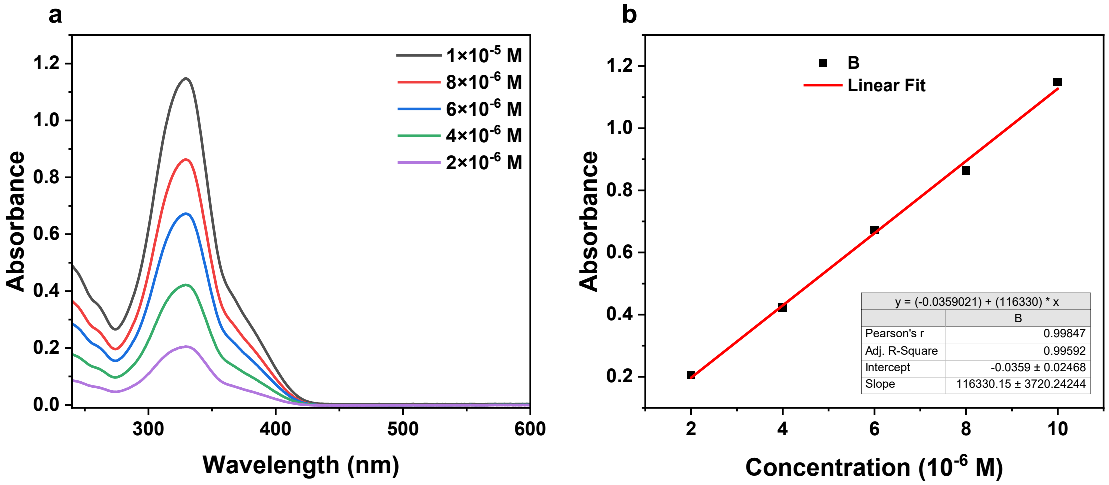

Figure S31. (a) Absorbance spectra of diketo[8]CPP at different concentrations in CH_2Cl_2. (b) Absorbance *vs*. concentration plot of diketo[8]CPP at ~329 nm.

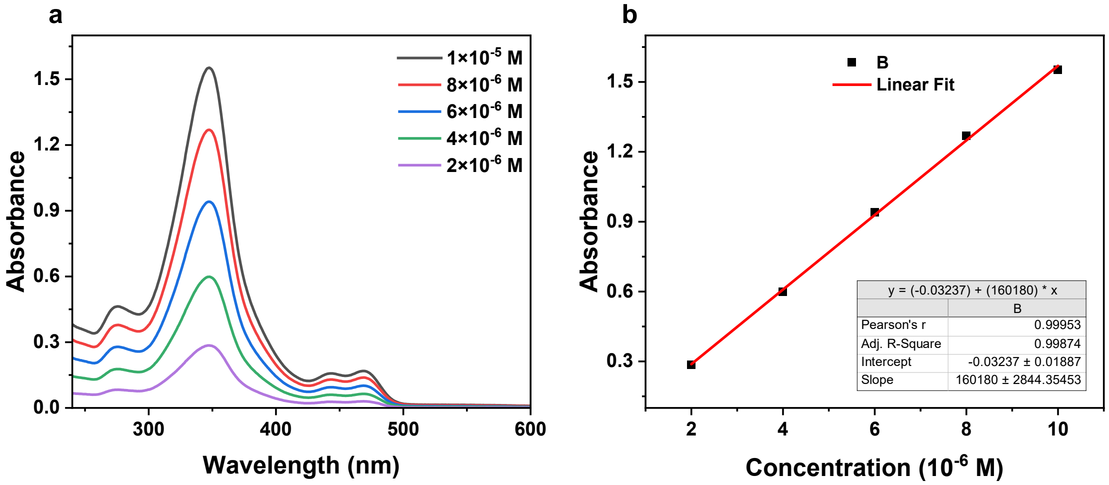

Figure S32. (a) Absorbance spectra of DBP[8]CPP at different concentrations in CH_2Cl_2. (b) Absorbance *vs*. concentration plot of DBP[8]CPP at ~347 nm.

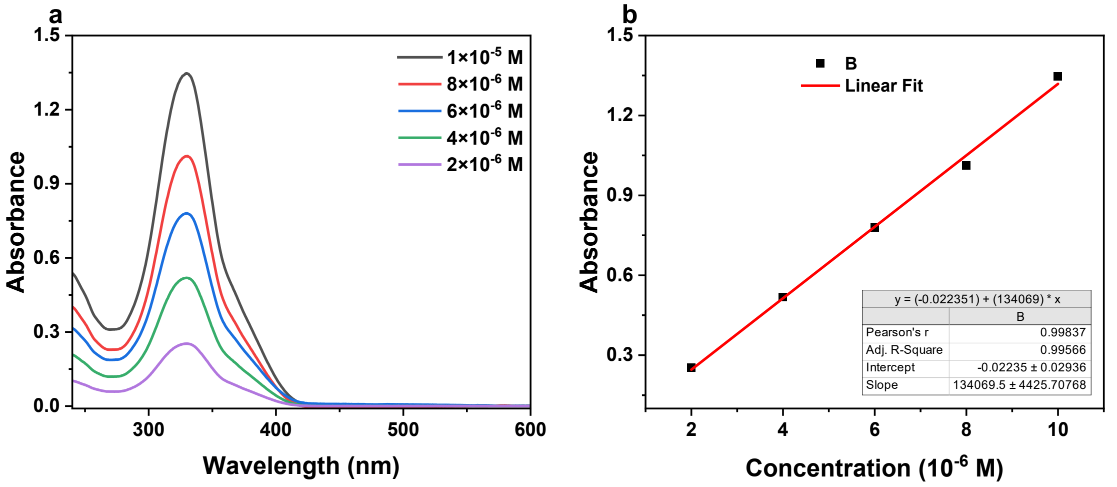

Figure S33. (a) Absorbance spectra of diketo[9]CPP at different concentrations in CH_2Cl_2. (b) Absorbance *vs.* concentration plot of diketo[9]CPP at ~330 nm.

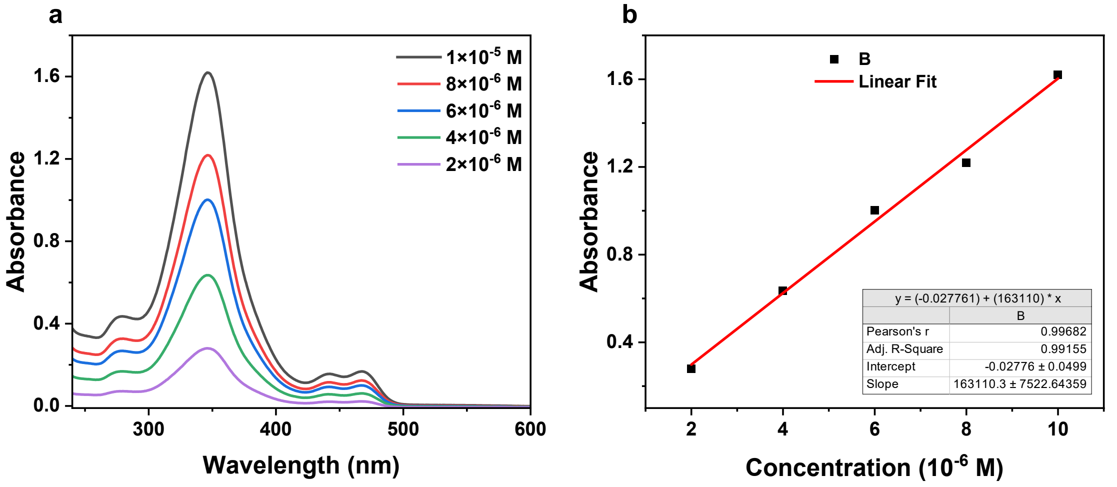

Figure S34. (a) Absorbance spectra of DBP[9]CPP at different concentrations in CH_2Cl_2. (b) Absorbance *vs.* concentration plot of compound DBP[9]CPP at ~346 nm.

5.2 Absorption and Emission Spectra

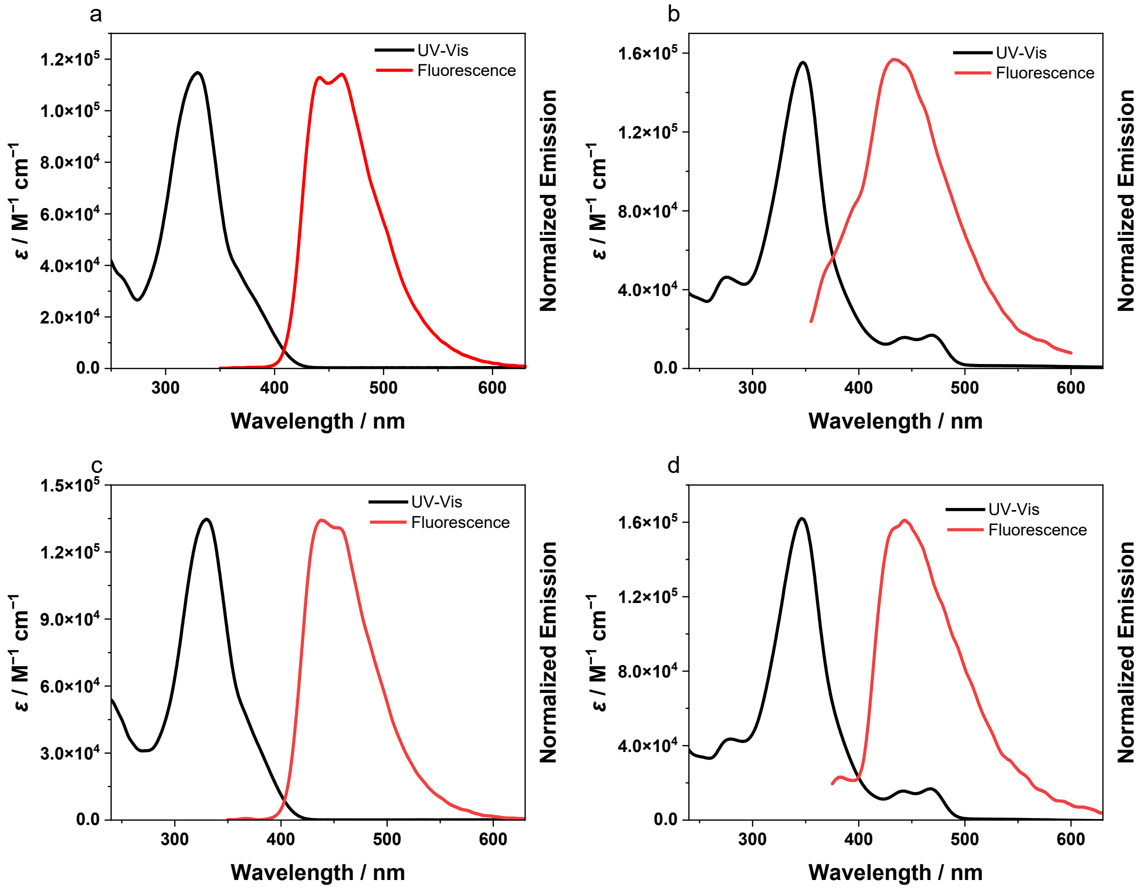

Figure S35. Absorption and emission spectra (excitation wavelengths=330 nm) of (a) diketo[8]CPP, (b) DBP[8]CPP, (c) diketo[9]CPP and (d) DBP[9]CPP in CH_2Cl_2.

6. Chiral Resolution, Circular Dichroism and Circularly Polarized Luminescence Spectra

6.1 Chiral resolution and the chiroptical properties for diketo[n]CPP

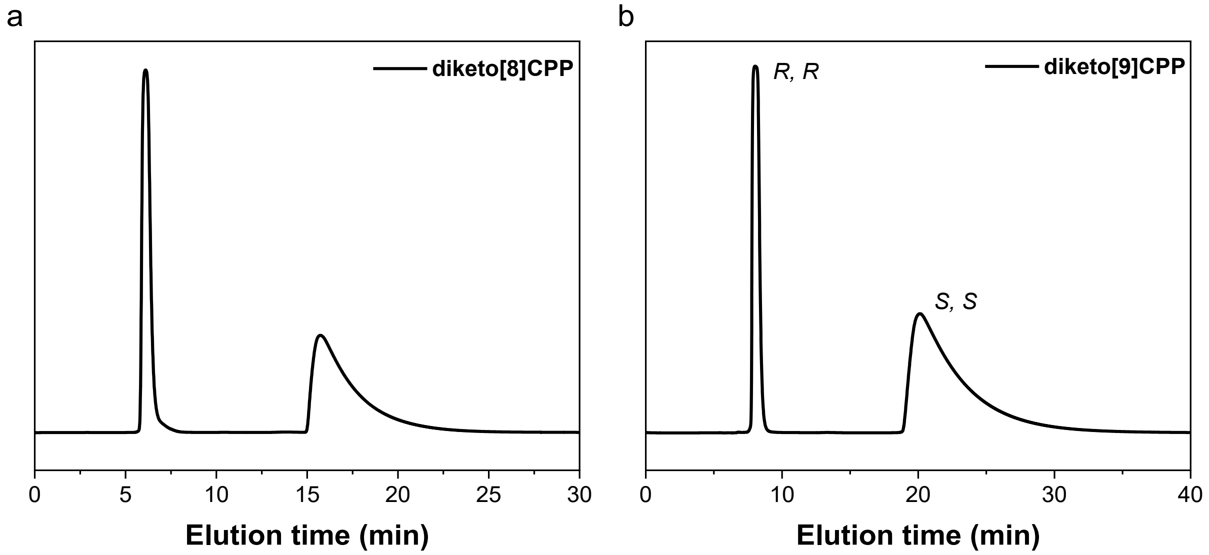

Figure S36. HPLC elugrams for racemic (a) diketo[8]CPP and (b) diketo[9]CPP. Chiral resolution conditions: column Daicel CHIRALPAK IG-5, 5 μm, column size: 10×250mm, mobile phase: CH_2Cl_2, flow rate: 5 mL/min. The detection wavelength was 320 nm.

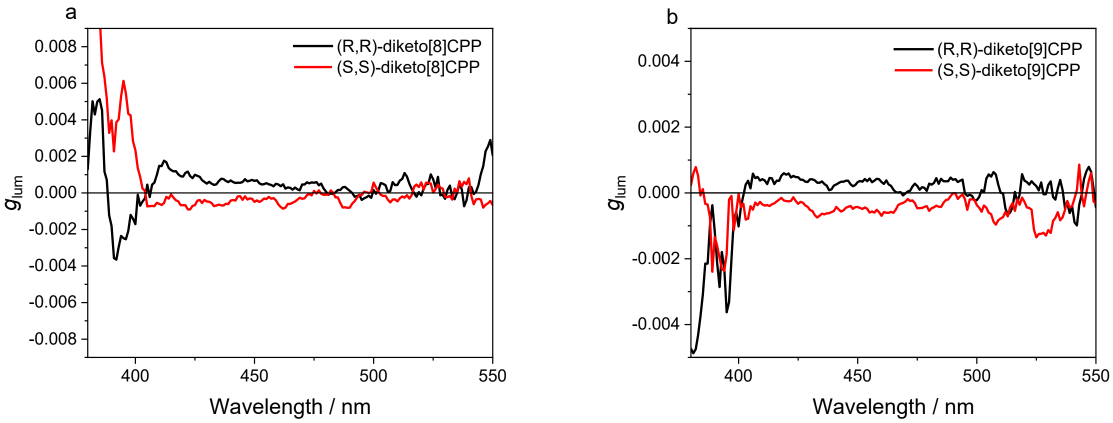

Figure S37. g_{lum}= f (λ) curves obtained for (*R*,*R*)-diketo[*n*]CPP and (*S*,*S*)-diketo[*n*]CPP (DCM solutions at 5.0×10^{-5} M).

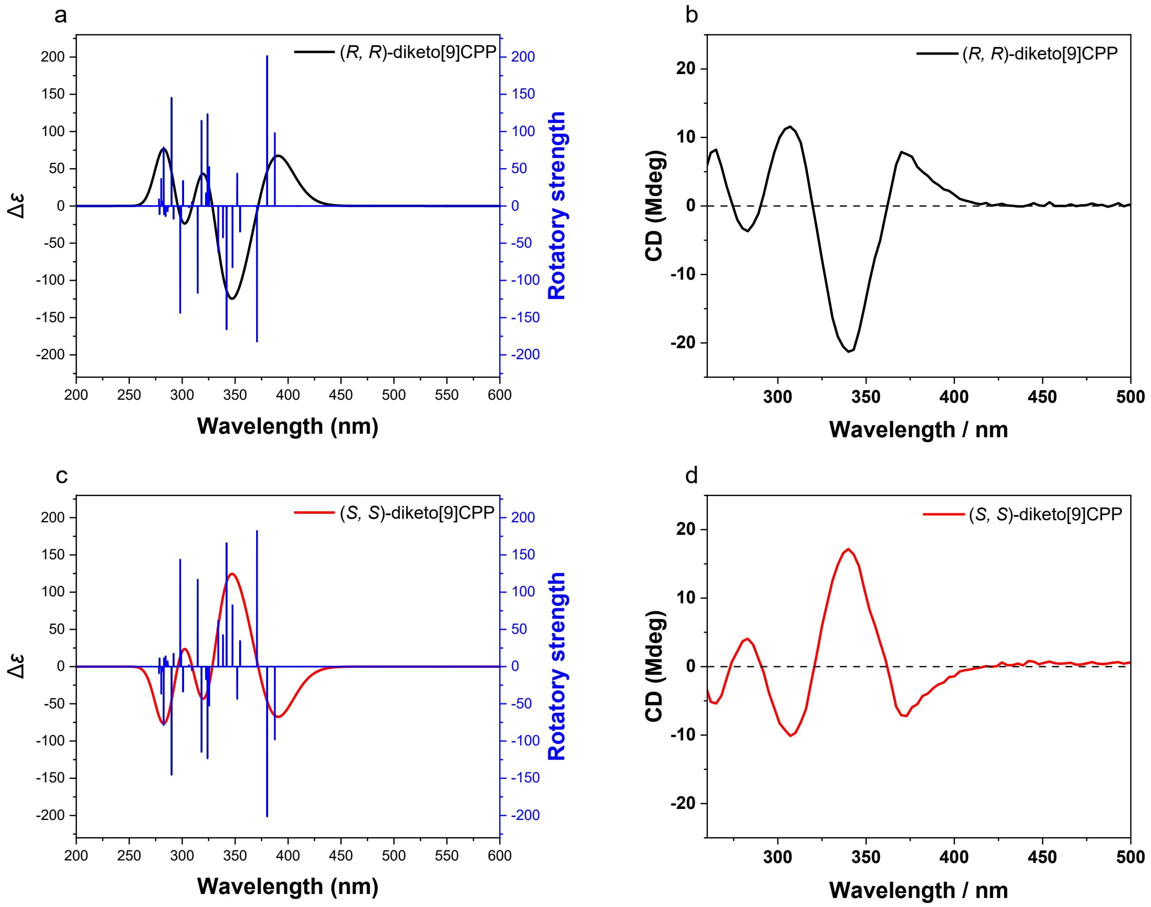

Figure S38. Theoretical (a, TD-DFT, B3-LYP-D3(BJ)/def2-TZVP) and experimental (b) CD spectra of (*R,R*)-diketo[9]CPP. Theoretical (c, TD-DFT, (B3-LYP-D3(BJ)/def2-TZVP) and experimental (d) CD spectra of (*S,S*)-diketo[9]CPP.

6.2 Dynamic HPLC elution profiles and the thermodynamic analysis for DBP[n]CPP

Elution profiles for the dynamic HPLC experiment of compound DBP[9]CPP were obtained using a Chiralpak IG-3 column (column size: 5×250mm) with a dichloromethane/n-hexane (4:1) mobile phase at a flow rate of 1.0 mL/min. All experiments were repeated three times at each temperature, only one representative example is shown. The elution profiles were analyzed at λ = 255 nm. The elution profile was analyzed with the DCXplorer software. [5]

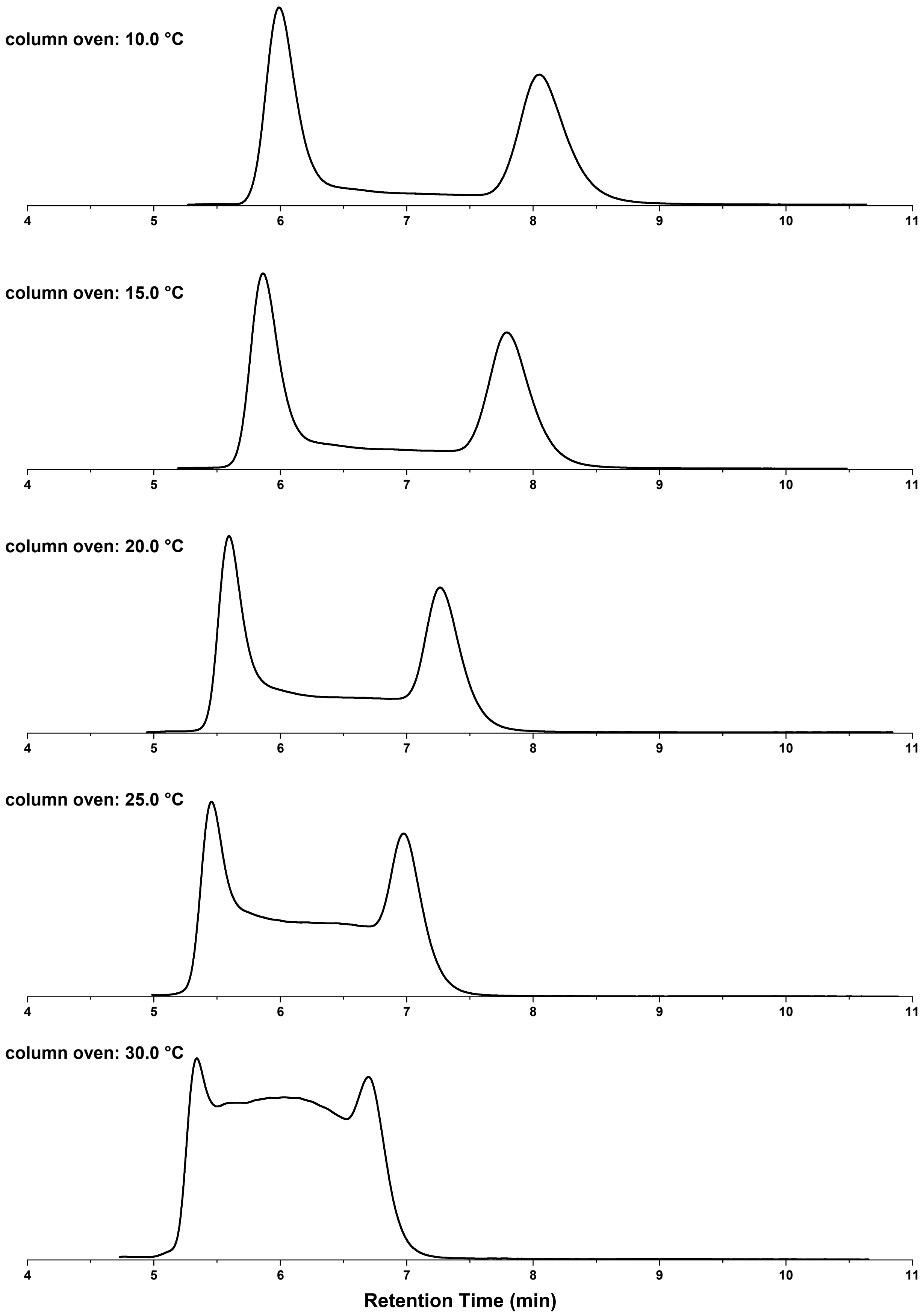
column oven: 10.0 °C
column oven: 15.0 °C
column oven: 20.0 °C
column oven: 25.0 °C
column oven: 30.0 °C
4
5
6
7
8
9
10
11
Retention Time (min)

Figure S39. Representative elution profiles obtained from the dynamic HPLC experiment at a temperature range from 10 – 30 °C.

Data evaluation with DCXplorer

The rate constants are obtained directly from the parameters given, via the software DCXplorer by Trapp and coworkers and are shown in the following table, together with the key parameters that are used for the determination.

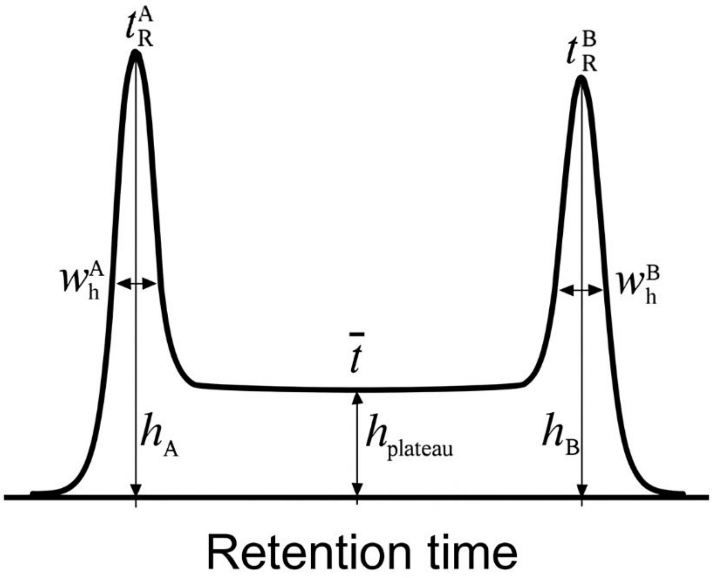

Figure S40. Typical chromatogram where the interconversion is occurring on the HPLC column, and the labelling of the parameters used for data fitting

Table S1. Obtained data from DCXplorer

	t_1^R [min]	t_2^R [min]	w_1^h [s]	w_2^h [s]	h_1 [%]	h_p [%]	h_2 [%]	A_1	A_2	k_e [1/s]
10 °C column										
First Elution	5.991	8.049	14.2	27.1	100.00	5.62	65.95	50.32	49.68	5.956E-04
Second Elution	5.991	8.051	14.5	27.8	100.00	5.62	66.86	50.27	49.73	5.820E-04
Third Elution	6.020	8.098	14.4	27.4	100.00	5.21	66.45	50.58	49.42	5.511E-04

k1 with error										5.76±0.23 E-04
15 °C column										
First Elution	5.863	7.792	13.4	24.6	100.00	9.79	69.76	50.23	49.77	9.942E-04
Second Elution	5.857	7.783	13.6	24.2	100.00	9.93	69.91	50.26	49.74	1.000E-03
Third Elution	5.843	7.747	13.6	24.6	100.00	10.08	70.87	50.08	49.92	1.003E-03
k1 with error										9.99±0.04 E-04
20 °C column										
First Elution	5.594	7.264	11.3	21.2	100.00	17.81	73.81	49.73	50.27	1.771E-03
Second Elution	5.583	7.250	12.6	21.4	100.00	18.95	79.30	49.76	50.24	1.718E-03
Third Elution	5.584	7.245	12.2	21.6	100.00	19.09	78.69	49.69	50.31	1.759E-03
k1 with error										1.749±0.03 E-03
25 °C column										
First Elution	5.456	6.976	11.3	19.2	100.00	37.66	83.76	49.33	50.67	3.078E-03
Second Elution	5.431	6.952	13.6	19.8	100.00	42.16	91.89	49.44	50.56	3.017E-03
Third Elution	5.449	6.959	11.4	19.3	100.00	38.42	83.40	49.20	50.80	3.091E-03
k1 with error										3.062±0.04 E-03
30 °C column										
First Elution	5.339	6.698	10.7	18.0	100.00	80.52	90.90	48.73	51.27	5.421E-03

Second Elution	5.343	6.706	12.0	18.5	100.00	88.01	95.32	48.71	51.29	5.518E-03
Third Elution	5.338	6.685	10.8	18.1	100.00	85.67	93.55	48.50	51.50	5.622E-03
k1 with error										5.520±0.10 E-03

Through the Eyring equation, the Gibbs free energy of racemization can be calculated at 25 °C directly from the obtained elution profile:

$$\Delta G^{\ddagger}_{298} = RT\left[ln\left(\frac{k_B T}{h}\right) - ln(k_e)\right] \quad (1)$$

$$= 8.31 \cdot 10^{-3} \cdot 298\left[ln\left(\frac{1.38 \cdot 10^{-23} \cdot 298}{6.63 \cdot 10^{-34}}\right) - ln(3.06 \cdot 10^{-3})\right] = 87.28\ kJ\ mol^{-1}$$

Plotting $ln(k_{eT}/T)$ against $1/T$ for all obtained elution profiles and linear regression of the obtained graph ($y = ax + b$) gives access to the full thermodynamic analysis.

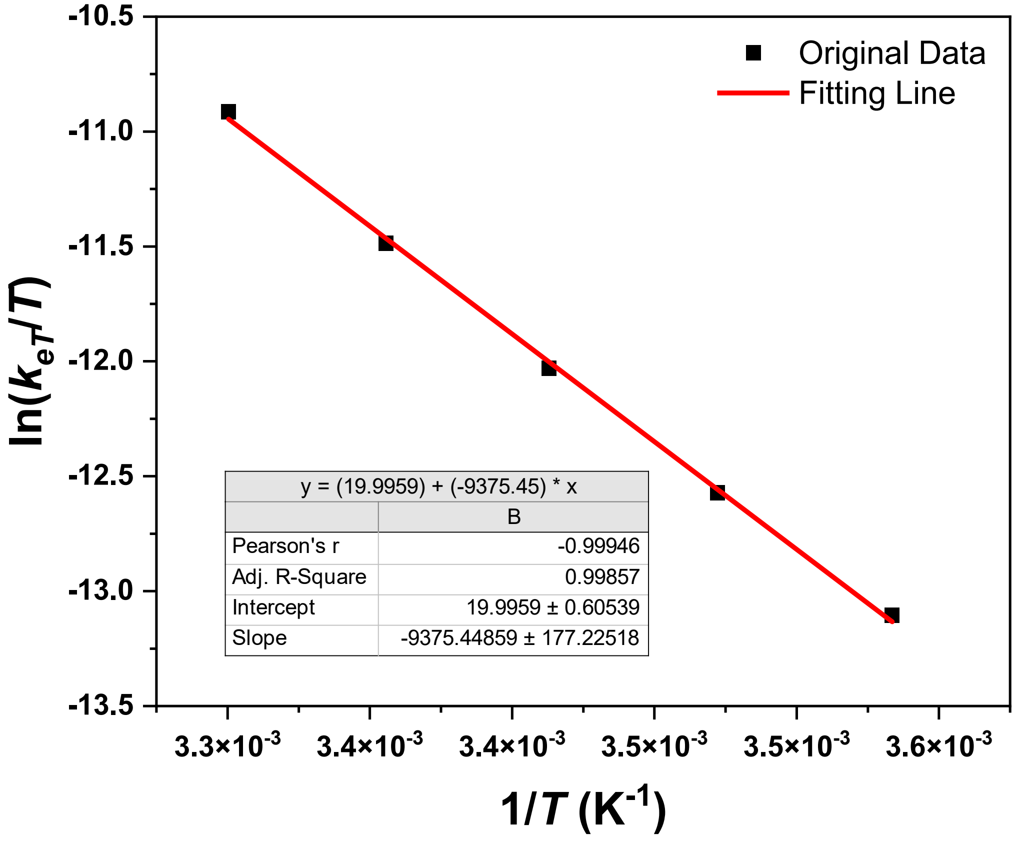

Figure S41. Eyring plot of $\ln(k_{eT}/T)$ against $1/T$ with linear regression ($y=ax+b$).

From this, the entropy and enthalpy of the enantiomerization process can be calculated through:

$$\Delta H_e^{\ddagger} = -aR \tag{2}$$

$$\Delta H_e^{\ddagger} = -(-9375 \cdot 8.31 \cdot 10^{-3}) = 77.91 \pm 1.47 \text{ kJ mol}^{-1}$$

$$\Delta S_e^{\ddagger} = R\left[b - ln\left(\frac{k_B}{h}\right)\right] \tag{3}$$

$$\Delta S_e^{\ddagger} = 8.31 \cdot (19.9959 - 23.8) = -31.61 \pm 5.03 \text{ J mol}^{-1}\text{K}^{-1}$$

From the obtained values, the Gibbs free activation energy can be calculated for every temperature. To validate the data, the activation energy at 25 °C (298 K) is again calculated from the data obtained through the full thermodynamical analysis.

$$\Delta G_{e298}^{\ddagger} = \Delta H_e^{\ddagger} - T\Delta S_e^{\ddagger} \tag{4}$$

$$\Delta G^{\ddagger}_{e298} = 77.91 - (-0.031 \cdot 298) = 87.14 \text{ kJ mol}^{-1}$$

The value obtained directly from $k_{e298\,\mathrm{K}}$ is in good agreement with the value obtained from the full thermodynamical analysis with $\Delta\Delta G^{\ddagger}_{e298}$ = 0.14 kJ mol^{-1}

The enantiomerization barrier of DBP[8]CPP was obtained by dynamic chromatography at 35 °C, using the same separation conditions. The elution profile (Figure S42) was analyzed with the DCXplorer software, which gave an enantiomerization reaction rate of $k_e = 1.406\text{x}10^{-4}$ s^{-1}. Because dynamic HPLC of DBP[8]CPP requires elevated temperatures, and considering solvent and instrument limitations, we only performed the measurement only at 35 °C and used this temperature to estimate the activation Gibbs free energy.

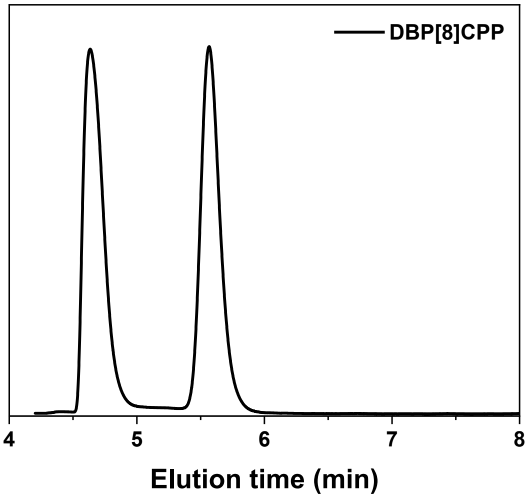

Figure S42. HPLC chromatogram of DBP[8]CPP at 35 °C column temperature

Through the Eyring equation, the Gibbs free energy of racemization can be calculated at 35 °C directly from the obtained elution profile:

$$\Delta G^{\ddagger}_{298} = RT\left[ln\left(\frac{k_B T}{h}\right) - ln(k_e)\right] \quad (1)$$

$$= 8.31 \cdot 10^{-3} \cdot 308\left[ln\left(\frac{1.38 \cdot 10^{-23} \cdot 308}{6.63 \cdot 10^{-34}}\right) - ln(1.406 \cdot 10^{-4})\right] = 98.18 \text{ kJ mol}^{-1}$$

7 Supramolecular Chemistry

7.1 MS Spectra of Nanohoop-Fullerene Complexes

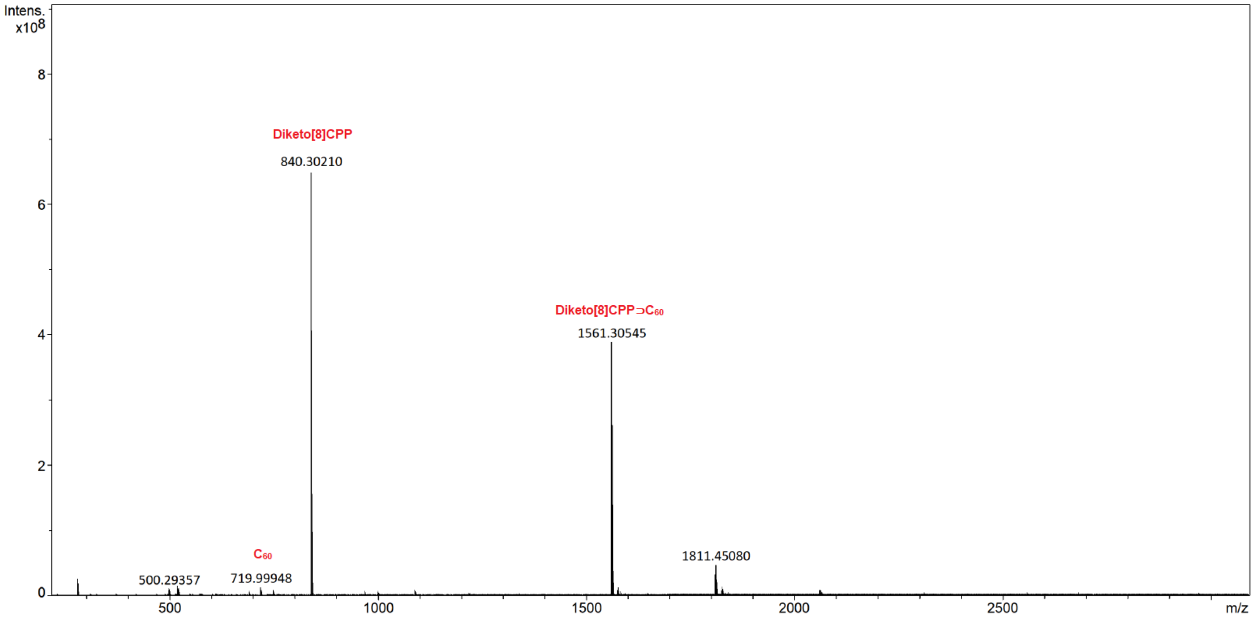

Figure S43: MALDI (positive mode) MS spectrum of diketo[8]CPP with excess C_{60}.

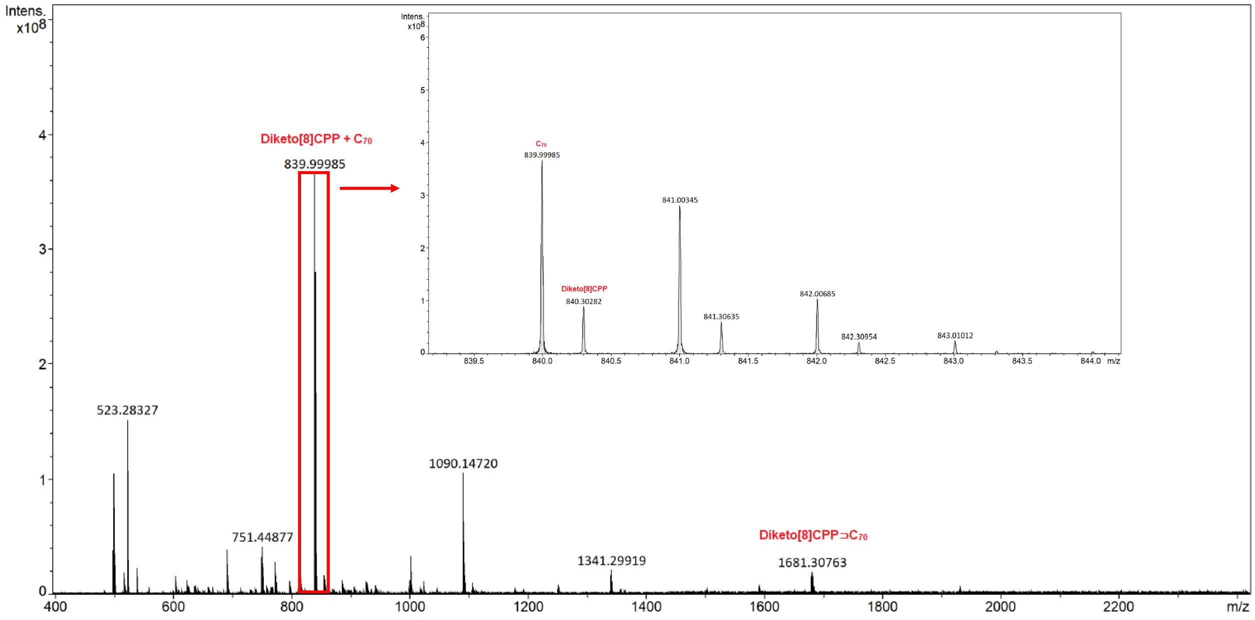

Figure S44: MALDI (positive mode) MS spectrum of diketo[8]CPP with excess C_{70}.

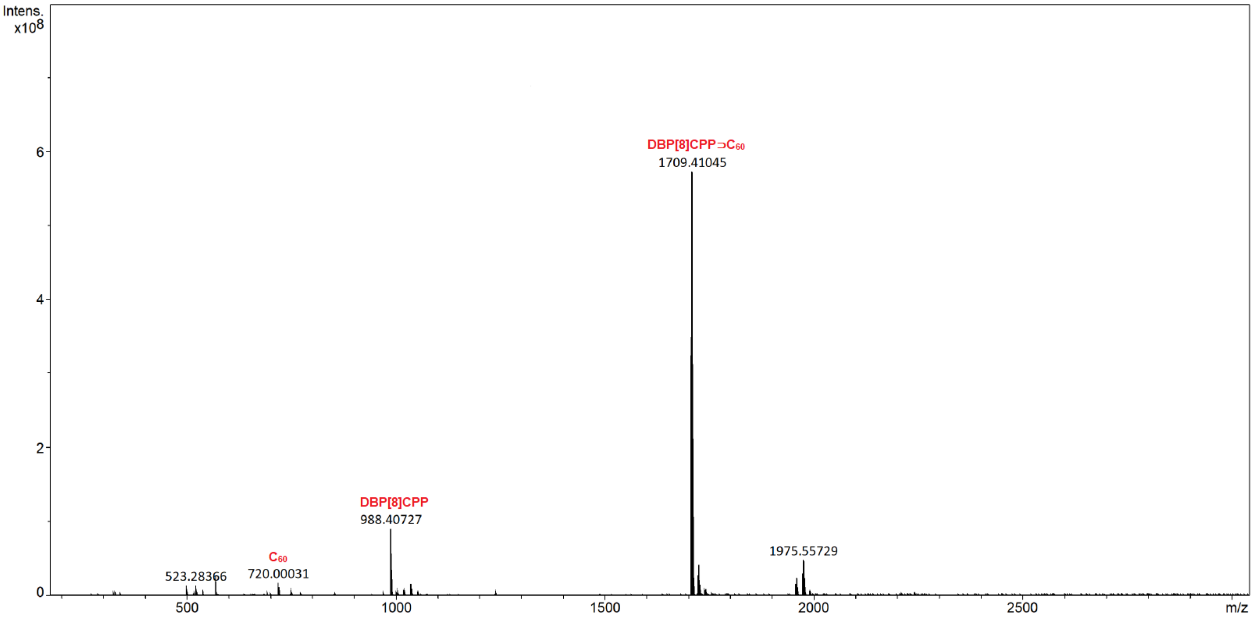

Figure S45: MALDI (positive mode) MS spectrum of DBP[8]CPP with excess C_{60}.

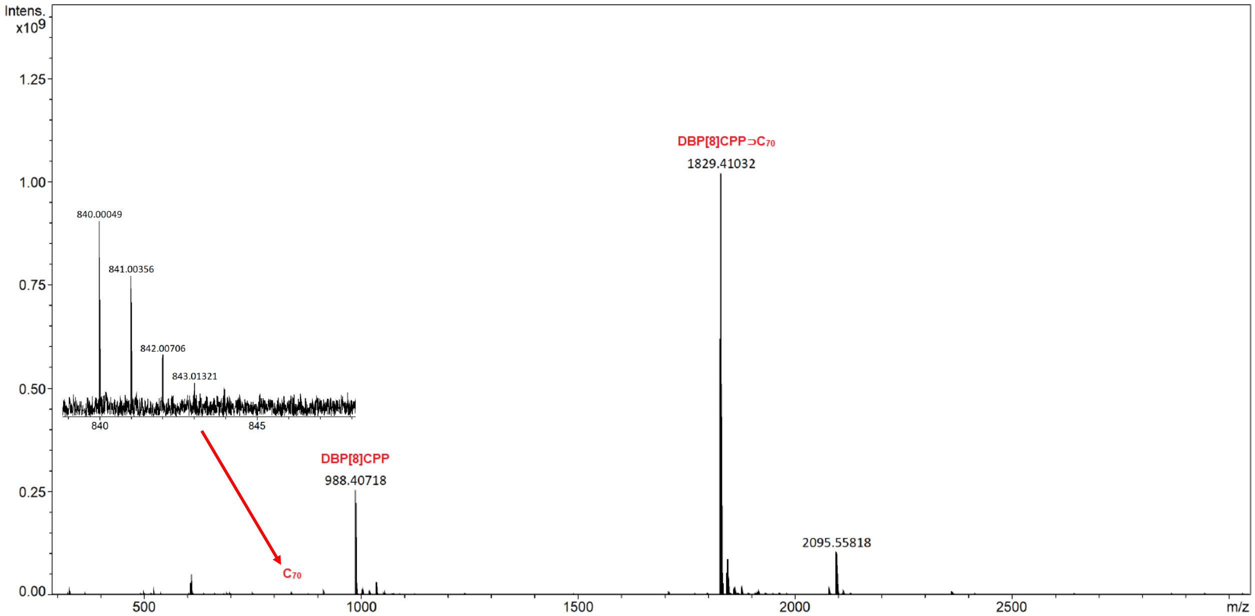

Figure S46: MALDI (positive mode) MS spectrum of DBP[8]CPP with excess C_{70}.

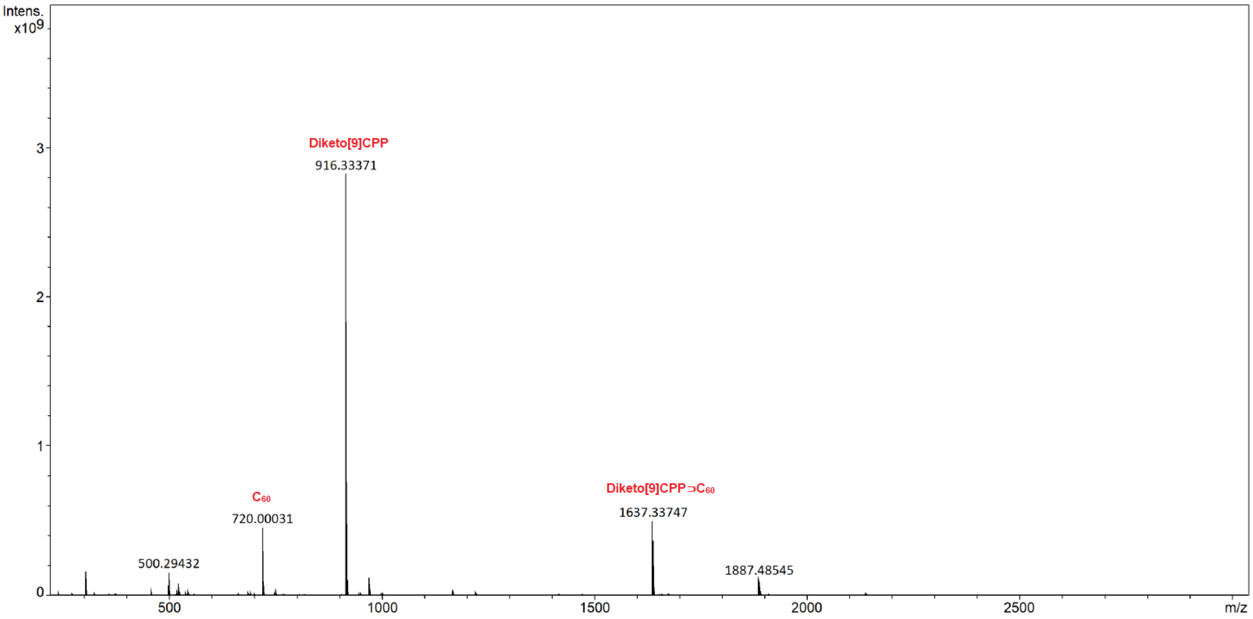

Figure S47: MALDI (positive mode) MS spectrum of diketo[9]CPP with excess C_{60}.

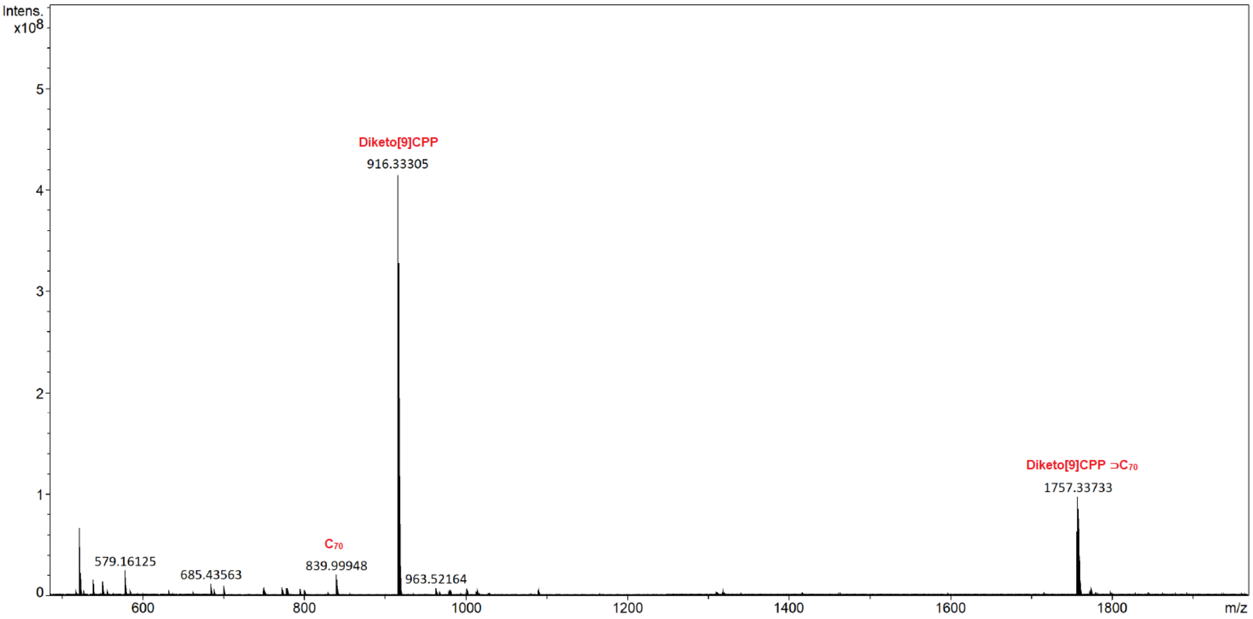

Figure S48: MALDI (positive mode) MS spectrum of diketo[9]CPP with excess C_{70}.

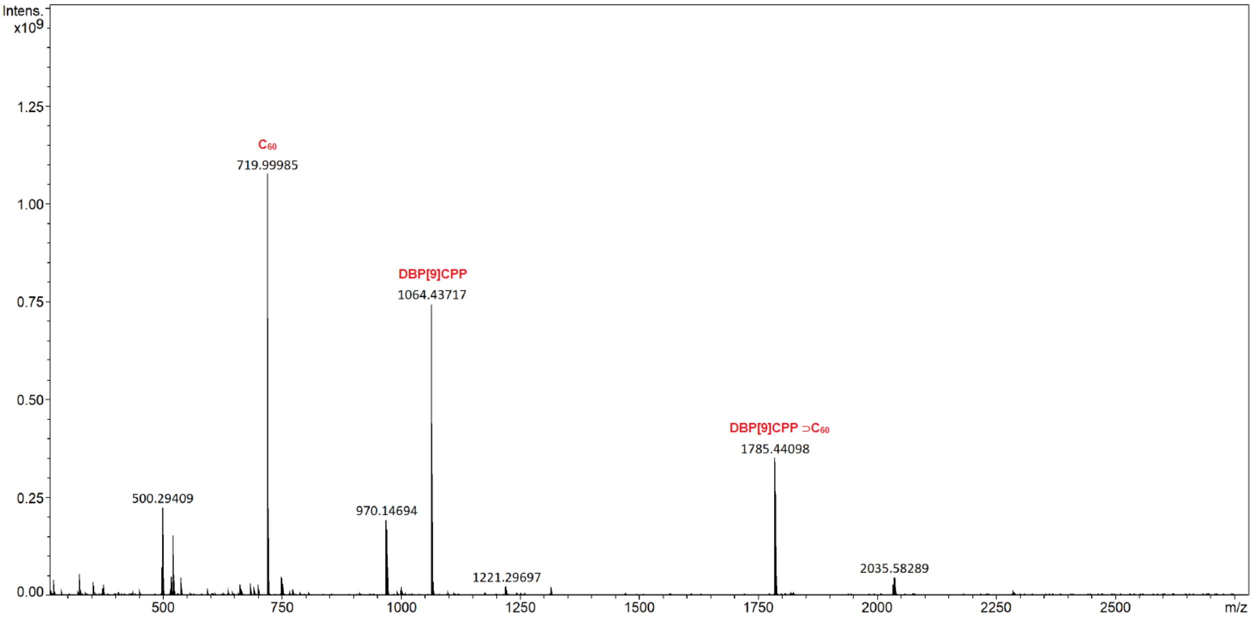

Figure S49: MALDI (positive mode) MS spectrum of DBP[9]CPP with excess C_{60}.

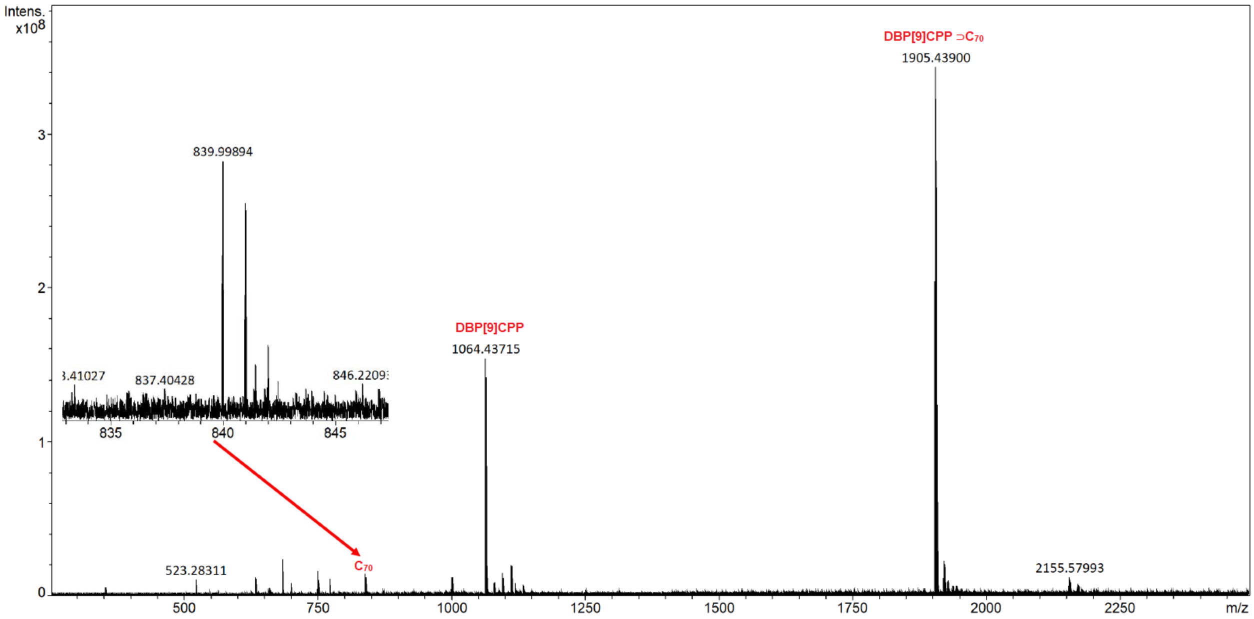

Figure S50: MALDI (positive mode) MS spectrum of DBP[9]CPP with excess C_{60}.

Sample preparation: Stock solutions of diketo[9]CPP and DBP[9]CPP were prepared in CH_2Cl_2 (0.2 g l^{-1}) while the stock solutions of C_{60} and C_{70} were prepared in toluene (0.5 g l^{-1}). The ESI experiments were carried out with the analyte solutions consisting of equimolar amounts of host and guest solution at a concentration of 1×10^{-5} mol L^{-1}. For the ESI experiments with fullerene complexes a solvent mixture of MeCN/CH_2Cl_2 /toluene (3:2:1, v:v:v) was used. To both a small amount of trifluoroacetic acid was added to enhance ionization.

Breakdown graphs: Breakdown graphs are a plot of the survival yield (SY) as a function of the collision energy. The SY is the ratio of precursor ions surviving the collision event at a given collision energy against the total amount of ions observed. The collision energy is given as the laboratory energy (E_{lab}) divided by the degrees of freedom (DoF) of the hosting nanohoop. This approach of defining the collision energy is based on previous studies.[6] The number of DoF was calculated according to Eqn. 5:

$$DoF = (3 * n) - 6 \quad (5)$$

With *n* corresponding to the number of atoms of the host.

All SY curves were recorded under multiple collision conditions and fitted with a sigmoid Boltzmann function. The collision energy, E_{50}, at which 50% of the parent ions have dissociated into their fragment ions is chosen as a relative measure of stability. The fragmentation energies obtained represent relative rather than absolute values with respect to the actual energy demand of the dissociation processes.

Diketo[9]CPP + $C_{60/70}$

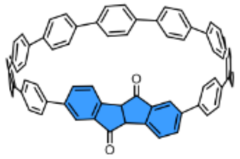

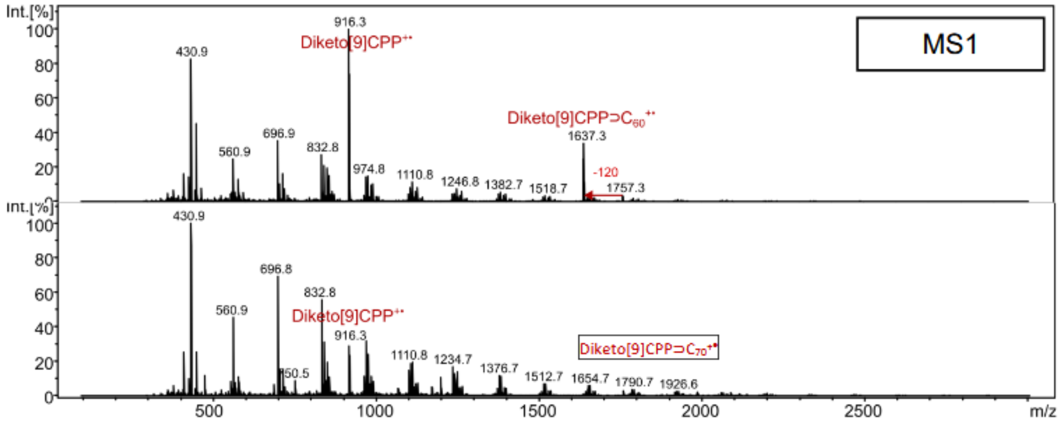

Figure S51. ESI mass spectra (MS[1]) of diketo[9]CPP with C_{60} and C_{70}.

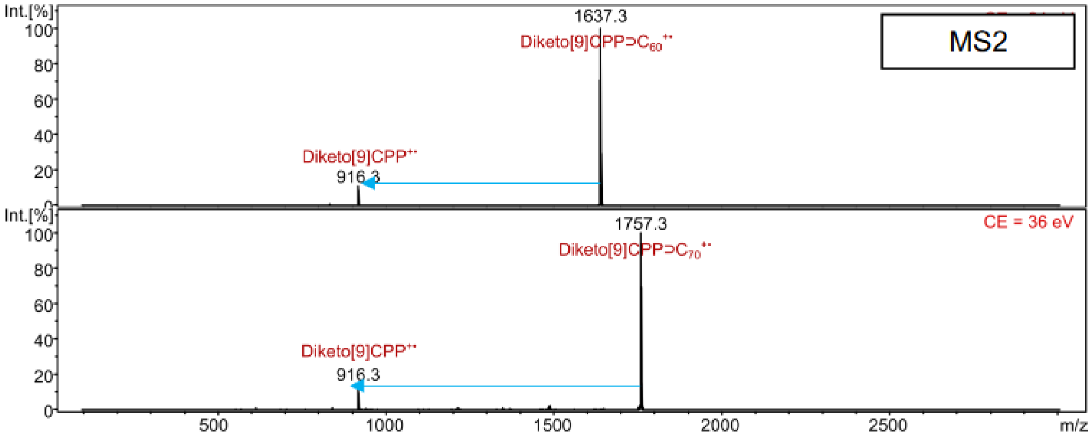

Figure S52. Collision-induced dissociation (MS[2]) mass spectra of diketo[9]CPP$^{•+}$⊃C_{60} and diketo[9]CPP$^{•+}$⊃C_{70}.

DBP[9]CPP + $C_{60/70}$

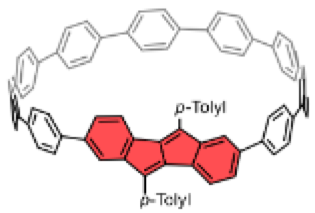

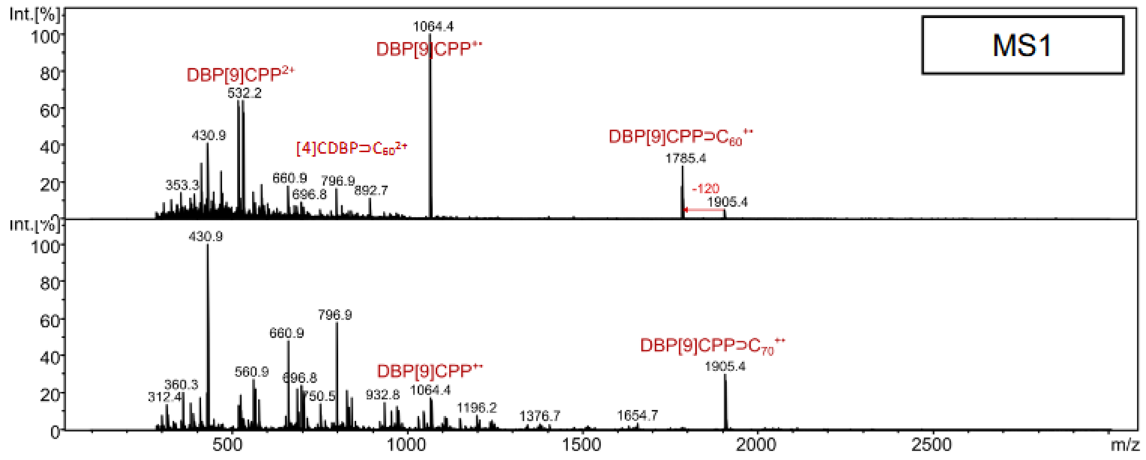

Figure S53. ESI mass spectra (MS^1) of DBP[9]CPP with C_{60} and C_{70}.

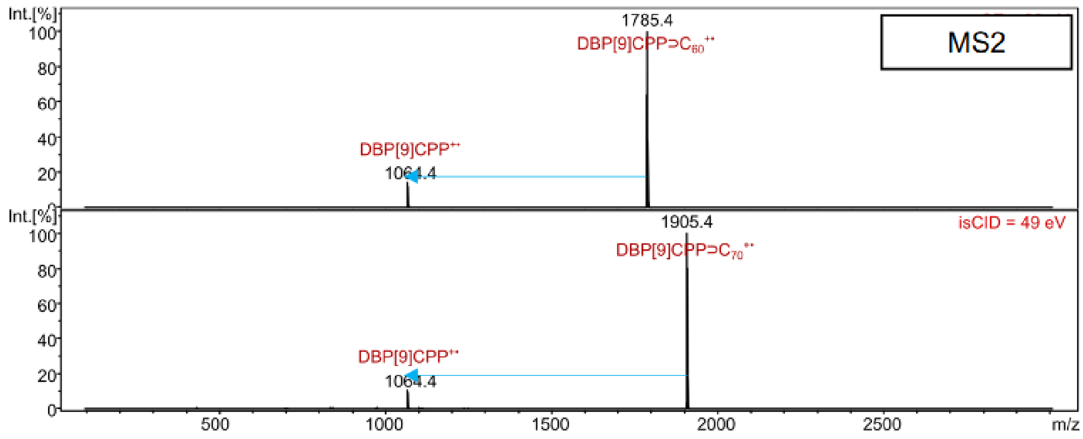

Figure S54. Collision-induced dissociation (MS^2) mass spectra of DBP[9]$CPP^{•+}$⊃C_{60} and DBP[9]$CPP^{•+}$⊃C_{70}.

7.2 NMR Studies of Nanohoop-Fullerene Complexation

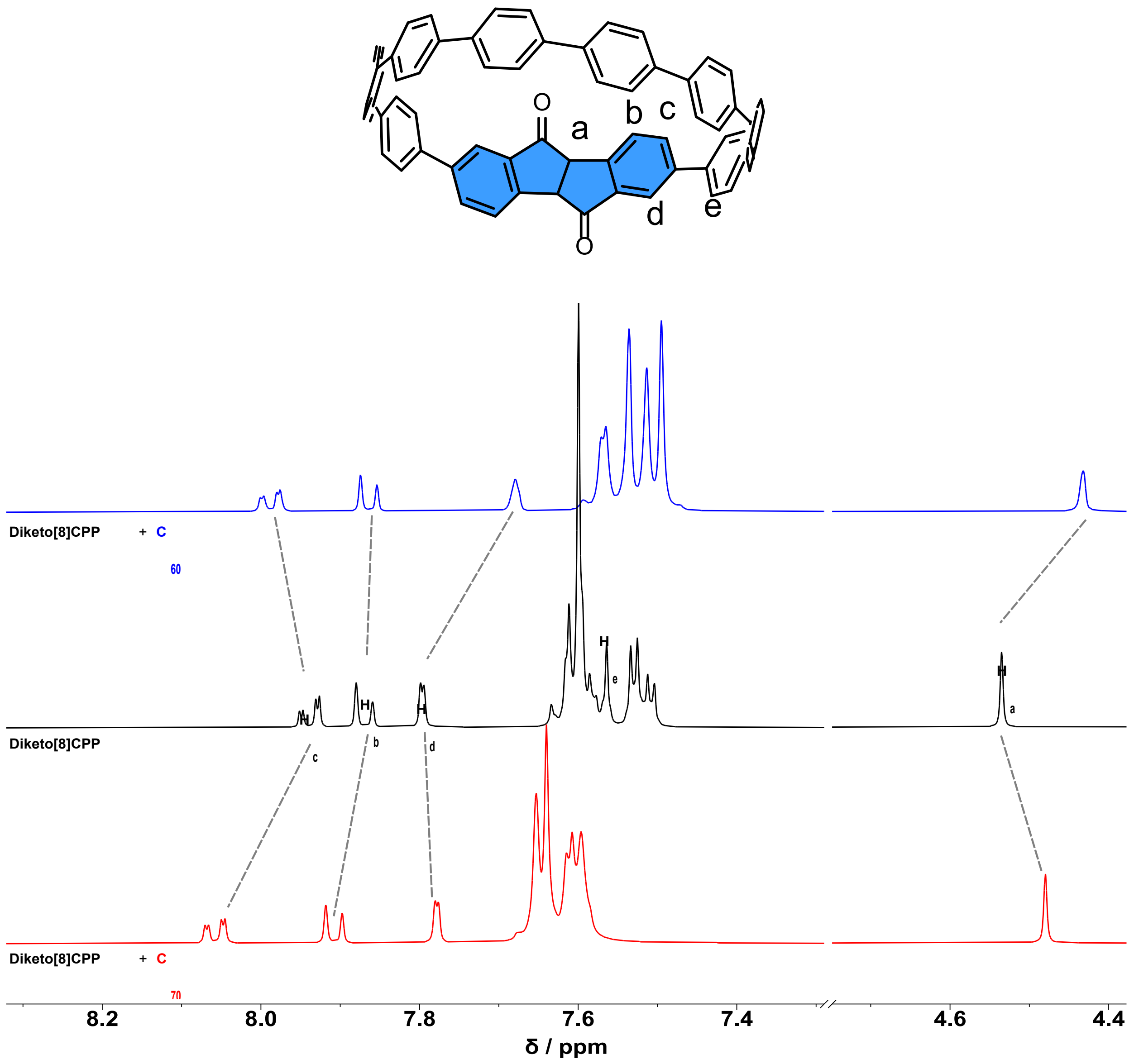

Figure S55. Section of the ^{1}H NMR spectra (1,1,2,2-tetrachloroethane-d_2, 400 MHz) of diketo[8]CPP by itself (center) and in the presence of 3 eq. C_{60} (top) or C_{70} (bottom).

Discussion: Upon addition of C_{60} or C_{70} to diketo[8]CPP, H_a shows a noticeable upfield. In contrast, the aromatic protons (H_b, H_c, H_d, H_e) exhibit a mixture of upfield and downfield movements. The observed chemical-shift variations arise from a combination of the guest's intrinsic magnetic-anisotropy pattern and host structural responses upon complexation.

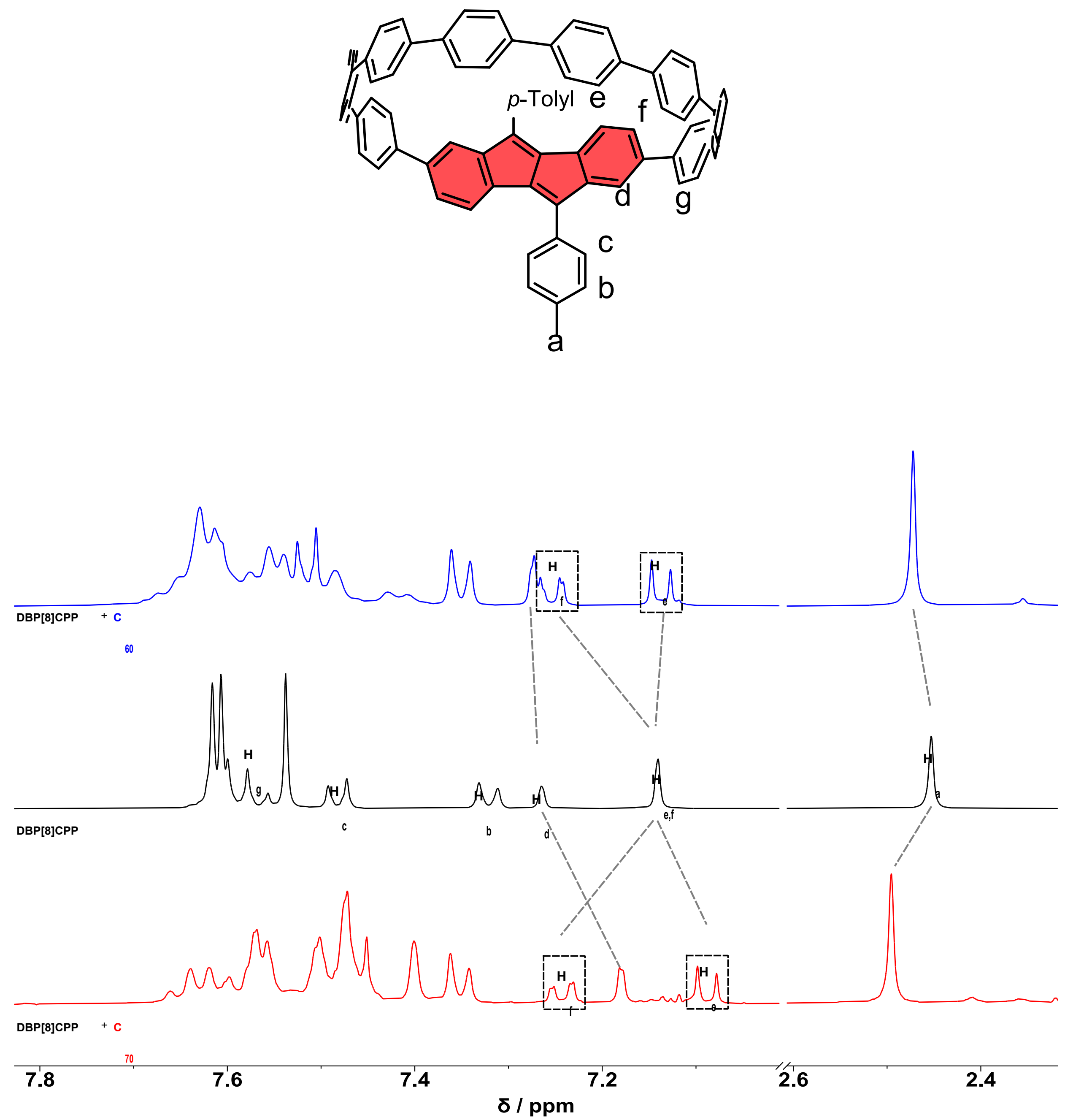

Figure S56. Section of the ^{1}H NMR spectra (1,1,2,2-tetrachloroethane-d_2, 400 MHz) of DBP[8]CPP by itself (center) and in the presence of 3 eq. C_{60} (top) or C_{70} (bottom).

Discussion: For DBP[8]CPP, the addition of C_{60} or C_{70} leads to chemical-shift changes. The observed upfield and downfield movements arise mainly from the magnetic-anisotropy pattern of the fullerene guests. As shown in the ^{1}H NMR spectra of the DBP[n]CPPs (Figures S15 and S20), the introduction of the fullerene guest leads to the splitting of the previously overlapping H_e and H_f resonances, thereby enabling a clear and reliable structural assignment.

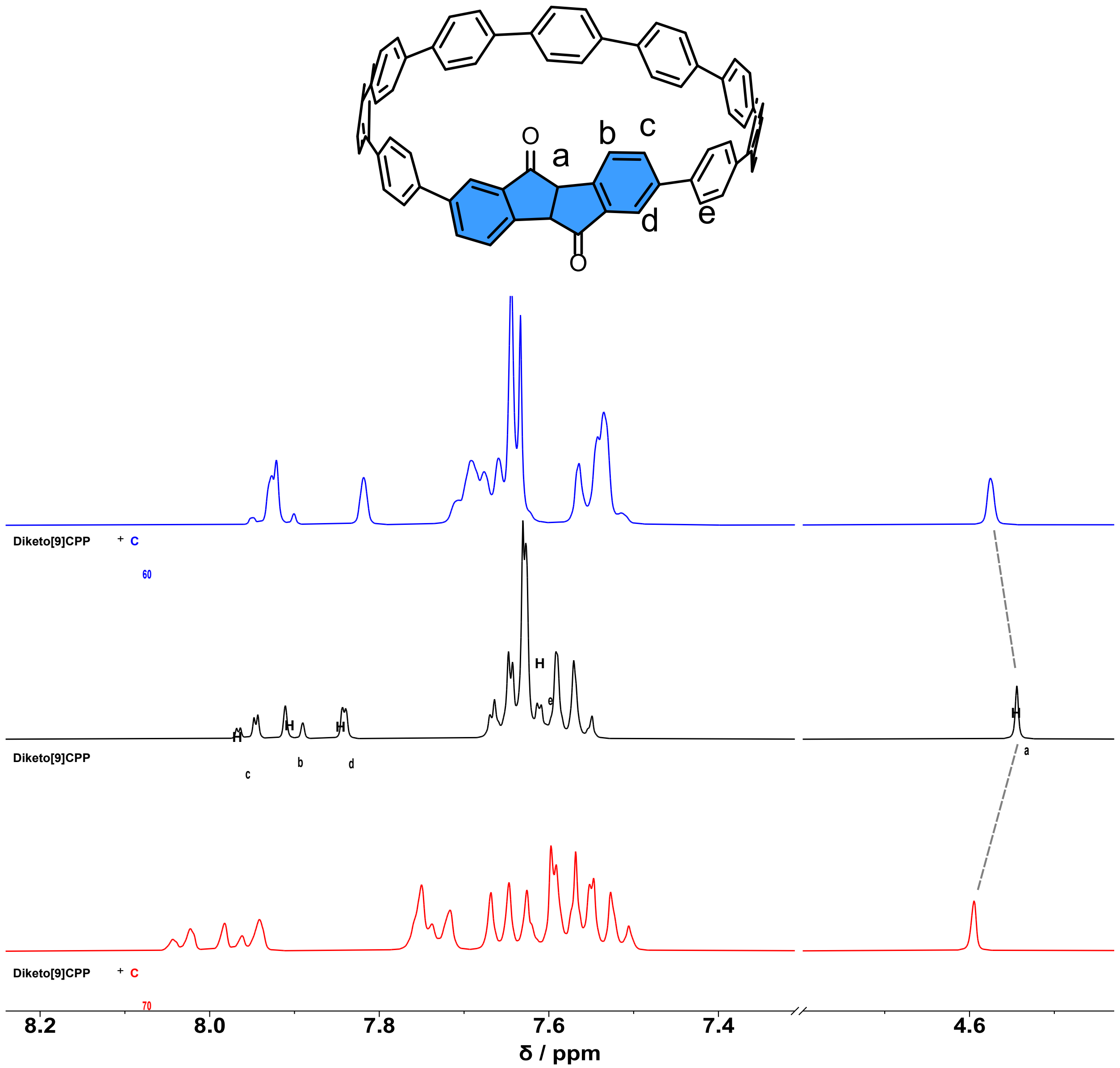

Figure S57. Section of the ^{1}H NMR spectra (1,1,2,2-tetrachloroethane-d_2, 400 MHz) of diketo[9]CPP by itself (center) and in the presence of 3 eq. C_{60} (top) or C_{70} (bottom).

Discussion: Upon addition of C_{60} or C_{70} to diketo[9]CPP, the protons exhibit a mixture of upfield and downfield movements. These changes mainly come from the guest's intrinsic magnetic-anisotropy pattern, while theoretical calculations and the single-crystal structure show that the host undergoes only very small structural changes upon complexation.

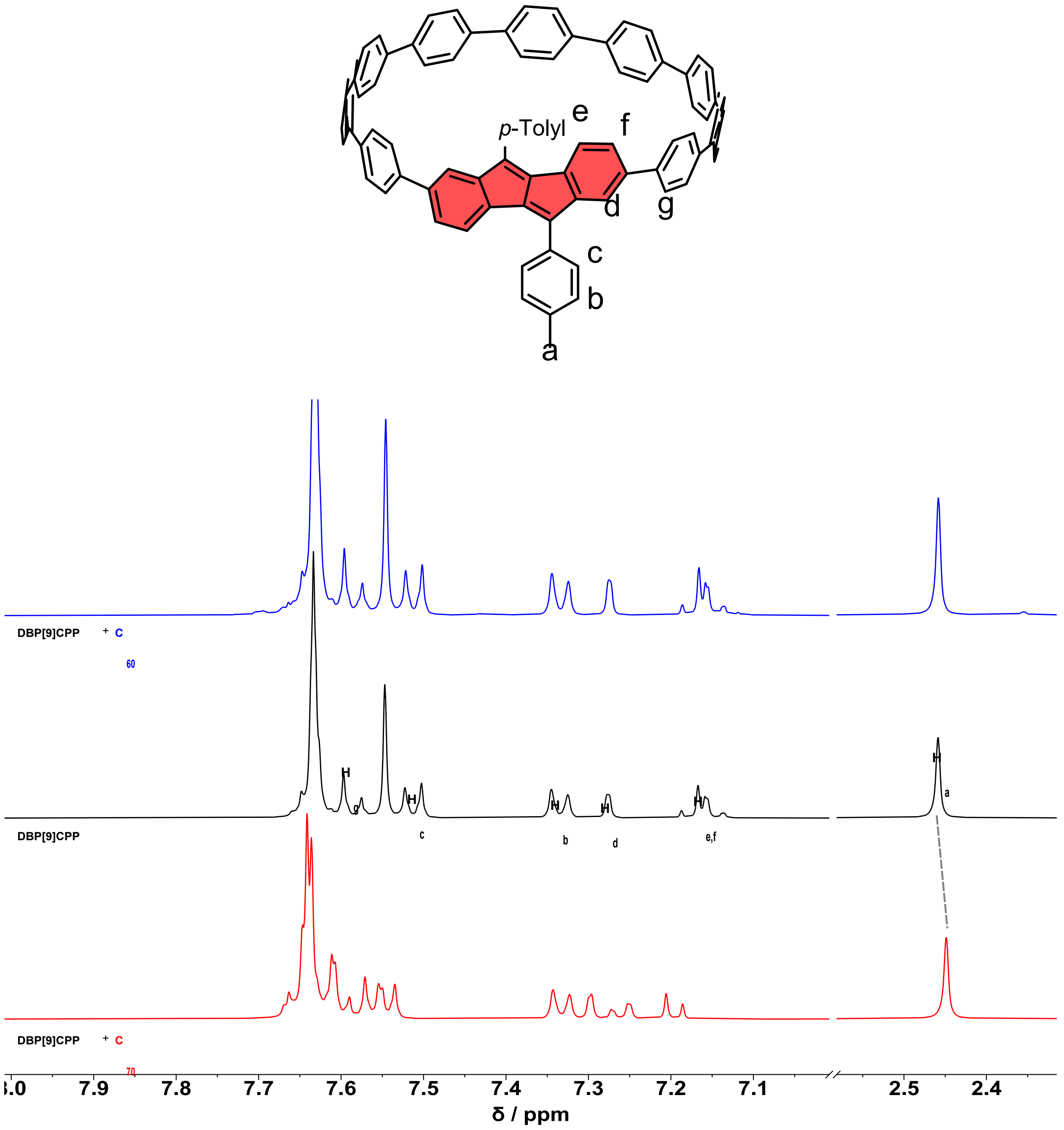

Figure S58. Section of the ^{1}H NMR spectra (1,1,2,2-tetrachloroethane-d_2, 400 MHz) of DBP[9]CPP by itself (center) and in the presence of 3 eq. C_{60} (top) or C_{70} (bottom).

Discussion: For DBP[9]CPP, almost no chemical-shift changes are observed upon addition of C_{60}, which can be due to the large size of the nanohoop. By contrast, C_{70} produces small but measurable shifts.

7.3 Fluorescence Titration Experiments

A solution of diketo[n]CPPs (3.00 mL) was titrated with fullerene stock solutions (C_{60}: 8.19×10^{-4} M, C_{70}: 3.21×10^{-4} M, see corresponding graphs for exact amount of the respective guest) in toluene at 25 °C. In each parallel titration, the total volumes of added fullerene stock solutions were less than 3% of the combined volumes. Accordingly, the concentration of diketo[n]CPPs was regarded as constant during the titration process. The final concentration of fullerene varied depending on the titration range and host concentration used. The resulting solutions were measured by fluorescence spectroscopy. The resulting titration curves were analyzed using the BindFit program (http://supramolecular.org)[7, 8], which performs nonlinear least-squares regression analysis. A 1:1 host–guest binding model was applied. Each titration experiment was repeated three times to ensure reproducibility.

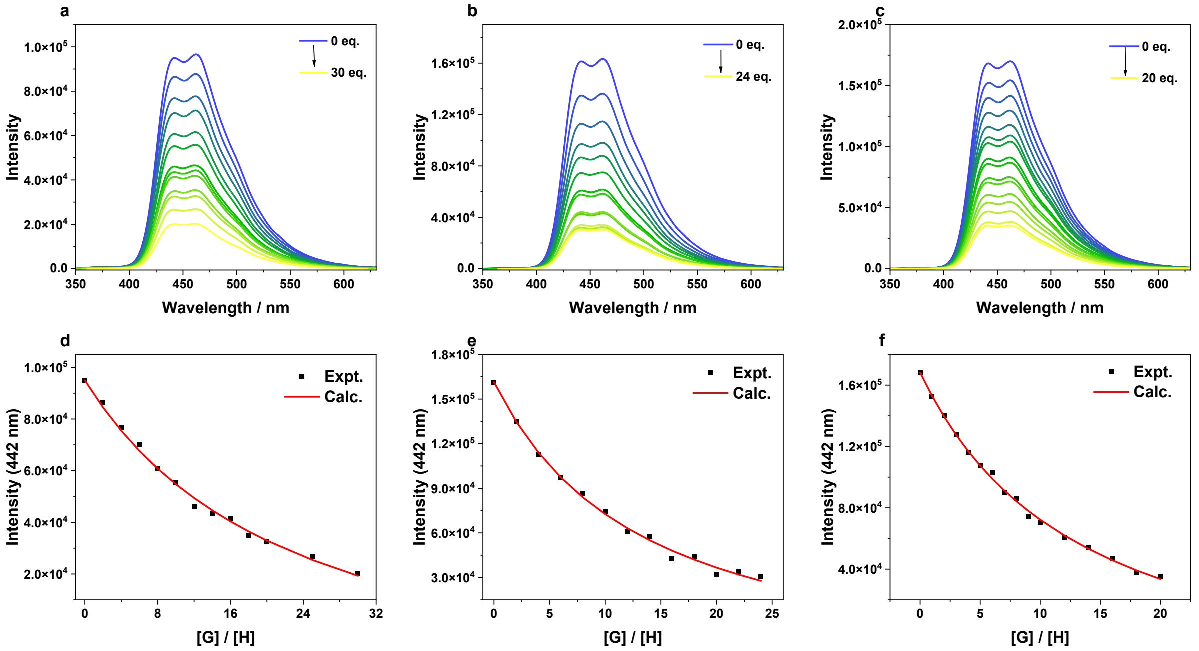

Figure S59. (a–c) Fluorescence spectra of diketo[8]CPP (a) 8×10^{-7} M, b)–c) 1.2×10^{-6} M; λ_{ex} = 330 nm) during titration with C_{60} in toluene. (d–f) Fluorescence titration plot for determination of the binding constant of diketo[8]CPP to C_{60} (d) 8×10^{-7} M, e)–f) 1.2×10^{-6} M) in the presence of various amounts of the mixture of C_{70} (1:1).

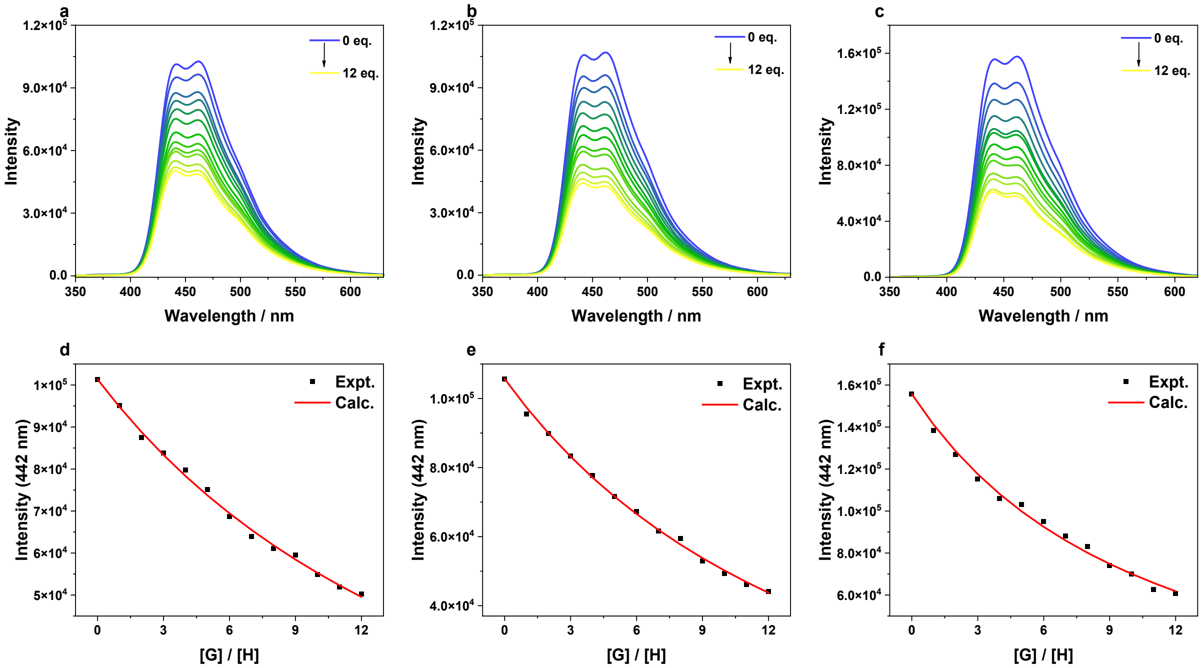

Figure S60. (a–c) Fluorescence spectra of diketo[8]CPP (a) 8 × 10^{-7} M, b) 1 × 10^{-6} M, c) 1.2 × 10^{-6} M; λ_{ex} = 330 nm)) during titration with C_{70} in toluene. (d–f) Fluorescence titration plot for determination of the binding constant of diketo[8]CPP (d) 8 × 10^{-7} M, e) 1 × 10^{-6} M, f) 1.2 × 10^{-6} M) in the presence of various amounts of the mixture of C_{70} (1:1).

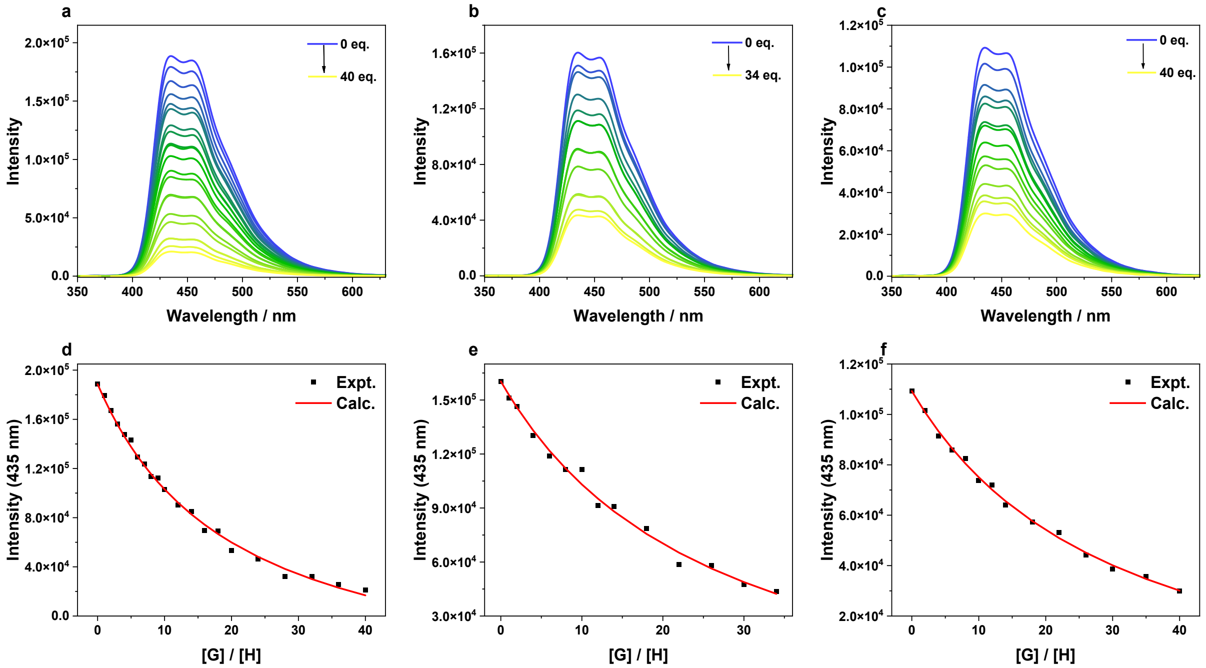

Figure S61. (a–c) Fluorescence spectra of diketo[9]CPP (a) 1 × 10^{-6} M, b) 8 × 10^{-7} M, c) 6 × 10^{-7} M; λ_{ex} = 330 nm) during titration with C_{60} in Toluene. (d–f) Fluorescence titration plot for determination of the binding constant of diketo[9]CPP (d) 1 × 10^{-6} M, e) 8 × 10^{-7} M, f) 6 × 10^{-7} M) in the presence of various amounts of the mixture of C_{60} (1:1).

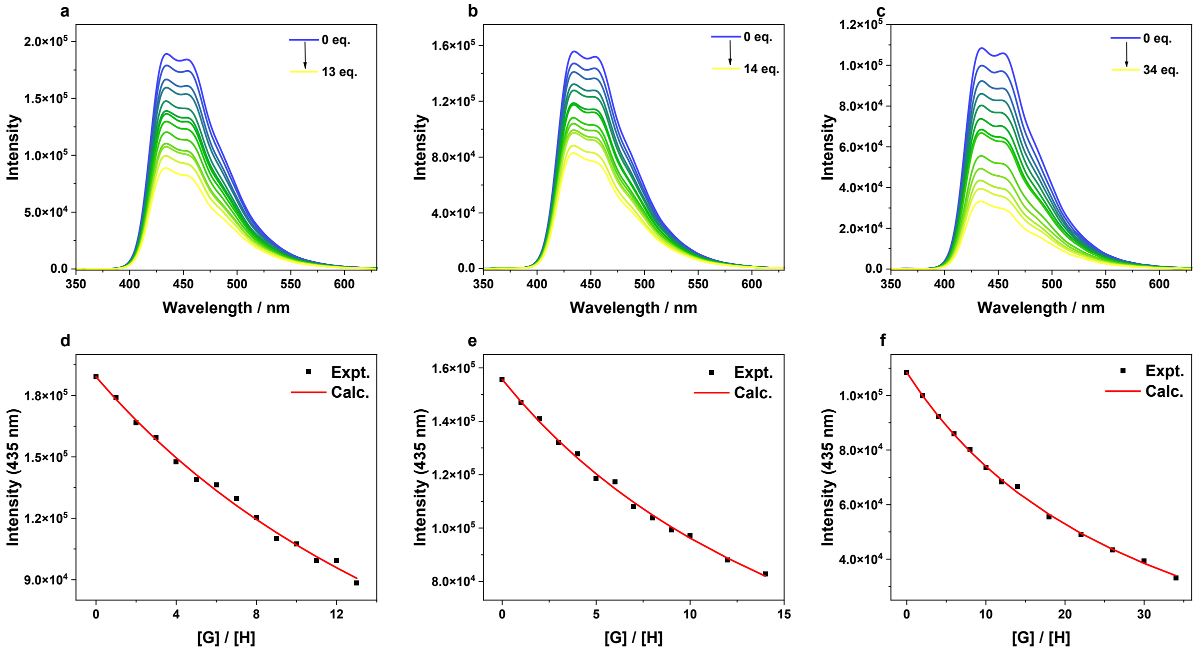

Figure S62. (a–c) Fluorescence spectra of diketo[9]CPP (a) 1×10^{-6} M, b) 8×10^{-7} M, c) 6×10^{-7} M; $\lambda_{ex} = 330$ nm) during titration with C_{70} in toluene. (d–f) Fluorescence titration plot for determination of the binding constant of diketo[9]CPP (d) 1×10^{-6} M, e) 8×10^{-7} M, f) 6×10^{-7} M) in the presence of various amounts of the mixture of C_{70} (1:1).

Table S2. Experimental binding constants K [M^{-1}] of 1:1 host–guest complexes from fluorescence titrations of diketo[*n*]CPPs with C_{60} and C_{70} in toluene.

Experiment		1	2	3	average
Diketo[8]CPP	C_{60}	5.55(±0.14) ×10^4	6.61(±0.17) ×10^4	6.65(±0.10) ×10^4	6.27(±0.14) ×10^4
	C_{70}	6.73(±0.12) ×10^4	6.49(±0.09) ×10^4	8.24(±0.22) ×10^4	7.15(±0.14) ×10^4
Diketo[9]CPP	C_{60}	5.20(±0.11) ×10^4	4.84(±0.18) ×10^4	5.55(±0.11) ×10^4	5.20(±0.13) ×10^4
	C_{70}	4.21(±0.08) ×10^4	6.36(±0.11) ×10^4	5.42(±0.07) ×10^4	5.33(±0.09) ×10^4

8. Single Crystal X-Ray Diffraction

8.1 General Experimental Setup and Solution

X-ray diffraction data of single crystals for diketo[9]CPP, DBP[9]CPP and diketo[9]CPP⊃C_{60} were collected on a Rigaku/Oxford SuperNova single crystal four-circle diffractometer equipped with a low temperature device, Atlas CCD detector and used Cu*Kα* radiation (λ = 1.54184 Å) with a micro-focus sealed X-ray tube. Data were collected from a shock-cooled single crystal at 150(2) K.

X-ray diffraction data of single crystals for diketo[8]CPP were collected at the P24 beamline of the PETRA III at DESY (Hamburg, Germany) equipped with a four-circle HUBER four-circle diffractometer with Eulerian geometry, an X Spectrum LAMBDA 7.5M pixel array detector with a pixel size of 55×55 µm, and an openflow nitrogen cryosystem at T = 105 K. The data were acquired by 360° rotation φ-scans with 0.1° scan width with 2θ = 17° and κ = −45° and an exposure time of 1 s per frame at the wavelength λ = 0.56 Å and a detector distance of 150 mm. All data were handled (importing, indexing, integration, scaling, absorption correction) using CrysAlisPro 1.171.43.66a (Rigaku OD 2024) and a multi-scan absorption correction using SCALE3 ABSPACK was applied.

All data were integrated with CrysalisPro 43.112a and a multi-scan absorption correction using SCALE3 ABSPACK was applied.[9] The structures were solved by direct methods using SHELXT and refined by full-matrix least-squares methods against F^2 by SHELXL-2019/1.[10, 11] All non-hydrogen atoms were refined with anisotropic displacement parameters. All hydrogen atoms were refined isotropically on calculated positions using a riding model with their U_{iso} values constrained to 1.5 times the U_{eq} of their pivot atoms for terminal sp^3 carbon atoms and 1.2 times for all other carbon atoms. Some disordered solvent problems were solved by the program PLATON/SQUEEZE (see details for each structure).[12] Some disordered moieties were refined using bond length restraints and displacement parameter restraints. Some parts of the disorder model were introduced by the program DSR.[13] Crystallographic data for the structures reported in this paper have been deposited with the Cambridge Crystallographic Data Centre.[14] These data can be obtained free of charge from The Cambridge Crystallographic Data Centre via www.ccdc.cam.ac.uk/structures. The CIF files were generated using FinalCif (v139).[15] Mercury was used for structural representations.

8.2 Details for Each Crystal

Diketo[9]CPP

A suitable yellow block shaped crystal was obtained by solvent diffusion of MeCN into a THF solution. The MeCN molecules could be placed on discrete positions and restrained using DSR. The THF solvent molecules present inside the hoop cavities could not be modeled discretely and therefore the electron density was described using PLATON/SQUEEZE. The electron count amounts to 8 THF molecules in total.

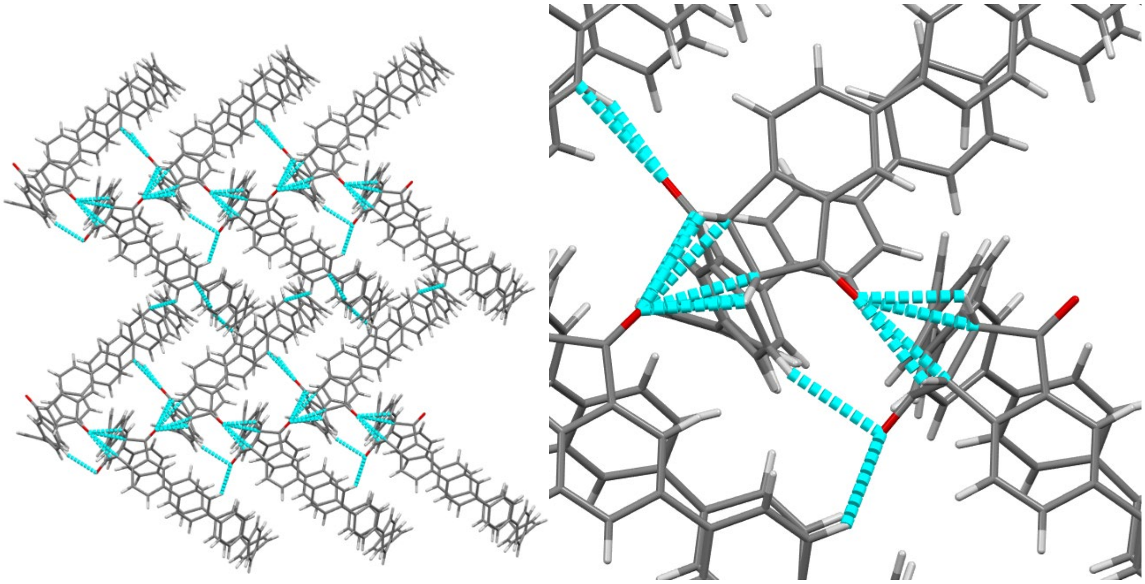

View of the noncovalent interactions (dotted blue lines) between the adjacent molecules. Intermolecular distances. Contacts length: 2.5–3.2 Å.

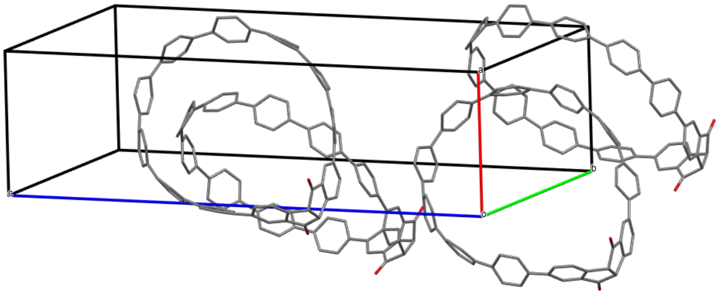

DBP[9]CPP

A suitable clear dark brown block-shaped crystal was obtained by solvent diffusion of *n*-hexane into $CHCl_3$ solution. The unit cell contains 14 *n*-hexane molecules (3.5 in the asymmetric unit) which have been treated as a diffuse contribution to the overall scattering without specific atom positions by PLATON/SQUEEZE. The "unsqueezed" structure contains 1.5 n-hexane molecules outside of the hoop and 2 molecules inside the hoop. The volume and electron count matches roughly with these 3.5 molecules in the asymmetric unit (14 in the cell). The solvent molecules were included in the molecular formula for calculation of the intensive properties.

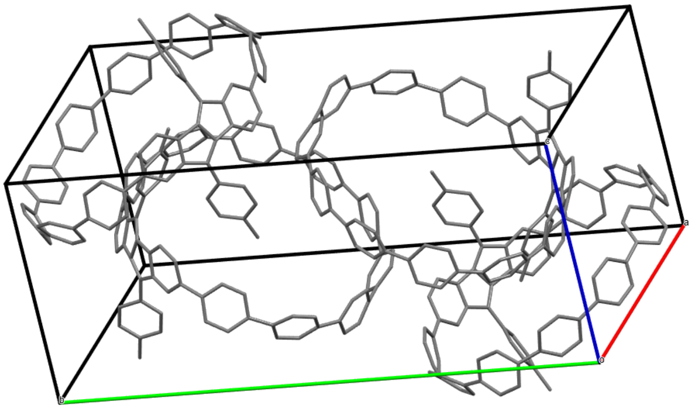

Diketo[8]CPP

A suitable yellow, needle shaped crystal was obtained by solvent diffusion of MeCN into a dichloroethane solution. The structure featured a very long cell length in the *a* direction. The small crystal size resulted in a weak diffraction up to only 1.05 Å, even using synchrotron radiation. We recorded data for several crystals, but this reported dataset yielded the best result. The solvent molecules inside the cavity are not well ordered, leading to a diminished quality. The flexibility in their position lead to high displacement parameters. One para-phenyl group was disordered on two positions by rotation. Additionally, there were indications, that other phenyl groups might be disordered over two positions as well (as it is often observed for this type of nanohoop) but since the data quality was already not the best, and the positional disorder was not as pronounced, we chose not to model this disorder.

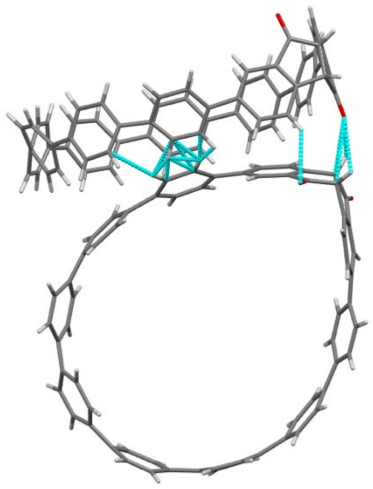

View of the noncovalent interactions (dotted blue lines) between the adjacent molecules. Intermolecular distances. Contacts length: 2.1–3.2 Å.

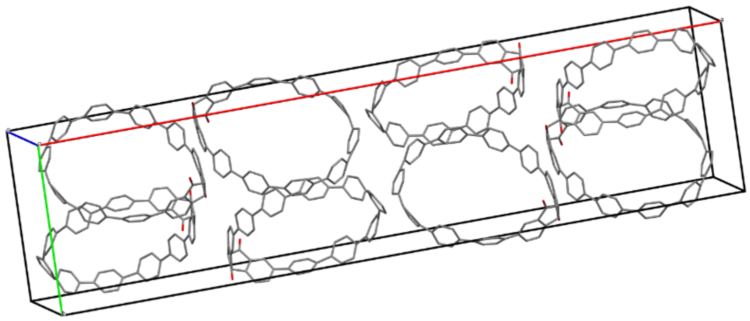

Diketo[9]CPP⊃C_{60}

A suitable crystal was obtained by vapor diffusion of MeCN into a 1,2-dichlorobenzene solution. A clear dark red, block-shaped crystal was mounted on a MiTeGen micromount with perfluoroether oil. The structure contains 4.5 1,2-dichlorobenzene solvent molecules in the asymmetric unit and 18 molecules in the cell. These were heavily disordered and were therefore masked using PLATON SQUEEZE. A single MeCN molecule is present and was modelled. The fullerene inside the cavity was restrained and placed on two positions using DSR. The top half of the diketoneCPP structure is disordered over two positions consisting of both enantiomers of the diketone structure with a ratio of 57 / 43. Distance and displacement parameter restraints were used.

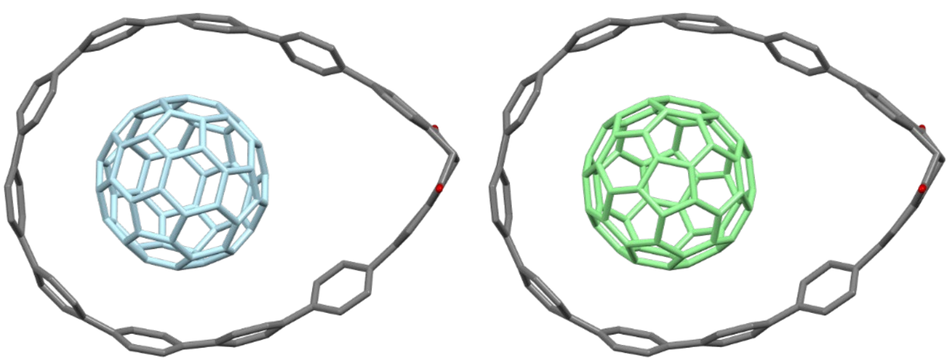

Crystal structure of Diketo[9]CPP⊃C_{60} and its disorders. Two different orientations of C_{60} were shown in different colors.

Table S3. Structural parameters.

Compound	**Diketo[9]CPP**	**DBP[9]CPP**	**Diketo[8]CPP**	**Diketo[9]CPP⊃C60**
CCDC number	2364437	2365575	2427456	2486379
Empirical formula	$C_{72}H_{47}NO_2$	$C_{84}H_{563.5}(C_6H_{14})$	$C_{72}H_{54}Cl_4N_2O_2$	$C_{132}H_{47}NO_2$
Formula weight	958.10	1366.88	1120.97	1678.70
Temperature [K]	150.00(14)	150(2)	105(2)	150(2)
Crystal system	monoclinic	monoclinic	monoclinic	monoclinic
Space group (number)	Pc (7)	$P2_1/c$ (14)	$C2/c$ (15)	$P2_1/n$ (14)
a [Å]	10.3442(3)	18.8736(5)	68.988(4)	33.9339(7)
b [Å]	17.7628(6)	31.9978(8)	16.9569(10)	9.7569(2)
c [Å]	37.0847(10)	14.3224(4)	10.6338(11)	34.8054(8)
α [°]	90	90	90	90
β [°]	91.107(3)	96.305(3)	93.366(6)	106.600(2)
γ [°]	90	90	90	90
Volume [Å^3]	6812.8(3)	8597.2(4)	12418.1(17)	11043.4(4)
Z	4	4	8	4
ρ_{calc} [gcm^{-3}]	0.934	1.056	1.199	1.010
μ [mm^{-1}]	0.428	0.442	0.128	0.459
F(000)	2008	2940	4672	3448
Crystal size [mm^3]	0.412×0.230×0.186	0.350×0.210×0.150	0.02×0.02×0.05	0.130×0.170×0.360
Crystal colour	yellow	clear dark brown	yellow	clear dark red
Crystal shape	block	block	needle	block
Radiation	CuK_α (λ=1.54184 Å)	CuK_α (λ=1.54184 Å)	synchrotron (λ=0.560 Å)	CuK_α (λ=1.54184 Å)
2θ range [°]	5.52 to 145.85 (0.81 Å)	4.71 to 137.13 (0.83 Å)	3.77 to 30.93 (1.05 Å)	7.63 to 149.01 (0.80 Å)
Index ranges	$-12 \leq h \leq 12$<br>$-21 \leq k \leq 17$<br>$-45 \leq l \leq 45$	$-21 \leq h \leq 22$<br>$-38 \leq k \leq 38$<br>$-17 \leq l \leq 14$	$-65 \leq h \leq 65$<br>$-16 \leq k \leq 16$<br>$-10 \leq l \leq 9$	$-40 \leq h \leq 42$<br>$-12 \leq k \leq 9$<br>$-43 \leq l \leq 39$
Reflections collected	129080	90093	18226	95597
Independent reflections	26757<br>R_{int} = 0.0937<br>R_{sigma} = 0.0516	15787<br>R_{int} = 0.1762<br>R_{sigma} = 0.1054	5576<br>R_{int} = 0.1239<br>R_{sigma} = 0.0921	22510<br>R_{int} = 0.1006<br>R_{sigma} = 0.0639
Completeness to θ =	100.0 %<br>66.4915°	100.0 %<br>67.684°	99.4 %<br>15.465°	99.9 %
Data / Restraints / Parameters	26757/2339/1353	15787/0/759	5576/1376/780	22510/50393/2125
Absorption correction T_{min}/T_{max} (method)	0.39971/1.00000 (multi-scan)	0.32793/1.00000 (multi-scan)	0.6201/1.0000 (multi-scan)	0.5283/1.0000 (multi-scan)
Goodness-of-fit on F^2	1.032	0.946	1.060	1.593
Final R indexes [I≥2σ(I)]	R_1 = 0.1177<br>wR_2 = 0.2966	R_1 = 0.0704<br>wR_2 = 0.1788	R_1 = 0.2189<br>wR_2 = 0.5321	R_1 = 0.1425<br>wR_2 = 0.4572
Final R indexes [all data]	R_1 = 0.1730<br>wR_2 = 0.3587	R_1 = 0.1226<br>wR_2 = 0.2219	R_1 = 0.2396<br>wR_2 = 0.5650	R_1 = 0.1567<br>wR_2 = 0.4756
Largest peak/hole [eÅ^{-3}]	0.42/−0.28	0.19/−0.18	1.54/−0.74	1.32/−0.37
Flack X parameter	0.4(3)	-	-	-

9. DFT Calculations

All calculations were performed on the Justus 2 High Performance Computing Cluster (JUSTUS 2).[16] Density functional theory (DFT) calculations were performed with the TURBOMOLE v7.7.1 program package,[17] or the Gaussian 16 Rev.C.01 program package.[18] All structural modifications were done with Avogadro.[19] Visualization and image generation of molecules were done with Avogadro, Chimera[20] or VMD[21]. The methodologies are explained in detail in the respective sub chapter alongside the used software.

Initially the structures of diketo[8]CPP, DBP[8]CPP, diketo[9]CPP and DBP[9]CPP were optimized at the PBEh-3c[22] level of theory without any symmetry restrictions. Stationary points on the potential energy surface were confirmed by vibrational frequency analysis (no imaginary frequency). These structures were further used to calculate frontier molecular orbitals, theoretical UV/Vis and ECD spectra.

Table S4: Calculated single-point energies, zero-point vibrational energies and total energies.

	$E_{\text{PBEh-3c}}$/ Hartrees	E_{PW6B95}/ Hartrees	E(ZVPE) $_{\text{PBEh-3c}}$/ kcal mol^{-1}	E_{total}/ kcal mol^{-1}
Diketo[8]CPP	-2607.5836685	-2618.0237821	544.4937357	-1635738.96266
Diketo[9]CPP	-2838.1299654	-2849.4957343	596.9643836	-1780356.47753
DBP[8]CPP	-2996.9273366	-3008.9896699	677.9938035	-1879922.30299
DBP[9]CPP	-3227.4783976	-3240.4677123	730.3415851	-2024542.93024

9.1 Strain Energies

Strain energies were calculated with the StrainViz[23] scheme developed by JASTI and coworkers. The Gaussian program package was used applying the B3LYP[24,25] density functional and the 6-31G(d)[26] basis set.

Table S5. Calculated fractional and total strain energies of the nanohoops.

Strain[a]	Diketo[8]CPP	Diketo[9]CPP	DBP[8]CPP	DBP[9]CPP
Total	43.8	40.8	59.0	53.1
Bond	4.3	1.5	4.1	3.1
Angle	3.3	3.8	4.4	4.3
Dihedral	36.1	35.4	50.5	45.6

[a] All energies are given in kcal·mol^{-1}

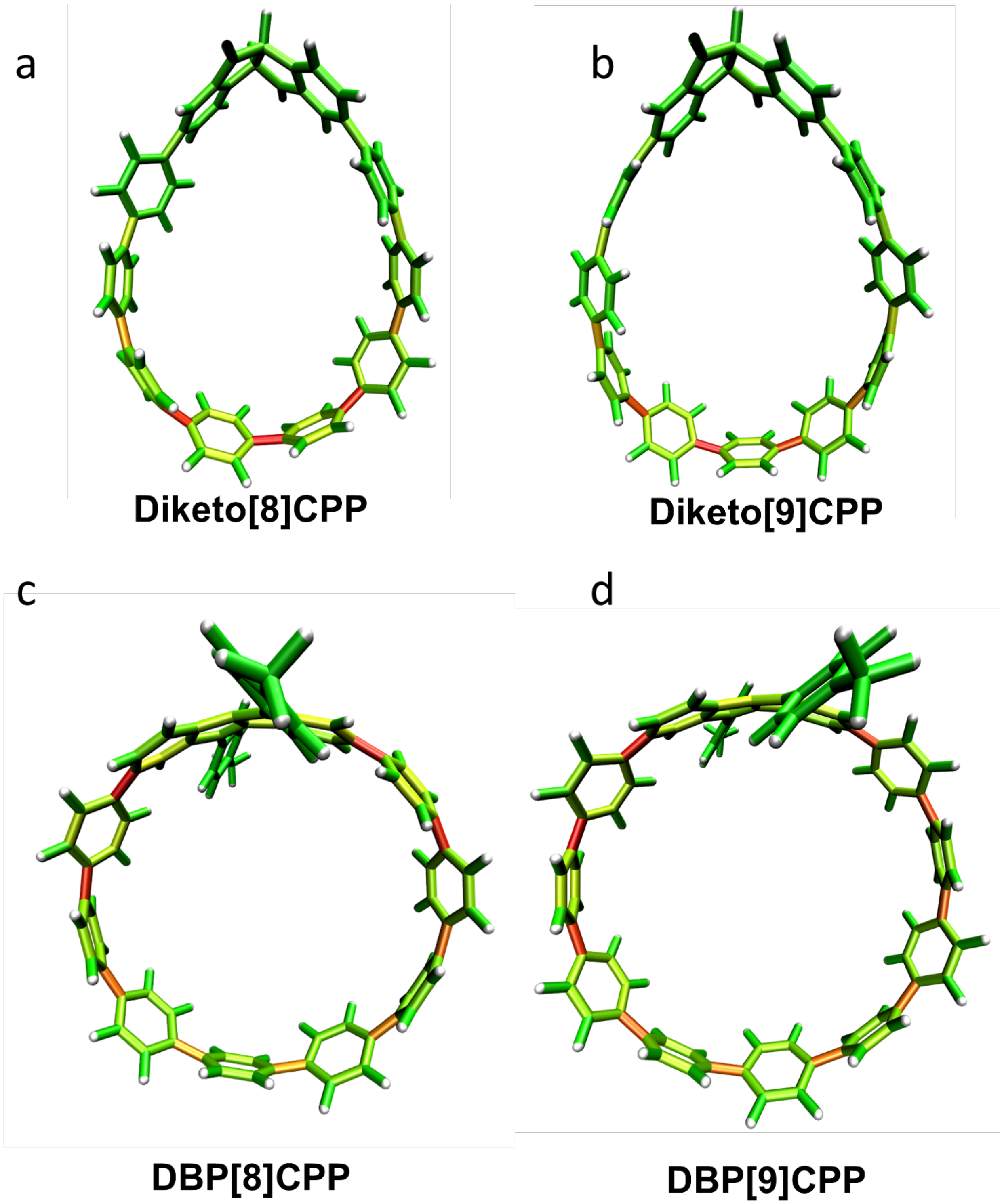

Figure S63. Graphic representations of the calculated localized strain energies.

In the figure below the strain energies of several diketo[*n*]CPPs and DBP[*n*]CPPs hoops are plotted as a function of the respective value n. Additionally, the strain energies for [*n*]CPPs with n = 6–9 are shown. Values for [*n*]CPPs are taken from the literature.[2,23]

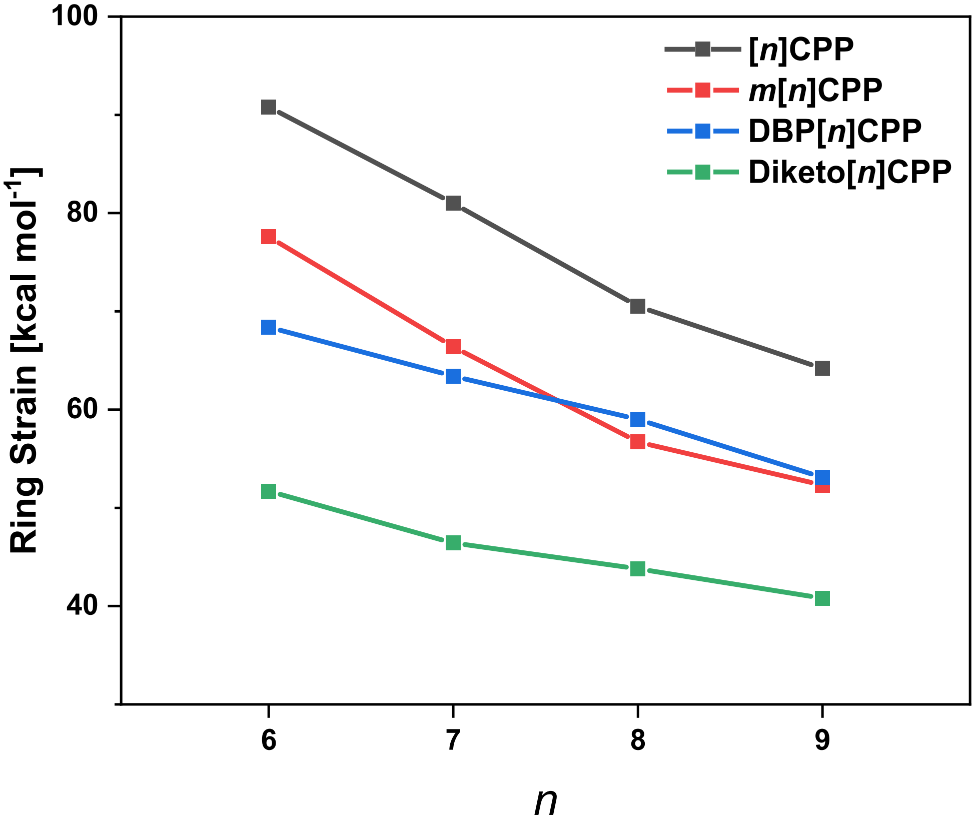

Figure S64. Strain energy in selected diketo[*n*]CPP, DBP[*n*]CPPs, *m*[*n*]CPPs and [*n*]CPPs.

9.2 Calculation of Racemization Barriers

Calculation of transition states were done with the TS option applying the Berny algorithm as implemented in the Gaussian program package. The B3LYP[24,25] functional and the 6-31G(d,p) basis set[26] was used. Transition states were confirmed by the presence of one imaginary frequency.

Table S6. Calculated energies of the ground- and transition states.

	DBP[8]CPP	DBP[9]CPP
E_{TS} [Eh]	-3002.731141	-3233.734263
$ZPVE_{TS}$ [Eh]	1.047345	1.128293
Imag. Frequency [cm^{-1}]	-12.62	-10.99
E_{GS} [Eh]	-3002.766928	-3233.766083
$ZPVE_{GS}$ [Eh]	1.047956	1.129057
ΔE [Eh]	0.035787	0.03182
**ΔE [kcal·mol^{-1}]**	22.5	19.9
**ΔE [kJ·mol^{-1}]**	94.0	83.5

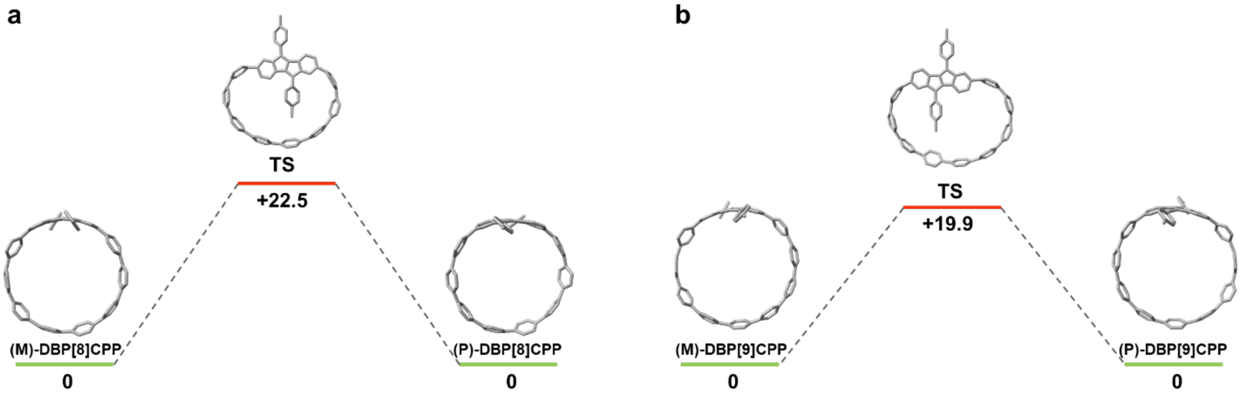

Figure S65. Theoretical isomerization process of (a) DBP[8]CPP and (b) DBP[9]CPP in units of kcal mol^{-1}.

9.3 TD-DFT Calculations

Single point calculations of diketo[8]CPP, DBP[8]CPP, diketo[9]CPP and DBP[9]CPP were conducted with the Turbomole program package on at the PW6B95[27]-D3(BJ)[28,29]/def2-QZVPP[30] level of theory using the PBEh-3c optimized geometries.

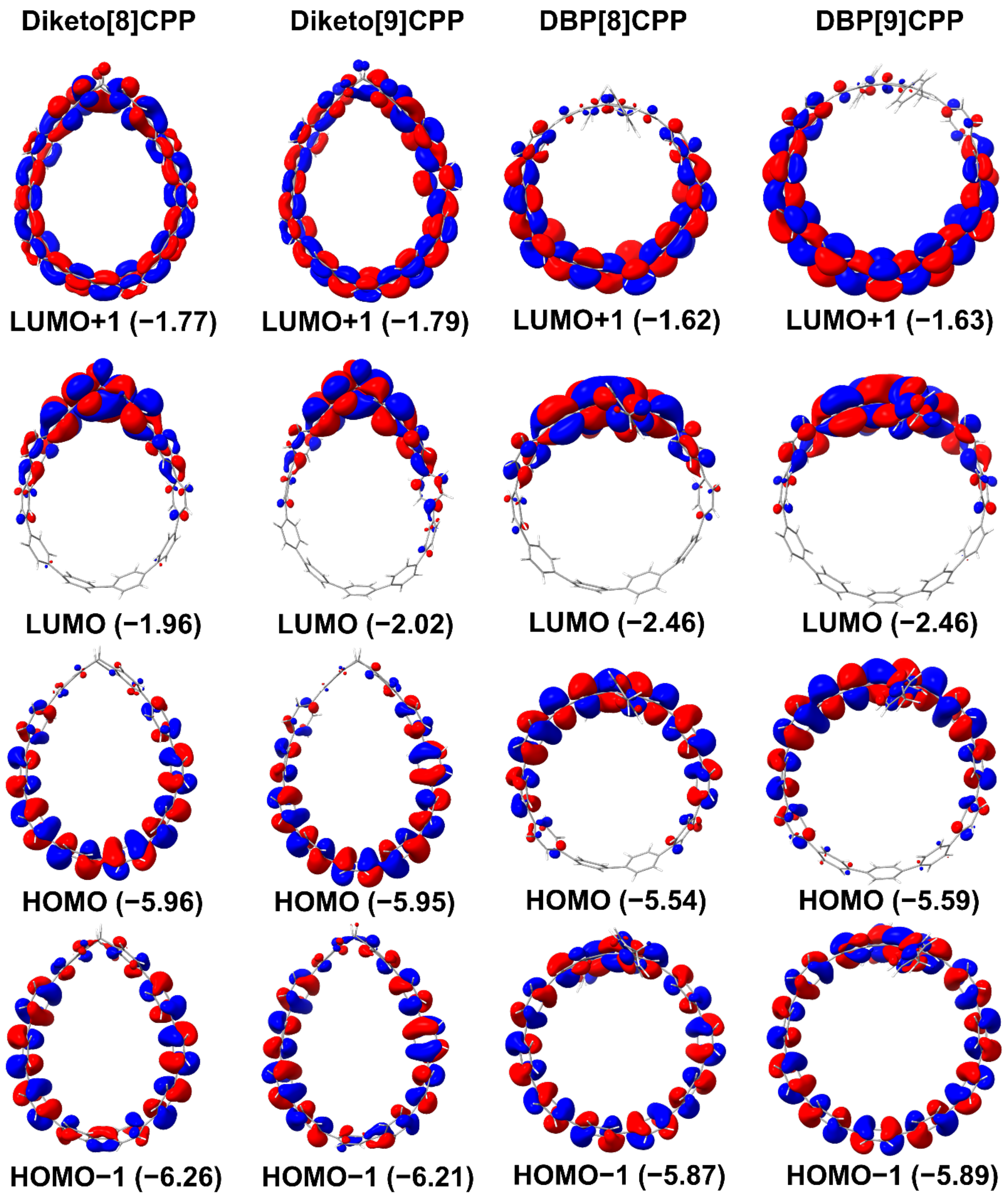

Figure S66. Frontier molecular orbitals (with energies in eV) of Diketo[8]CPP, DBP[8]CPP, Diketo[9]CPP and DBP[9]CPP.

For the assignment of the observed transitions in the absorption spectra we calculated the first 30 singlet excitations for diketo[8]CPP, DBP[8]CPP, diketo[9]CPP and DBP[9]CPP. These TD-DFT calculations were performed within the Turbomole program package on the B3LYP-D3(BJ)/def2-TZVP level of theory using the PBEh-3c optimized geometries. The calculated oscillator strengths were visualized using SpecDis v1.70 applying a gaussian band shape (σ=0.2 eV).

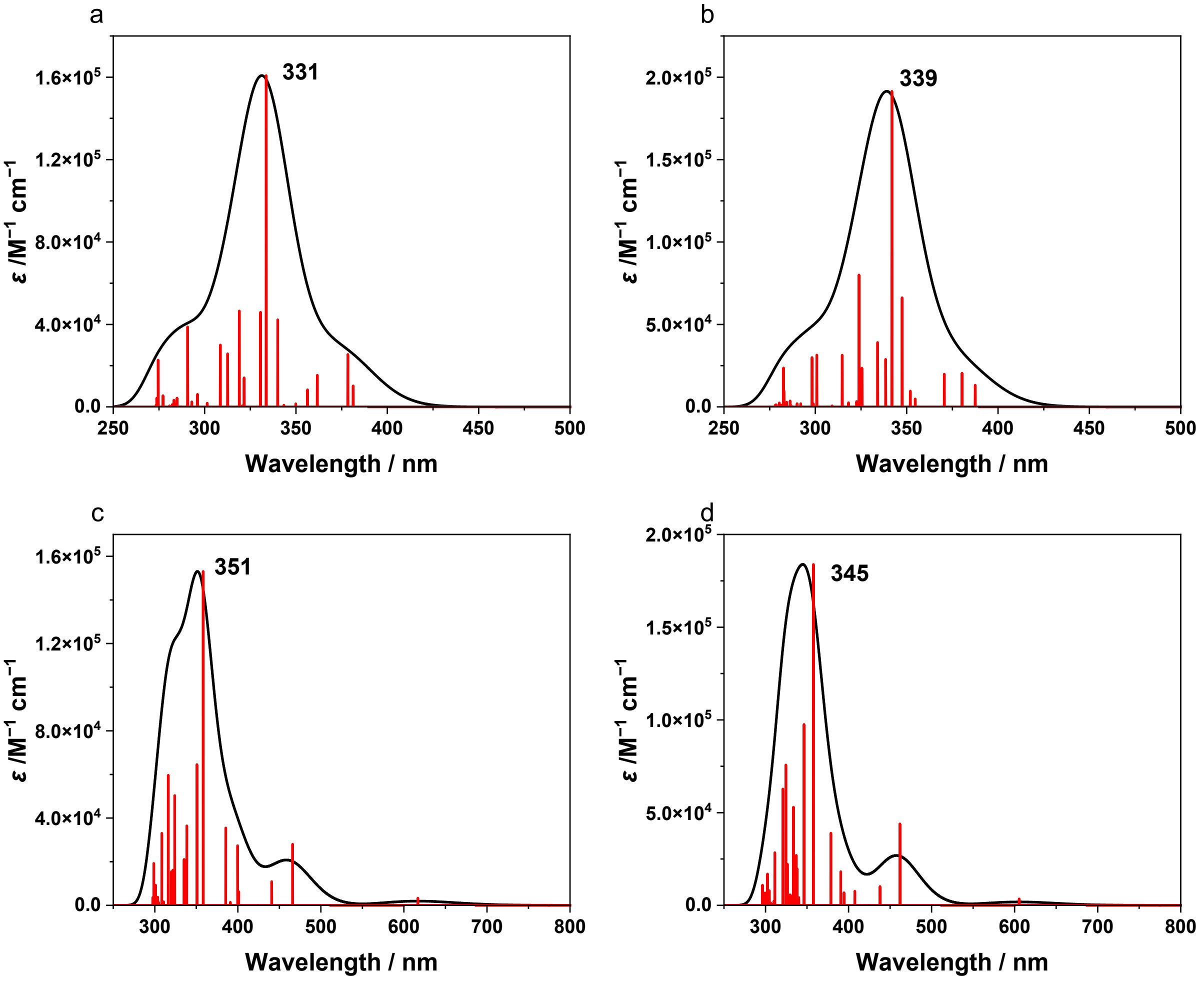

Figure S67. Calculated UV-Vis spectrum of (a) diketo[8]CPP, (b) diketo[9]CPP, (c) DBP[8]CPP and (d) DBP[9]CPP in (black plot) and calculated oscillator strengths (red bars).

Table S7. Calculated first ten transitions for diketo[8]CPP.

**Excited state**	**Energy/eV**	**Wavelength/nm**	**Oscillator strength**	**Configurations**
1	3.25	381	0.06981	H → L 91.6%
2	3.28	378	0.17293	H → L+2 45.8%<br>H → L+1 37.8%<br>H−1 → L 6.5%
3	3.43	362	0.10476	H−1 → L 59.6%<br>H−3 → L 14.9%<br>H−5 → L 10.5%<br>H → L+1 5.4%
4	3.48	356	0.05677	H → L+1 42.4%<br>H → L+2 36.9%<br>H−5 → L 5.6%<br>H−2 → L+1 5.5%
5	3.54	350	0.01064	H−2 → L 51.3%<br>H−4 → L 11.1%<br>H → L 7.0%<br>H−5 → L+1 6.5%<br>H−14 → L 6.0%
6	3.61	343	0.00558	H−5 → L 29.9%<br>H−1 → L 27.6%<br>H−3 → L 11.7%<br>H → L+2 8.3%<br>H→ L+1 8.1%
7	3.65	340	0.28633	H−1 → L+1 64.7%<br>H → L+3 9.9%<br>H−2 → L 9.5%
8	3.71	334	1.8927	H → L+3 71.1%<br>H−1 → L+2 17.5%
9	3.76	331	0.31209	H−1 → L+2 58.9%<br>H−1 → L+1 18.7%<br>H → L+3 15.4%
10	3.85	322	0.0958	H−2 → L+1 65.4%<br>H−1 → L+3 7.9%<br>H−3 → L 7.4%

Table S8. Calculated transitions for diketo[9]CPP.

Excited state	Energy/eV	Wavelength/nm	Oscillator strength	Configurations
1	3.20	387	0.83087	H → L 78.8%<br>H−2 → L 6.4%
2	3.26	380	0.12844	H → L+1 58.4%<br>H → L+2 11.3%<br>H→ L 9.8%<br>H−1 → L 8.0%
3	3.35	371	0.12529	H−1 → L 56.7%<br>H → L+1 12.0%<br>H−3→ L 11.8%<br>H−5 → L 9.7%
4	3.50	355	0.30424	H−2 → L 47.9%<br>H−5 → L 8.5%<br>H→ L+2 6.9%<br>H → L 5.9%<br>H−1→ L 5.4%<br>H−4 → L 5.3%
5	3.52	352	0.60322	H → L+2 24.1%<br>H−2 → L 17.8%<br>H−5 → L 16.8%<br>H−1 → L 7.4%<br>H−3 → L 6.5%
6	3.57	347	0.41633	H → L+2 33.6%<br>H−1 → L 13.3%<br>H−5 → L 13.3%<br>H−1 → L+1 11.2%<br>H → L+4 6.3%<br>H−3 → L 5.4%
7	3.63	342	1.20392	H−1 → L+1 41.8%<br>H → L+3 33.1%<br>H → L+2 12.5%
8	3.66	338	0.18168	H → L+4 40.7%<br>H−1 → L+1 17.1%<br>H−1→ L+2 14.7%<br>H−2 → L 6.6%
9	3.71	334	0.24587	H−1 → L+2 40.7%<br>H−1 → L+1 22.5%<br>H → L+3 14.4%

10	3.81	325	0.14741	H−2 → L+1 39.1%<br>H−1 → L+3 33.6%<br>H → L+1 7.6%

Table S9. Calculated transitions for DBP[8]CPP.

**Excited state**	**Energy/eV**	**Wavelength/nm**	**Oscillator strength**	**Configurations**
1	2.01	617	0.24044	H → L 96.8%
2	2.66	466	0.20186	H−1 → L 88.0%<br>H−2 → L 6.5%
3	2.81	441	0.78472	H−2 → L 81.6%<br>H−1 → L 7.8%
4	3.09	401	0.44909	H−3 → L 94.7%
5	3.10	400	0.19727	H → L+2 73.5%<br>H−1 → L+1 16.0%
6	3.17	391	0.10377	H−4 → L 92.4%
7	3.22	385	0.25569	H → L+1 98.0%
8	3.46	358	1.10314	H−1 → L+1 68.6%<br>H → L+2 17.5%<br>H−2→ L+1 7.7%
9	3.54	351	0.46546	H−1 → L+2 80.2%<br>H−2 → L+2 12.1%
10	3.66	338	0.26290	H−2 → L+2 50.3%<br>H−5 → L 15.3%<br>H−1 → L+2 13.1%<br>H−8 → L 5.1 %

Table S10. Calculated transitions for DBP[9]CPP.

Excited state	Energy/eV	Wavelength/nm	Oscillator strength	Configurations
1	2.05	605	0.22768	H → L 96.4%
2	2.68	462	0.28608	H−1 → L 82.6%<br>H−2 → L 12.0%
3	2.83	438	0.66434	H−2→ L 77.5%<br>H−1 → L 13.0%
4	3.04	408	0.49802	H−3 → L 94.1%
5	3.14	394	0.44388	H−4 → L 84.2%<br>H−2 → L 5.0%
6	3.17	391	0.11880	H → L+2 63.1%<br>H−1 → L+1 15.4%<br>H−4 → L 6.1%<br>H → L+1 5.4%
7	3.27	379	0.25368	H → L+1 91.5%
8	3.47	358	1.19777	H−1 → L+1 60.9%<br>H → L+2 20.7%<br>H−2→ L+1 10.8%
9	3.58	346	0.63548	H−1 → L+2 77.4%<br>H−2 → L+2 7.0%
10	3.65	340	0.27489	H−5 → L 72.6%

9.4 Calculations of Fullerene Complexes

Table S11. Uncorrected and thermal-corrected (298 K) energies of stationary points (in Hartrees) calculated at the B3LYP-D3(BJ)/6-31G(d) level of theory. [a]

Geometric Features[b]	E	E+ZPE	H	G
Diketo[8]CPP⊃C_{70} (stand)	−5281.45162	−5280.16435	−5280.08958	−5280.25224
Diketo[8]CPP⊃C_{70} (lay)	−5281.44843	−5280.16075	−5280.08645	−5280.24796
Diketo[9]CPP⊃C_{70} (stand)	−5512.52959	−5511.16130	−5511.08170	−5511.25382
Diketo[9]CPP⊃C_{70} (lay)	−5512.53400	−5511.16562	−5511.08606	−5511.25799

a) E: electronic energy; ZPE: Zero-point energy; H (=E+ZPE+E_{vib}+E_{rot}+E_{trans}+RT): sum of electronic and thermal enthalpies; G (=H–TS): sum of electronic and thermal free energies; b) These different configurations are denoted as diketo[*n*]CPPs⊃C_{70} (stand or lay), which correspond to C_{70}-lying and -standing orientations in the cavities of hosts, respectively.

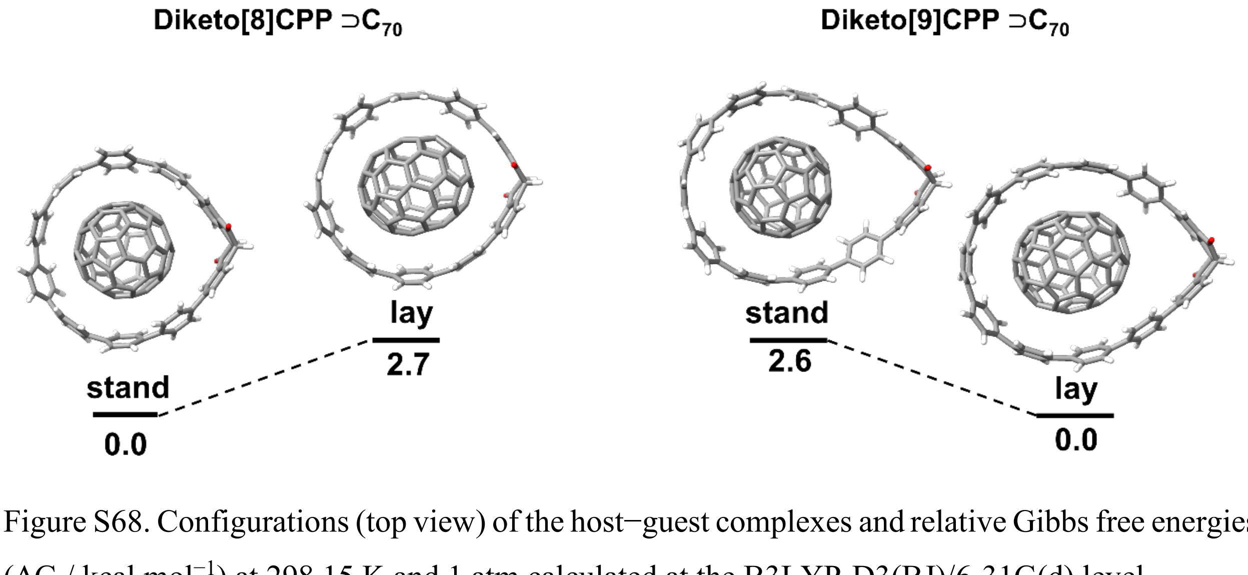

Figure S68. Configurations (top view) of the host−guest complexes and relative Gibbs free energies (ΔG / kcal mol^{-1}) at 298.15 K and 1 atm calculated at the B3LYP-D3(BJ)/6-31G(d) level.

Association free energies ΔG for the association of C_{60} and C_{70} with Diketo[n]CPPs were calculated. Structures were optimized with GFN2-xTB[31] as described above with the analytical

linearized Poisson-Boltzmann (ALPB) model with toluene solvent. Harmonic vibrational frequencies for the thermostatistical rigid-rotor-harmonic-oscillator (RRHO) contributions were calculated with GFN-FF[32], SP energies were calculated with B97-3c[33] applying the D-COSMO-RS[34] model with toluene solvent. The final ΔG_{assoc}. values consist of:

$$\Delta G_{assoc.} = \Delta\Delta G_{\text{RRHO}}(GFF) + \Delta E(\text{B97}-3\text{c}) + \Delta\delta G_{\text{solvation}}(COSMO),$$

where ΔG_{RRHO} is the thermostatistical contribution at 298K, ΔE is the gas phase association energy and $\Delta\delta G_{\text{solvation}}$ is the solvation free energy.

Table S12: Calculated association free energies. All values are given in Hartrees.

	E_{abs} (GFN2-xTB) (Toluene)	E_{ZPVE} (GFN-FF)	Δ =G_{RRHO} (GFN-FF)	E_{abs} (B97-3c) (Toluene)
Diketo[9]CPP⊃C_{60}	−309.0789621	1.272721125	1.167695065	−5128.349809
Diketo[9]CPP⊃C_{70}	−330.5983957	1.334110344	1.226433013	−5509.315267
Diketo[8]CPP⊃C_{60}	−294.1831312	1.193608387	1.093944514	−4897.400744
Diketo[8]CPP⊃C_{70}	−315.7001622	1.255526618	1.154803806	−5278.370356
Diketo[9]CPP	−180.5134032	0.906407196	0.821027244	−2843.135288
Diketo[8]CPP	−165.6171499	0.826475880	0.747133585	−2612.184827
C_{60}	−128.5026302	0.365125577	0.323213211	−2285.183442
C_{70}	−150.0123294	0.426849995	0.380131339	−2666.145778

Table S13: Calculated association free energies of the complexes.

	ΔE_{abs}(B97-3c-COSMO) / Hartrees	$\Delta\Delta G_{\text{RRHO}}$ / Hartrees	$\Delta G_{\text{assoc.}}$ / Hartrees	$\Delta G_{\text{assoc.}}$ / kcal mol^{-1}
Diketo[8]CPP⊃C_{60}	−0.032475	0.023597718	−0.008877282	−5.5
Diketo[8]CPP⊃C_{70}	−0.039751	0.027538882	−0.012212118	−7.6
Diketo[9]CPP⊃C_{60}	−0.031079	0.02345461	−0.007624390	−4.7
Diketo[9]CPP⊃C_{70}	−0.034201	0.02527443	−0.00892657	−5.6

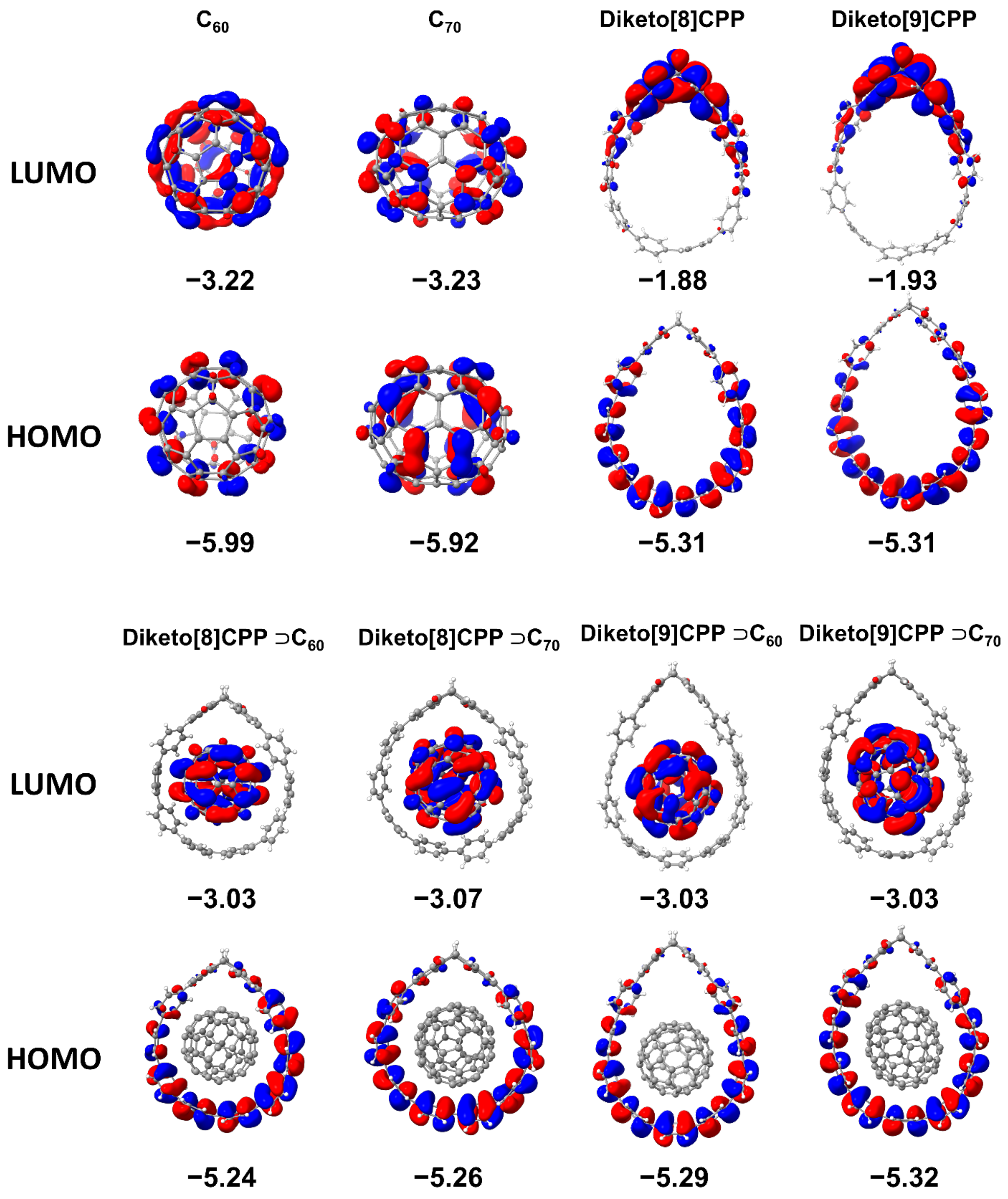

Figure S69. DFT-optimized geometries of Diketo[8]CPP⊃C_{60}, Diketo[8]CPP⊃C_{70}, Diketo[9]CPP⊃C_{60} and Diketo[9]CPP⊃C_{70} and their frontier molecular orbitals at the B3LYP-D3(BJ)/6-31G(d) level of theory.

To analyze the weak interactions between the nanohoops and fullerenes, independent-gradient-model based on Hirshfeld partition (IGMH) method calculations in Multiwfn[35] were performed. The isosurface maps were rendered using the VMD program.[21]

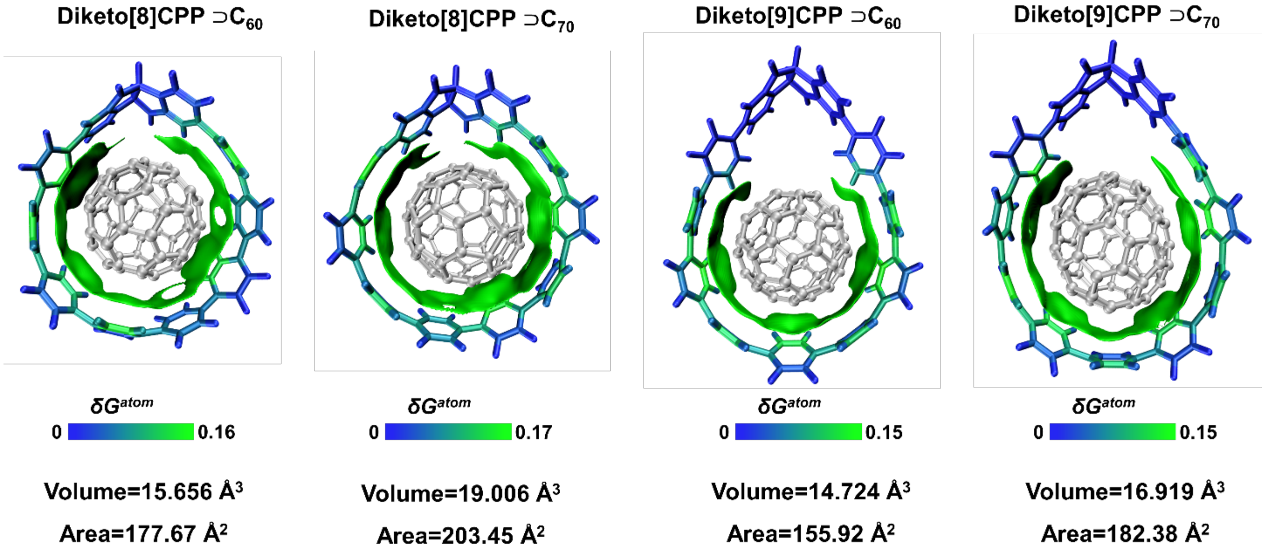

Figure S70. IGMH analysis for Diketo[8]CPP⊃C_{60}, Diketo[8]CPP⊃C_{70}, Diketo[9]CPP⊃C_{60} and Diketo[9]CPP⊃C_{70}; the green isosurfaces representing the interfragment interactions were drawn at a δg^{inter} value of 0.003; Volume and area calculated by ChimeraX, the framework of diketo[n]CPPs is colored based on the atom contribution index (δGatom) to the interfragment interactions (blue means weak contribution, and green means strong contribution).

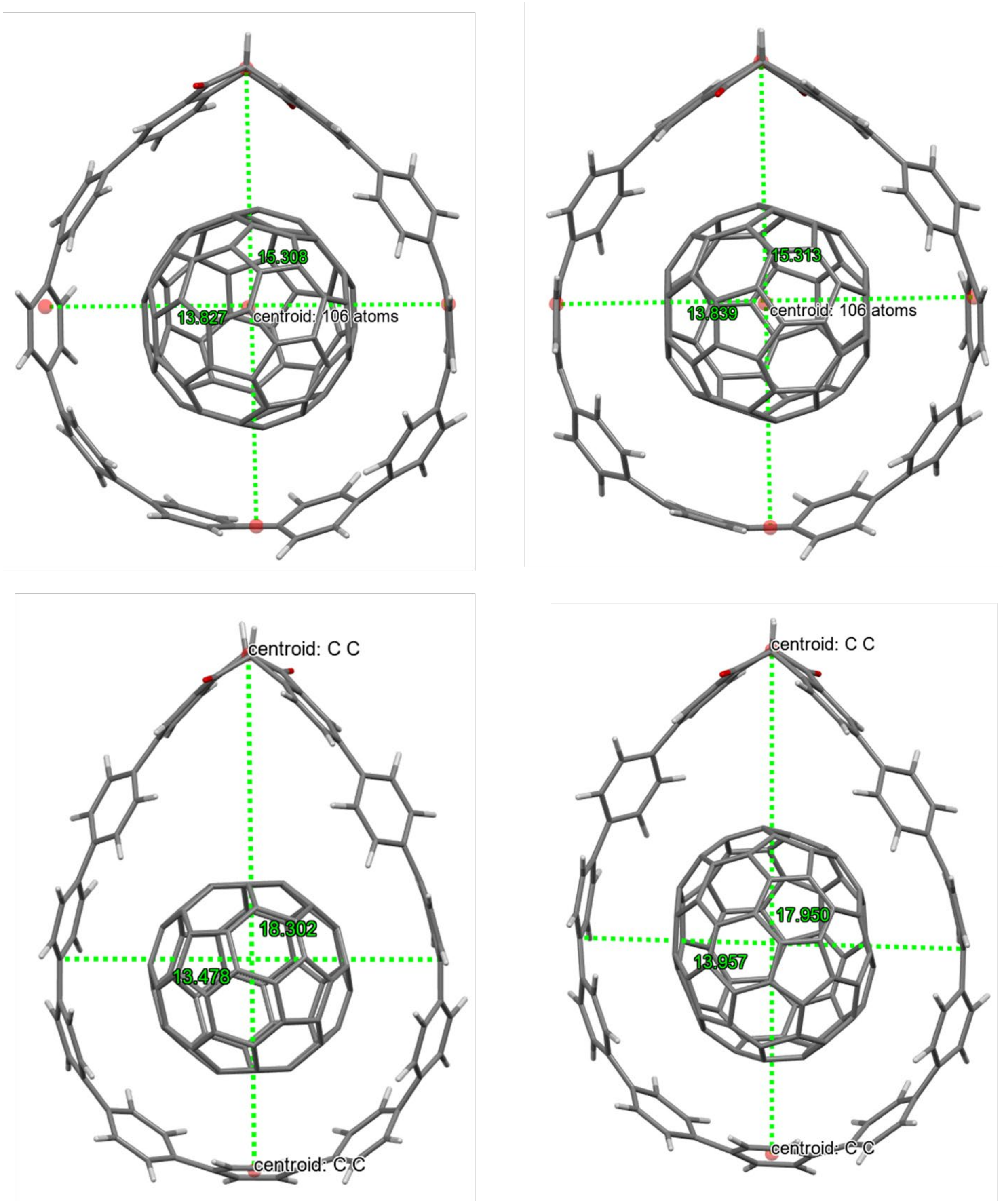

Figure S71. The shortest and longest distances of Diketo[8]CPP⊃C_{60}, Diketo[8]CPP⊃C_{70}, Diketo[9]CPP⊃C_{60} and Diketo[9]CPP⊃C_{70}.

10 References

1. H. E. Gottlieb, V. Kotlyar and A. Nudelman, *J. Org. Chem.*, 1997, **62**, 7512–7515.

2. D. Wassy, M. Hermann, J. S. Wössner, L. Frédéric, G. Pieters and B. Esser, *Chem. Sci.*, 2021, **12**, 10150–10158.

3. P. Seitz, M. Bhosale, L. Rzesny, A. Uhlmann, J. S. Wössner, R. Wessling and B. Esser, *Angew. Chem. Int. Ed.*, 2023, **62**, e202306184.

4. Q. Huang, G. Zhuang, H. Jia, M. Qian, S. Cui, S. Yang and P. Du, *Angew. Chem. Int. Ed.*, 2019, **58**, 6244–6249.

5. O. Trapp, *J. Chromatogr. B*, 2008, **875**, 42–47.

6. M. Freiberger, M. B. Minameyer, I. Solymosi, S. Frühwald, M. Krug, Y. Xu, A. Hirsch, T. Clark, D. Guldi, M. von Delius, K. Amsharov, A. Görling, M. E. Pérez-Ojeda, T. Drewello*, Chem. Eur. J.* 2023, **15**, 5665-5670.

7. P. Thordarson, *Chem. Soc. Rev.*, 2011, **40**, 1305–1323.

8. D. B. Hibbert and P. Thordarson, *Chem. Commun.*, 2016, **52**, 12792–12805.

9. Crysalispro, *1.171.43.112a*, **2024**, Rigaku OD.

10. G. M. Sheldrick, *Acta Cryst.* 2015, **A71**, 3–8.

11. G. M. Sheldrick, *Acta Cryst.* 2015, **C71**, 3–8.

12. A. L. Spek, *Acta Cryst.* 2015, **71**, 9–18.

13. D. Kratzert, I. Krossing, *J. Appl. Cryst.* 2018, **51**, 928–934.

14. C. R. Groom, I. J. Bruno, M. P. Lightfoot, S. C. Ward, *Acta Cryst.* 2016, **B72**, 171–179.

15. D. Kratzert, *FinalCif*, *V139*, https://dkratzert.de/finalcif.html.

16. Justus II, High Performance Computing for Computational Chemistry and Quantum Sciences, State of Baden Württemberg, https://www.wiki.bwhpc.de

17. TURBOMOLE V7.7 2022, a Development of University of Karlsruhe and Forschungszentrum Karlsruhe GmbH, 1989-2007, TURBOMOLE GmbH; 2007.

18. Gaussian 16, Revision C.01, M. J. Frisch, G. W. Trucks, H. B. Schlegel, G. E. Scuseria, M. A. Robb, J. R. Cheeseman, G. Scalmani, V. Barone, G. A. Petersson, H. Nakatsuji, X. Li, M. Caricato, A. V. Marenich, J. Bloino, B. G. Janesko, R. Gomperts, B. Mennucci, H. P. Hratchian, J. V. Ortiz, A. F. Izmaylov, J. L. Sonnenberg, D. Williams-Young, F. Ding, F. Lipparini, F. Egidi, J. Goings, B. Peng, A. Petrone, T. Henderson, D. Ranasinghe, V. G. Zakrzewski, J. Gao, N. Rega, G. Zheng,

W. Liang, M. Hada, M. Ehara, K. Toyota, R. Fukuda, J. Hasegawa, M. Ishida, T. Nakajima, Y. Honda, O. Kitao, H. Nakai, T. Vreven, K. Throssell, J. A. Montgomery, Jr., J. E. Peralta, F. Ogliaro, M. J. Bearpark, J. J. Heyd, E. N. Brothers, K. N. Kudin, V. N. Staroverov, T. A. Keith, R. Kobayashi, J. Normand, K. Raghavachari, A. P. Rendell, J. C. Burant, S. S. Iyengar, J. Tomasi, M. Cossi, J. M. Millam, M. Klene, C. Adamo, R. Cammi, J. W. Ochterski, R. L. Martin, K. Morokuma, O. Farkas, J. B. Foresman, and D. J. Fox, Gaussian, Inc., Wallingford CT, 2016.

19. M. D. Hanwell, D. E. Curtis, D. C. Lonie, T. Vandermeersch, E. Zurek, G. R. Hutchison, *J. Cheminform*. 2012, **4**, 17.

20. E. F. Pettersen, T. D. Goddard, C. C. Huang, G. S. Couch, D. M. Greenblatt, E. C. Meng and T. E. Ferrin, *J. Comput. Chem.*, 2004, **25**, 1605–1612.

21. W. Humphrey, A. Dalke and K. Schulten, *J. Mol. Graph.*, 1996, **14**, 33–38.

22. S. Grimme, J. G. Brandenburg, C. Bannwarth and A. Hansen, *J. Chem. Phys.*, 2015, **143**, 054107.

23. C. E. Colwell, T. W. Price, T. Stauch and R. Jasti, *Chem. Sci.*, 2020, **11**, 3923–3930.

24. A. D. Becke, *J. Chem. Phys.*, 1993, **98**, 5648–5652.

25. P. J. Stephens, F. J. Devlin, C. F. Chabalowski and M. J. Frisch, *J. Phys. Chem.*, 1994, **98**, 11623–11627.

26. R. Ditchfield, W. J. Hehre and J. A. Pople, *J. Chem. Phys.*, 1971, **54**, 720–723.

27. Y. Zhao and D. G. Truhlar, *J. Phys. Chem. A*, 2005, **109**, 5656–5667.

28. S. Grimme, J. Antony, S. Ehrlich and H. Krieg, *J. Chem. Phys.*, 2010, **132**, 154104.

29. S. Grimme, S. Ehrlich and L. Goerigk, *J. Comput. Chem.*, 2011, **32**, 1456–1465.

30. F. Weigend, *Phys. Chem. Chem. Phys.*, 2006, **8**, 1057–1065.

31. C. Bannwarth, S. Ehlert, S. Grimme, *J. Chem. Theory Comput.* 2019, **15**, 1652–1671.

32. S. Spicher, S. Grimme, *Angew. Chem. Int. Ed.* 2020, **59**, 15665–15673.

33. J. G. Brandenburg, C. Bannwarth, A. Hansen, S. Grimme, *J. Chem. Phys.* 2018, **148**, 064104

34. A. Klamt, *J. Phys. Chem.* 1995, **99**, 2224–2235

35. T. Lu and F. Chen, *J. Comput. Chem.*, 2012, **33**, 580–592
